# Structural insights enable drug discovery for the neuronal NBCn2 carbonate transporter

**DOI:** 10.1038/s41467-026-75444-4

**Published:** 2026-07-22

**Authors:** Shifan Yang, Yihan Zhao, Sezen Vatansever, Gregory Zilberg, Michael J. Capper, Jinglong Zhang, Joshua Stamos, Keino Hutchinson, Audrey L. Warren, Alexander C. Stone, Anwar Abbassi, Eric Purisic, Lap Ho, Aiqun Li, Jinye Dai, Avner Schlessinger, Bin Zhang, Daniel Wacker

**Affiliations:** 1 Department of Genetics and Genomic Sciences, Icahn School of Medicine at Mount Sinai, One Gustave L. Levy Place, New York, NY USA; 2 Mount Sinai Center for Transformative Disease Modeling, Icahn School of Medicine at Mount Sinai, One Gustave L. Levy Place, New York, NY USA; 3 Windreich Department of Artificial Intelligence and Human Health, Icahn School of Medicine at Mount Sinai, New York, New York USA; 4 Department of Pharmacological Sciences, Icahn School of Medicine at Mount Sinai, New York, New York USA; 5 Department of Neuroscience, Icahn School of Medicine at Mount Sinai, New York, New York USA; 6 AI Small Molecule Drug Discovery Center, Icahn School of Medicine at Mount Sinai, One Gustave L. Levy Place, New York New York, USA; 7 Icahn Genomics Institute, Icahn School of Medicine at Mount Sinai, New York, New York USA; 8 Present Address: The School of Theoretical and Applied Science, Ramapo College of New Jersey, Mahwah, New Jersey USA

**Keywords:** Cryoelectron microscopy, Virtual screening, Computational biophysics, Transporters

## Abstract

NBCn2 (SLC4A10), a member of the SLC4 solute carrier (SLC) family, is a sodium-dependent (bi)carbonate transporter that regulates acid extrusion in various brain regions. Mutations in NBCn2 cause severe neurodevelopmental disorders in humans, and knock out studies suggest that its role in regulating neuronal excitability could hold therapeutic potential for seizure disorders such as epilepsy. Despite its physiological importance, NBCn2’s molecular mechanisms remain largely unknown, and there is limited availability of tool compounds to further probe its role in health and disease. Combining cryoEM with computational docking and simulation studies, we herein elucidate NBCn2’s molecular architecture and substrate binding mechanisms on the atomic scale. Via structure-based drug discovery we further identify a compound series that inhibits NBCn2-mediated transport, and characterize its inhibitory mechanisms via cryoEM. Lastly, we showcase the potential of this compound series to template useful probes by demonstrating pharmacological activity both in primary culture as well as brain slices.

## Abstract (web summary)

In this work, researchers elucidate sodium and carbonate binding at the NBCn2 transporter through cryoEM, and further develop NBCn2 inhibitors that reduce neuronal activity in primary culture and brain slices using structure-based drug discovery.

## Introduction

NBCn2 or SLC4A10 is a lesser studied transporter of the SLC4 (bi)carbonate solute carrier family that regulates pH in virtually all tissues^1^. Studies showed that NBCn2 is highly expressed in the brain^2,3^, where it has been associated with the regulation of cerebrospinal fluid (CSF) production in the basolateral membrane of the choroid plexus^4^. NBCn2 knockout animals exhibit drastically reduced brain ventricular volume and neuronal excitability^2^, suggesting a key role of the transporter in brain development. In fact, several loss-of-function mutations in *slc4a10* cause severe neurodevelopmental disorders in individuals, including autism-like phenotypes and seizures during infancy^5^. These findings are mirrored by an independent study that directly links the deletion of exon 1 of *slc4a10* to autism spectrum disorder^6^.

Despite NBCn2’s critical role in human health and disease, many aspects of its (patho)physiology and function remain understudied compared to other SLC4 transporters. This is in part due to limited mechanistic insights and useful tools to elucidate and modulate its function. NBCn2 is a Na^+^-dependent HCO_3_^-^/CO_3_^2-^ transporter that is sensitive to inhibition by stilbenes^3^, a series of compounds that inhibit most SLC4 transporters but also unrelated enzymes^7^. However, there remains considerable ambiguity whether NBCn2’s Na^+^-dependent HCO_3_^-^/CO_3_^2-^ transport is in fact coupled to Cl^−^^3,8^. Furthermore, while we^9^ and others^10–13^ have determined structures of other SLC4 transporters, structural insights into NBCn2 function remain limited. It thus remains unclear how substrates such as Na^+^ and HCO_3_^-^/CO_3_^2-^ bind to NBCn2, or which residues and conformational changes are involved in substrate translocation and selectivity. But most importantly, smallmolecule NBCn2 probes to investigate the transporter’s molecular and physiological roles, or explore its therapeutic potential, are limited. For instance, NBCn2-mediated regulation of neuronal excitability remains relatively understudied, as the limited availability of small molecule NBCn2 modulators severely impedes such inquiries to genetic approaches^2,5^.

We herein report a combination of structural, computational, and drug discovery studies to address our limited mechanistic insight into NBCn2 function and the absence of useful tool compounds. We report NBCn2 structures in apo and substrate-bound states, develop a NBCn2-inhibitor series, and characterize its binding via cryoEM. Using primary neuronal culture and brain slices, we further highlight the utility and potential of these inhibitors to enable future neural studies, including inquiries into NBCn2’s role in regulating neuronal excitability and its potential as a drug target for seizure disorders.

## Results

### Structure determination of human NBCn2

**Fig. 1:**
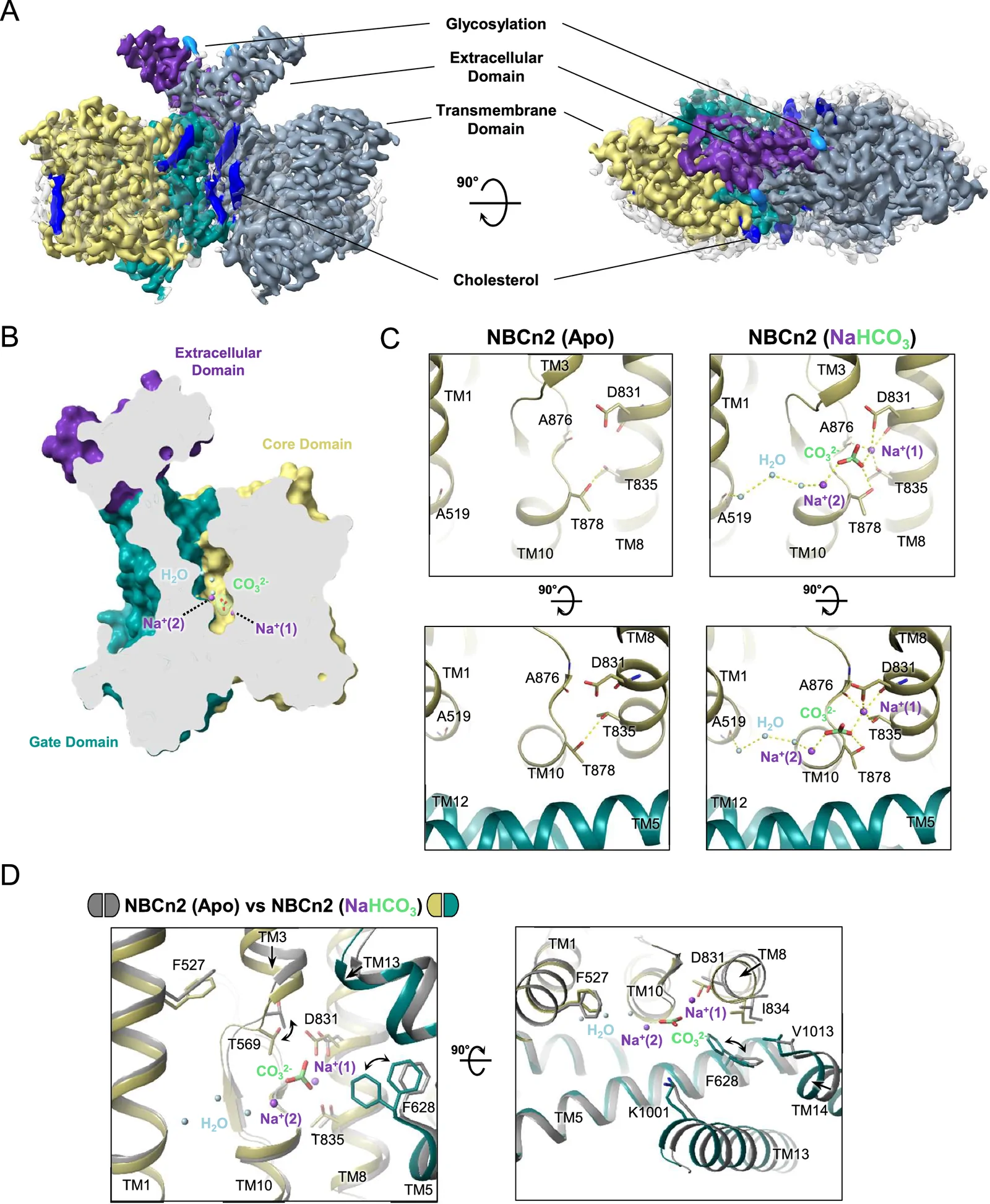
CryoEM structure of apo- and substrate-bound states reveal overall architecture of NBCn2 transporter. **A** CryoEM density of the overall NBCn2 homodimer obtained in LMNG detergent micelles. One protomer is shown in gray, the other is colored by domain: core domain (yellow), gate domain (teal), extracellular domain (purple). Cholesterols are shown in dark blue, and glycosylations are highlighted in light blue. **B** Sliced side view of substrate-bound NBCn2 protomer structure revealing binding of CO_3_^2-^ (green), Na^+^ (purple spheres), and H_2_O (light blue spheres). **C** Substrate binding pocket in apo- and substrate-bound NBCn2 structure. Hydrogen bonds and ionic interactions are shown as dashed yellow lines. **D** Overlay of apo- and substrate-bound NBCn2 structures, highlighting structural changes including two distinct F628 rotamers observed in the substrate-bound state.

To elucidate the overall architecture of NBCn2, we first determined a cryoEM structure of NBCn2 in the apo state following our previously published methodology^14^. Briefly, we expressed and purified the transporter from *Sf9* insect cells. We used a human NBCn2 construct in which we truncated residues 2-90 and 231-299 in the N-terminal domain that likely does not affect SLC4 transport function^15^, and added a C-terminal 3 C protease tag followed by a 10x His-tag to enable affinity chromatography. Following similar expression and purification protocols we established to determine structures of related SLC4 transporters^14^, we obtained a cryoEM structure of apo NBCn2 at 2.84 Å resolution (Fig. 1A, Supplementary Figs. 1, 2 and Supplementary Table 1). With core areas of the structure reaching local resolutions as high as 2.5 Å, as calculated by local resolution estimation in cryoSPARC, we characterize the overall architecture of the transporter and identify glycosylation sites and sterol binding sites.

NBCn2 assumes a typical SLC4 architecture of protomers forming a homodimeric complex, with each protomer comprising 14 transmembrane helices that can be divided into a gate and a core domain (Fig. 1A)^14^. In addition, the membrane domain contains a small extracellular domain formed by extracellular loop 3 of both protomers, which also contains N-linked glycosyl chains attached to N697. Previous studies have suggested that NBCn2 glycosylation is important for trafficking and perhaps folding^16^, but disrupting glycosylation of other SLC4 transporters did not affect their function^17,18^. We further observe several densities for likely membrane components near membrane-facing surfaces, some of which correspond to cholesterol molecules that are found bound to both gate and core domains. While we also observe densities in the interface between the protomers that likely correspond to phospholipids, these are much less defined than in our previous structures of anion exchanger 1 (AE1/SLC4A1)^14^, and we therefore opted not to model them. As observed in our previous AE1 structure^14^, NBCn2’s intracellular N-terminal domain appears largely disordered, and our discussion herein will thus exclusively focus on the C-terminal membrane domain that conducts ion transport in SLC4 transporters^19–21^. Overall, our NBCn2 structure appears very similar to previously reported structures of other Na^+^-coupled SLC4 transporters such as NDCBE (SLC4A8)^10^ and NBCe1 (SLC4A4)^11^, with more evident differences compared to mechanistically divergent transporters such as AE1 (SLC4A1)^14,22^ and BTR (SLC4A11)^12^ (Supplementary Fig. 2C).

### Structural elucidation of NBCn2 substrate binding

To characterize modulatory surfaces and delineate the molecular details of substrate binding, we next determined a cryoEM structure of NBCn2 co-purified with NaHCO_3_ at 2.42 Å resolution (Fig. 1B, Supplementary Figs. 3, 4 and Supplementary Table 1). Comparison to other SLC4 transporter structures reveals that both structures of apo and substrate-bound NBCn2 display features of the typical SLC4 outward-facing (OF) state (Supplementary Fig. 5A). In the OF state, we observe a large cavity formed between the gate- and core domain in each protomer that extends from the extracellular space to an anion binding pocket at the center of the protein (Fig. 1B). The cavity is largely open and lined by positively charged residues with a negatively charged environment at the center of the transporter (Supplementary Fig. 5B). There, we observe a largely conserved SLC4 substrate pocket architecture: The N-terminal ends of TM3 and TM10 point towards each other from the extracellular and intracellular direction, respectively, forming a gap lined with residues that contribute to coordinating the substrate (Fig. 1C). As seen in other SLC4 transporter structures, for NBCn2 we also observe two juxtaposed beta strands that constrain the binding site laterally in the core domain^22,23^. Within this architecture, we find that the NBCn2 structure obtained in the presence of NaHCO_3_ reveals densities likely corresponding to both Na^+^ and HCO_3_^-^/CO_3_^2-^ ions, as well as water molecules. Based on the signal strength and size of the densities we observe two Na^+^ ions coordinating a CO_3_^2-^ ion, with additional water molecules forming a network between transporter and ions (Fig. 1C). One of the Na^+^ ions (hereafter named Na^+^(1)) is coordinated by the sidechains of D831^TM8^ and T835^TM8^ at the N-terminal end of TM8, and the backbone carbonyl groups of D831^TM8^ and A876 near the beta strand extending from the N-terminal end of TM10 (Fig. 1C). These interactions range between 2.3–2.5 Å, which is characteristic for Na^+^-Oxygen distances observed in structures^24^. Tightly bound by the transporter, Na^+^(1) forms an ionic interaction with the anion at a distance of 3.1 Å. In line with previous work^10^, as well as the presence of two Na^+^ ions, we interpret the anion density as a divalent CO_3_^2-^ ion (Fig. 1C). Positionally, this CO_3_^2-^ ion is located at the end of the presumed ion entry tunnel closer to the gate domain (Fig. 1B). There, it forms only one notable direct interaction with NBCn2, specifically with the backbone nitrogen of T878^TM10^ at the N-terminal end of TM10, and interacts with a second Na^+^ ion termed Na^+^(2), which coordinates CO_3_^2-^ from the opposite side as Na^+^(1). It should be noted that unlike Na^+^(1), Na^+^(2) appears to be more indirectly coordinated by the transporter via a water-based network, though we observe similarly strong densities for both Na^+^ in our maps (Supplementary Fig. 4).

To validate the identity and binding sites of both Na^+^ and CO_3_^2-^ ions, we next performed several computational experiments. First, we performed Site Identification by Ligand Competitive Saturation (SILCS) simulations^25^, which combine Grand-Canonical Monte Carlo (GCMC) and Molecular Dynamics (MD) protocols (Supplementary Table 2). SILCS simulation approaches have been used for other SLC4 transporters to map the potential anion and cation binding sites^10,26^. Our SILCS-generated fragment maps of NBCn2 reveal putative cation and anion binding sites (Supplementary Fig. 6A). Specifically, the cation mesh precisely coincides with D831^TM8^ and is proximal to T835^TM8^, matching the Na^+^(1) binding site observed in the NBCn2 cryoEM map. The anion map suggests the presence of an anion binding pocket formed by TM3, TM5, TM10, and TM13, which closely aligns with the binding site of CO_3_^2-^ in NBCn2. The unbiased SILCS simulations indicate that Na^+^(1) is anchored by the local chemical environment and contributes to CO_3_^2-^ binding, whereas Na^+^(2) appears more indirectly bound by a local water-based network. Moreover, MD simulations suggest that Na^+^(1), Na^+^(2), and CO_3_^2-^ did not dissociate and remained stable in the substrate binding site during the simulations (Supplementary Fig. 6B). Taken together, the SILCS and MD simulations therefore provide additional evidence for both the identity and positioning of the ions modeled in our cryoEM structure.

We next analyzed our structure using the CheckMyMetal server^27^, indicating that Na^+^(1) is appropriately coordinated, while Na^+^(2) has a low valence value (Supplementary Fig 7A). This is likely due to the absence of density for additional ligands of Na^+^(2) beyond a CO_3_^2-^ and a water molecule, suggesting that additional water ligands are less ordered in our structure. We further used AlphaFold3 (AF3)^28^ to predict the structure of the NBCn2 monomer in complex with two sodium ions, one carbonate ion, and three water molecules (Supplementary Fig. 7B–E). We find that the predicted protein structures were consistent with RMSD values clustering around ~ 0.6 Å, and AF3 correctly placed Na^+^(1) and CO_3_^2-^, with less precise placement of Na^+^(2), which was driven by some clear outliers (Supplementary Fig. 7B–E). Protein-ligand interaction analysis using PLIP^29^ and distance measurements further validates that the AF3 models, which largely mirror our experimental structure, produced coordination distances within optimal ranges (Supplementary Fig. 7B–E). Moreover, we used AF3 to predict ion coordinates in the absence of anions and water molecules (Supplementary Fig. 7F, G). AF3 generally placed Na⁺(1) near its experimental position. In contrast, Na⁺(2) was rarely predicted near its experimental site under these conditions, with many predictions showing large displacements, indicating that AF3 struggles to localize this ion without an anion and water. Together, these computational studies support our initial hypothesis that Na^+^(2) binding in NBCn2 is mediated primarily by solvent and anion interactions.

When compared with the NBCn2 apo structure, we observe several structural changes in the transporter both on the scale of repositioning of helices, as well as changes of sidechains (Fig. 1D and Supplementary Fig. 5C). The most apparent difference is an overall “contraction” of the substrate binding sites, in which many of the helices of the gate and core domains appear to move closer to each other, on the order of approximately 1-2 Å. Overall, the binding of Na^+^ and CO_3_^2-^ ions results in a 24.9% reduction in the volume of the substrate binding site compared to that in the apo state (933-996 Å^3^ vs 1,327 Å^3^) (Supplementary Fig. 5D). When comparing apo- and substrate-bound states, we observe several conformational differences in the sidechains of key residues (Fig. 1D). Concomitantly with a contraction of the substrate binding pocket, we observe that D831^TM8^, I834^TM8^, K1001^TM13^, V1013^TM14^, and T569 move closer towards the ion substrates. The shift of I834^TM8^ and V1013^TM14^ appears to push on the sidechain of F628^TM5^, for which we observe two distinct side chain rotamer conformations (Fig. 1D). The 1.4-1.5 Å downward movement of TM8 towards the substrate is accompanied by a 2.4 Å downward swing of T569 in the loop connecting the end of the helix to one of the beta sheets that laterally borders the ion pocket. It should be noted that even subtle changes in D831^TM8^ are potentially very important for substrate recognition, as the acidic residue in this position has been highlighted as a key ion selectivity filter for distinct transporter-specific substrates in other SLC4 transporters^30^.

### Comparison of distinct substrate configurations across SLC4 transporters

**Fig. 2:**
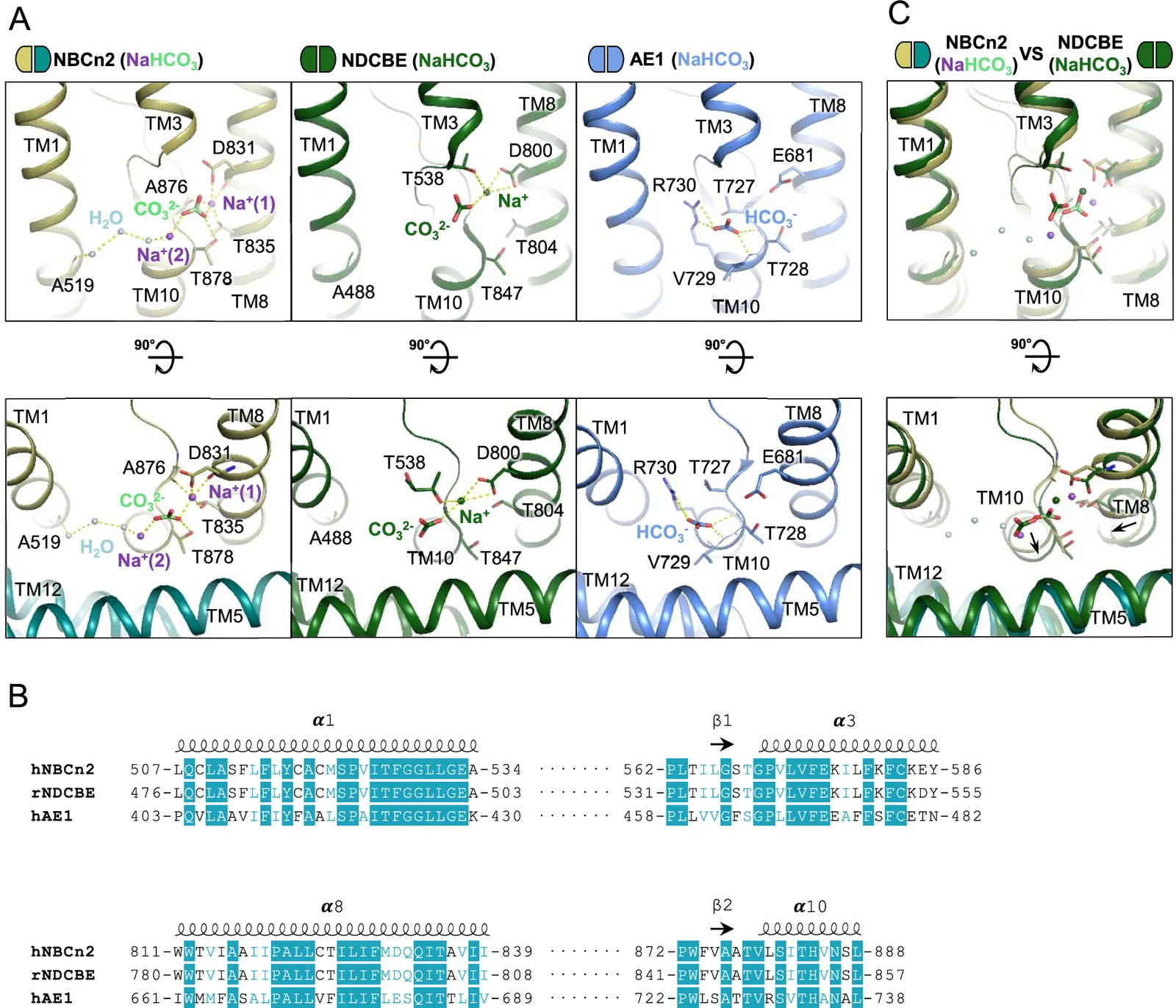
Distinct ion stoichiometries in the substrate binding pockets of different SLC4 transporters. **A** Comparison of ion substrate-binding pockets in different substrate-bound SLC4 structures, including human NBCn2 (yellow and teal), rat NDCBE (dark green), and human AE1 (blue). Structures also show CO_3_^2-^ (green), Na^+^ (purple spheres), and H_2_O (light blue spheres), and hydrogen bonds and ionic interactions are shown as dashed yellow lines. **B** Sequence alignment of the substrate binding pocket residues in different SLC4 transporters. Conserved residues are highlighted in teal, and similar residues are shown in teal font color. **C** Overlay of substrate-binding pockets for sodium-dependent NBCn2 and NDCBE reveals key differences that likely reflect their distinct mechanisms. Coloration is the same as in (**A**).

When compared to structures of other substrate-bound SLC4 transporters in the OF state, we observe several notable differences (Fig. 2). First, comparison to the substrate binding pocket described in the structure of the related Na^+^-dependent SLC4 transporter NDCBE (SLC4A8) from rat^10^ shows a similar positioning of CO_3_^2-^, which is also linked to the transporter via a Na^+^ ion corresponding to Na^+^(1) in our structure. However, the NDCBE structure does not reveal a second Na^+^, which is potentially due to its distinct mechanism as a Na^+^-dependent CO_3_^2-^/Cl^-^ exchanger^31^. Comparison to our previous structure of the substrate-bound HCO_3_^-^/Cl^-^ exchanger AE1^14^ shows a structurally different ion configuration in which HCO_3_^-^ is primarily anchored by an arginine sidechain in AE1’s TM10, a role that appears to be fulfilled by Na^+^(1) in NBCn2 (Fig. 2A). Overall, these observations further highlight the importance of Na^+^ for electroneutral NBCn2-mediated transport as CO_3_^2-^ (i) is coordinated by two Na^+^ ions, (ii) is in close proximity to the acidic sidechain of D831^TM8^, and (iii) doesn’t exhibit any strong direct interactions with the transporter. Conversely, while we observe strong ion densities in the substrate-bound state, we do not observe any Na^+^ density in the apo state, even though the apo sample was prepared in the presence of 150 mM Na^+^ (compared to 250 mM Na^+^ in the substrate-bound sample (see methods)). This observation suggests that while Na^+^ is likely critical for the binding of CO_3_^2-^, this ion conversely appears to be critical for the binding of Na^+^ ions, including Na^+^(1). In agreement with previous studies reporting on NBCn2’s electroneutral transport that does not require Cl^-8^, our findings suggest that NBCn2 potentially co-transports one CO_3_^2-^ ion with two Na^+^ ions, though it is also conceivable that fewer Na^+^ ions are translocated alongside CO_3_^2-^. While the molecular basis for the distinct ion configurations in NBCn2 and NDCBE remains elusive, it should be noted that despite near identical residue composition in their respective substrate binding pockets (Fig. 2B), NBCn2 and NDCBE exhibit potentially important conformational differences (RMSD = 0.847 Å) (Fig. 2C).

### Structure-based discovery of NBCn2 modulators

**Fig. 3:**
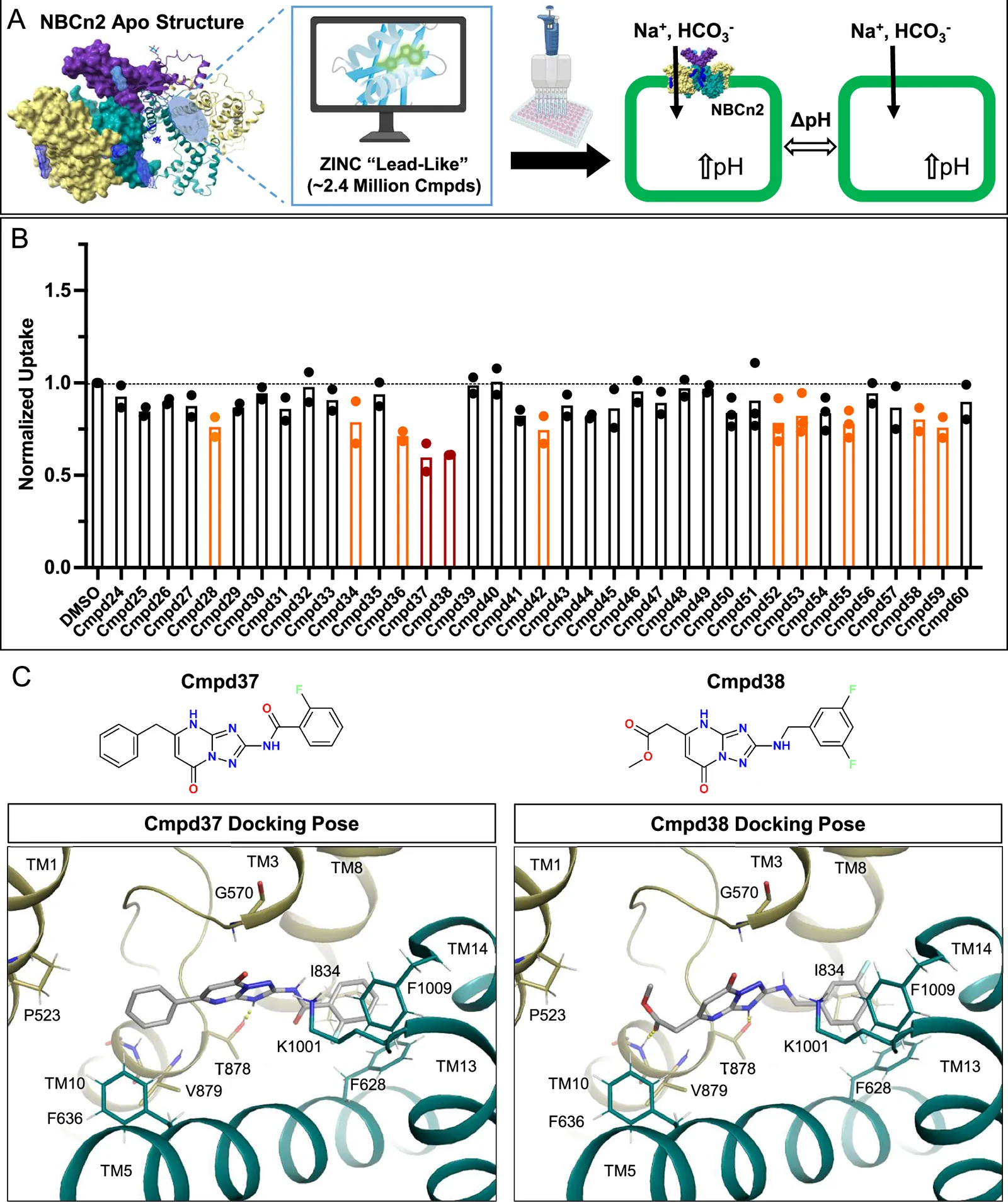
Virtual ligand screening identifies small molecule inhibitors of NBCn2. **A** Schematic of the structure-based ligand discovery workflow in part created in BioRender. Zhang, J. (2026) https://BioRender.com/27c86pl. Virtual ligand screening using the apo-NBCn2 structure was used to identify potential binders of the NBCn2 substrate binding pocket, which were tested in a HEK-cell based NBCn2 transport assay. **B** 37 compounds (Cmpd24-Cmpd60) were tested at 100 μM for their ability to inhibit NBCn2-mediated transport of 25 mM NaHCO_3_ in HEK cells. Data were normalized against vehicle (DMSO), and reveal a reduction of substrate transport for 11 compounds (orange and red bars). All experiments were performed with 3-4 technical repeats, and are averaged from *n* = 2 (Cmpd24-49, Cmpd56-60), *n *= 3 (Cmpd50-55), or *n* = 4 (DMSO) independent experiments. The two compounds with the largest reduction in transport (red bars) were validated by re-synthesis of the original compounds. **C** 2D chemical structures of Cmpd37 (Compound37) and Cmpd38 (Compound38), and docking poses of the compounds from the virtual ligand screen in the binding site of NBCn2. NBCn2 domains are colored in yellow (core domain) and teal (gate domain), and compounds are colored by element with carbons colored in gray. Source data are provided as a Source Data file.

Several studies point to a role of NBCn2 in regulating neuronal excitability, and different reports have thus linked the transporter to epilepsy and Alzheimer’s disease^2,5,32^. However, NBCn2’s role and therapeutic potential as a drug target in these pathologies remains underexplored, not at least due to the notable absence of useful tool compounds to precisely modulate its function. There is a striking dearth of useful probes for SLC4 transporters in general, prompting us previously to rationally develop an SLC4-targeted inhibitor series for AE1^14^. As for NBCn2, only the non-selective pan-SLC4 inhibitor DIDS has been reported as a transport modulator^3^, though its use is very limited due to its similar activity at most other SLC4 transporters^1^ and even non-SLC proteins^7^. To address the limited availability of tools to study NBCn2 function, we adopted our previous approach to develop NBCn2 inhibitors, leveraging our structural data in structure-based ligand discovery (Fig. 3). Empowered by our recent successes at AE1^14^ and our high-resolution structural data on the NBCn2 substrate binding site in apo and substrate-bound states, we aimed to generate compounds targeting the substrate binding site. Accordingly, we docked a virtual library of 2.4 million molecules from the ZINC15 “Lead-Like” subset (https://zinc15.docking.org/) against the substrate binding site in our apo NBCn2 structure (Fig. 3A). We used Glide of the Schrödinger suite to perform virtual screening^33–35^, and visually inspected the top-scoring molecules for docking artefacts to select compounds for experimental testing^36^. Out of these we curated a subset of 37 compounds (named Cmpd24-60) that was purchased for experimental testing (Supplementary Table 3). Before compounds could be experimentally tested for NBCn2 modulation, we set up a NBCn2 uptake assay in 96-well format to enable rapid screening of multiple compounds (Fig. 3A and Supplementary Fig. 8A). In brief, uptake was determined in HEK cells stably expressing NBCn2 upon tetracycline induction. NBCn2-mediated uptake was determined as the pH difference between induced and uninduced cells following Na^+^-dependent CO_3_^2-^ transport. After optimization, we screened all 37 compounds at 100 µM for their ability to modulate transport of NaHCO_3_ and discovered a range of inhibitory activities (Fig. 3B). Among the 37 compounds tested, 11 compounds showed an inhibition of NaHCO_3_ uptake when compared to vehicle (DMSO). The most efficacious inhibitory effect was observed for compound 37 (40.4% uptake reduction) and compound 38 (39.0% uptake reduction) in this series, which both contain similar triazolopyrimidine core scaffolds (Fig. 3C). To the best of our knowledge, no rationally developed inhibitor scaffold has been reported for NBCn2, and both compounds are notably distinct from the stilbene inhibitor DIDS as determined by low Tanimoto coefficients of 0.071 (compound 37) and 0.061 (compound 38). The docking poses of compounds 38 (Cmpd38) and compound 37 (Cmpd37) indicate that the triazolopyrimidine scaffold likely binds to the core substrate binding site between the ends of TM3 and TM10, and that the different substituents extend towards the entry of the anion binding cavity and a pocket bordered by TM8, TM14, and TM5 residues, respectively (Fig. 3C).

### SAR of NBCn2 Inhibitor Series

**Fig. 4:**
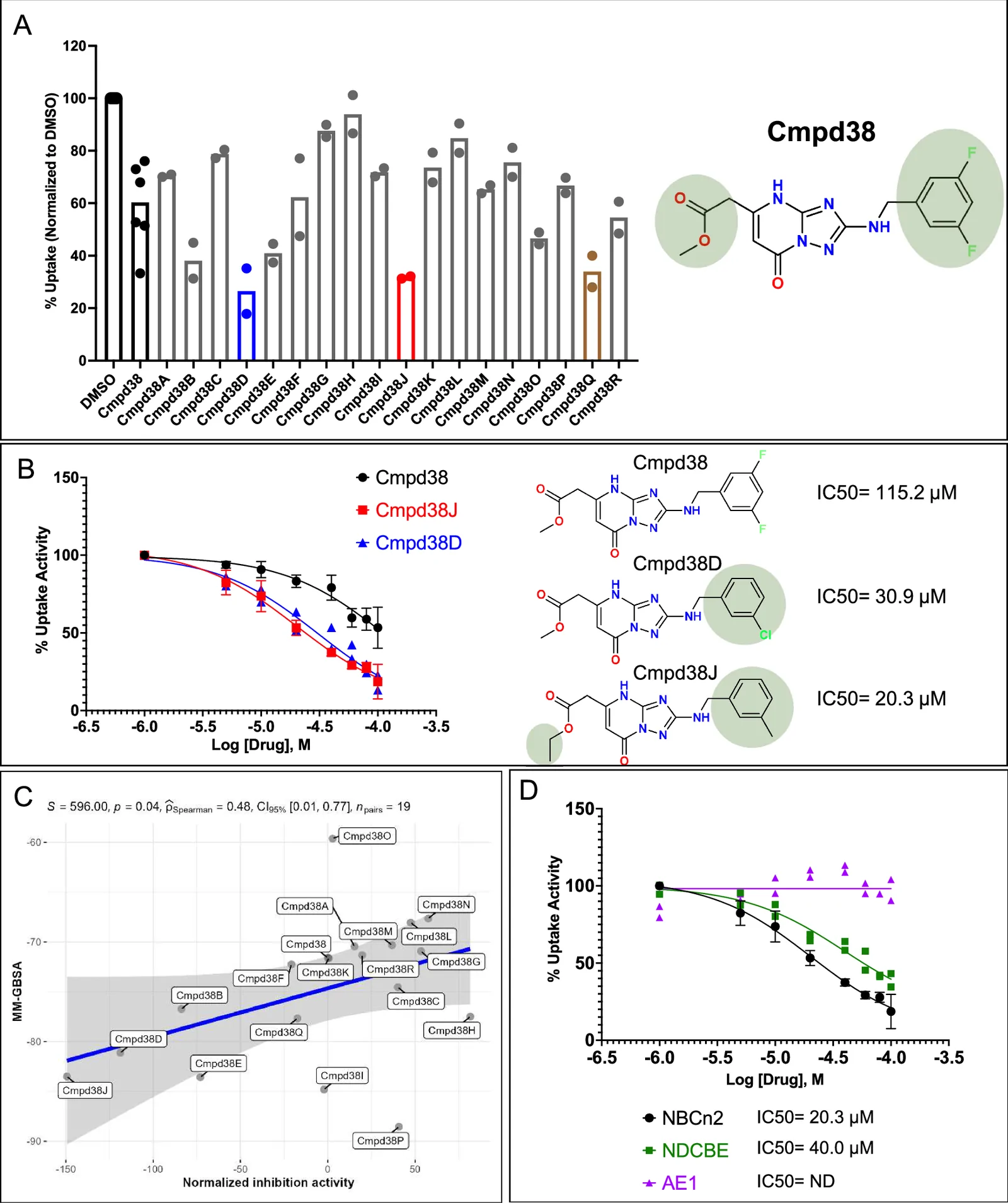
Structure-activity-relationship studies lead to improvement of inhibitor potency. **A** Analogs of Cmpd38 were tested at 100 μM concentration in the HEK-cell based NBCn2 uptake assay. Experiments were performed with 3 technical repeats and are averaged from *n* = 2 independent experiments (Cmpd38A-R) or *n* = 6 independent experiments (DMSO, Cmpd38). Data were normalized to DMSO. Cmpd38D (blue), Cmpd38J (red), and Cmpd38Q (brown) show the strongest inhibitory activity compared to DMSO and Cmpd38. 2D chemical structure of Cmpd38 with green circles highlights moieties that vary between Cmpd38 and its analogs. **B** Concentration-response studies of Cmpd38, Cmpd38D, and Cmpd38J enable estimations of IC50 values. Experiments were performed with 4 technical repeats and are averaged from *n* = 2 (Cmpd38D) or *n* = 3 (Cmpd38, Cmpd38J) independent experiments. Data are represented as mean ± s.d and have been normalized to Cmpd38. 2D chemical structures of the compounds highlight their chemical differences. **C** Cmpd38 analog predicted binding affinities ranked according to their MM-GBSA calculations (y-axis) are plotted against their inhibitory activities determined in uptake experiments at 100 μM concentration (x-axis). Correlation was assessed using two-sided and nonparametric Spearman’s rank correlation analysis (*ρ* = 0.48, *p* = 0.04, *n* = 19 compounds). No multiple-comparison correction was applied. The blue line represents the fitted trend, and the gray shaded area indicates the 95% confidence interval of the fit. **D** Concentration-response studies of Cmpd38J at NBCn2, NDCBE, and AE1. Experiments were performed with 4 technical repeats and are averaged from *n* = 3 (NBCn2) or *n* = 2 (NDCBE, AE1) independent experiments. Data are represented as mean ± s.d and have been normalized. Source data are provided as a Source Data file.

Since even concentrations as high as 100 µM of either Cmpd37 or Cmpd38 do not fully inhibit NBCn2 transport (Fig. 3B), we next opted to improve inhibitory potency and generate structure-activity-relationship (SAR) data through testing commercially available compound analogs selected based on their predicted interactions with the NBCn2 binding site (Fig. 4). Based on the more robust inhibitory activity of Cmpd38, we focused on optimization of this lead scaffold. Since the docking pose suggested that Cmpd38’s triazolopyrimidine core is anchored between TM3 and TM10, which are also critical for substrate binding (Fig. 1), we elected to test analogs with different triazolopyrimidine substituents. A curated set of 18 commercially available compounds (Cmpd38A-R) was purchased (Supplementary Table 4) and experimentally tested at 100 µM. We observed that 6 analogs showed an increased inhibitory effect, 9 showed reduced inhibition, and 3 analogs appeared to show similar effects compared to the parent compound (Fig. 4A and Supplementary Fig. 8B). Specifically, we observed that replacing the aromatic difluoro-phenyl with a cyclopentyl group reduces inhibition (Cmpd38P). Keeping the aromatic phenyl group, but replacing the difluoro substituents with either a single methyl (Cmpd38B and Cmpd38J) or a single chloride (Cmpd38D) substituent increased inhibitory efficacy at 100 µM compared to that of Cmpd38. These improvements might be explained by favorable hydrophobic contacts with the sidechains of F628^TM5^, I834^TM8^, and F1009^TM14^ (Fig. 3C). We next performed concentration-response experiments for select compounds, and found that several analogs indeed show increased apparent potencies compared to Cmpd38, with Cmpd38D and Cmpd38J exhibiting the most improved IC50s of 30.9 µM and 20.3 µM, respectively (Fig. 4B). We docked the 18 analogs and Cmpd38 to the substrate binding site of NBCn2, and estimated the relative binding affinities with molecular mechanics generalized Born surface area (MM-GBSA) calculations, which cannot predict absolute affinity/potency values accurately, but can rank similar compounds more precisely than docking score. We observe a weak but significant correlation between the ligands’ predicted relative binding affinities and their inhibitory activities (Fig. 4C). Interestingly, compounds 38J, 38D, 38B, 38E, 38F, which exhibit efficacious inhibitory effects and predicted binding affinities, all retain their triazolopyrimidine core and (m)ethyl-propionate tail, while substituting the di-fluoride for one halogen or methyl group. Lastly, we wanted to assess whether our most potent compound, Cmpd38J, can also inhibit other SLC4 transporters. We therefore performed similar concentration response assays testing the compound’s inhibitory activity at the mechanistically related transporter NDCBE (SLC4A8), and the sodium-independent anion exchanger AE1 (SLC4A1) (Fig. 4D). We find that Cmpd38J does not appear to affect AE1’s uptake activity, but does inhibit NDCBE with an apparent IC50 of 40.0 µM. Though this appears less than Cmpd38J’s IC50 of 20.3 µM at NBCn2, it should be noted that potentially transporter-dependent substrate Km values, and the non-physiological nature of the assay, prevent accurate determination of Cmpd38J’s affinities/potencies at the individual transporters.

### CryoEM elucidates inhibitor binding at NBCn2

**Fig. 5:**
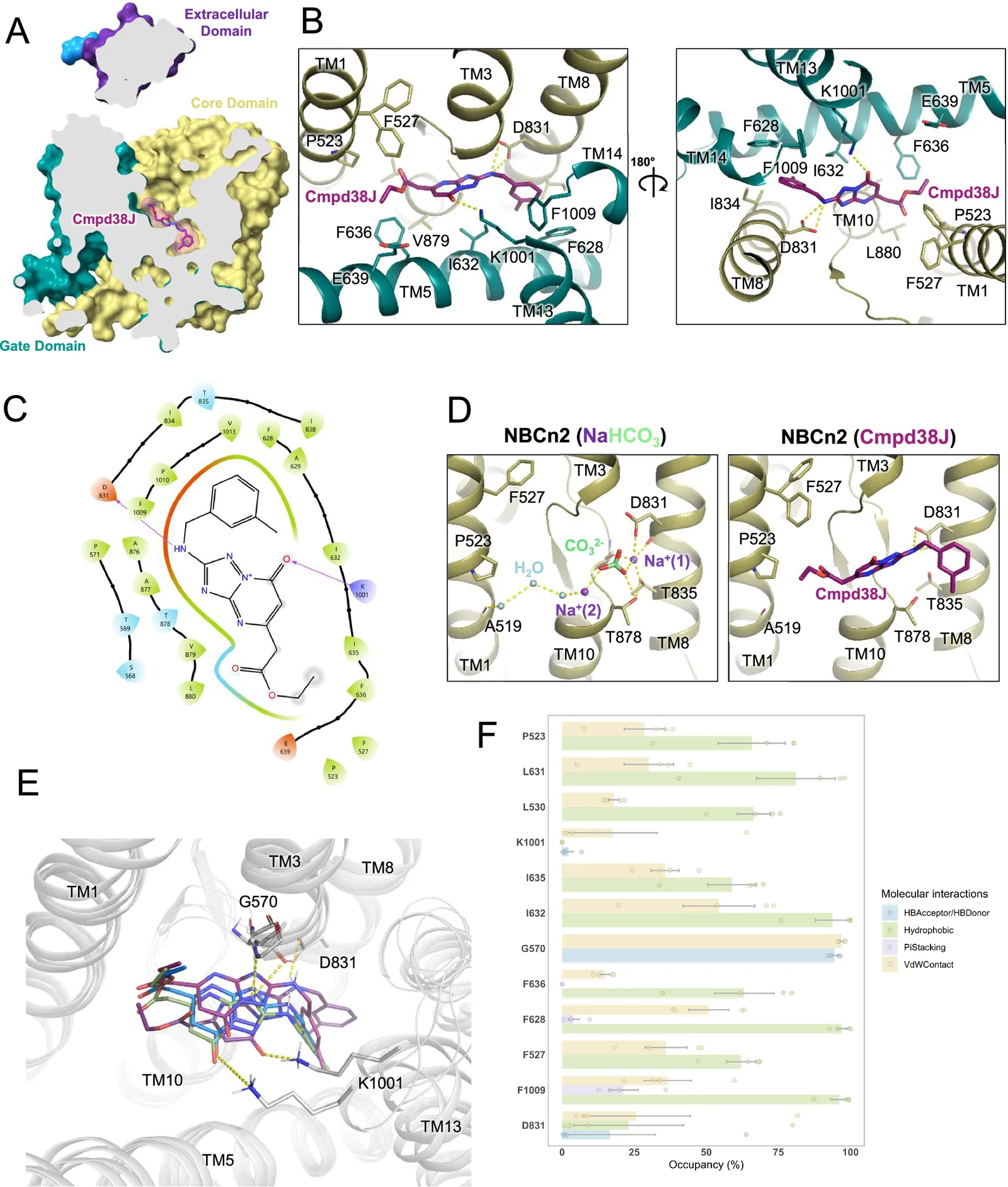
CryoEM and MD simulations elucidate binding mode of herein developed NBCn2 inhibitor. **A** Sliced side view of Cmpd38J-bound NBCn2 cryoEM structure showing gate domain (teal), core domain (yellow), extracellular domain (purple) and Cmpd38J (magenta). **B** Close-up of the Cmpd38J (Compound 38J) binding site. Dashed yellow lines denote hydrogen bonds and/or ionic interactions between Cmpd38J and NBCn2 residues, and F527 is shown in both rotamer states identified in the structure of Cmpd38J-bound NBCn2. **C** 2D schematic of molecular interactions between Cmpd38J and NBCn2 generated using the Schrödinger suite. Magenta arrows highlight polar interactions between Cmpd38J and NBCn2 residues. **D** Structural comparison of Cmpd38J- and substrate-bound NBCn2, with the same coloration as in Fig. 1C and panel (**B**). **E** Representative Cmpd38J poses from the clustering analysis of MD simulations with four replicas. 4 representative cluster centers are shown. Dashed yellow lines denote polar interactions between Cmpd38J and NBCn2 residues. **F** Molecular interactions between Cmpd38J and distinct NBCn2 residues derived from aggregated 1 μs MD simulations. The X-axis denotes occupancy (%), and values represent mean ± s.e.m from 4 independent replicas. Source data are provided as a Source Data file.

We next determined a cryoEM structure of NBCn2 bound to Cmpd38J, to validate our docking studies and obtain insights into how this compound series binds and inhibits the transporter (Fig. 5 and Supplementary Figs. 9, 10). This was done by adding 100 µM Cmpd38J to purified NBCn2 before grid preparation. At 3.24 Å global resolution, 2.9 Å around the substrate binding pocket, we obtained clear density for the inhibitor in the substrate binding pocket (Supplementary Fig. 10A). Overall, we observe the same OF state as seen in the apo- and substrate-bound states (Figs. 1, 5A). Cmpd38J’s triazolopyrimidine core is located between the tips of TM3 and TM10, thus overlapping with the binding of the CO_3_^2-^ and Na^+^ ions observed in our structure of substrate-bound NBCn2. The scaffold is accommodated between the transporter’s core and gate domains, and held in place by multiple hydrophobic and hydrophilic interactions with both domains. For instance, we observe an interaction between Cmpd38J’s carbonyl group and K1001^TM13^, as well as its amino group and D831^TM8^ - the aspartate coordinating Na^+^(1) in the structure of substrate-bound NBCn2 (Fig. 5B–D). Overall, this binding pose is reminiscent of how AE1 is bound by DIDS and H_2_DIDS, which are inhibitors that compete with substrate binding^14^. Cmpd38J’s amino-linked methyl-phenyl- and ethyl-propionate group extends from the core scaffold, and the inhibitor thereby approximately traces the presumed substrate entry channel (Fig. 5A–D). The methyl-phenyl group binds towards the cytoplasmic side within a pocket of TM5, TM8, and TM14 residues, forming hydrophobic contacts with the sidechains of F628^TM5^, I834^TM8^, and F1009^TM14^. This NBCn2 pocket is similar to that of AE1, accommodated by niflumic acid, a translocation inhibitor that does not compete for substrate binding in AE1^14^. The ethyl-propionate group, on the other hand, is located towards the extracellular substrate entry near TM1 and TM5 residues. This area is much less constrained, and we only observe weaker contacts with P523^TM1^ and F636^TM5^. Interestingly, we observe ambiguous density for F527^TM1^ that is best modeled by two distinct rotamer states, one of which forms hydrophobic contacts with Cmpd38J’s ethyl-propionate moiety, resulting in the binding pocket varying in size between 1181 Å^3^ and 1291 Å^3^ (Fig. 5B–D and Supplementary Fig. 10B, C). In the apo and substrate-bound states, the F527^TM1^ sidechain exclusively points towards the center of the core domain “behind” TM3, but Cmpd38J binding appears to stabilize a partial flip into the presumed ion entry channel. This partial rearrangement and the binding in a comparatively open cavity suggest that the ethyl-propionate does not form strong interactions with the transporter. Overall, Cmpd38J binding results in an overall contraction of the substrate binding site compared to the apo state (1181-1291 Å^3^ vs 1,327 Å^3^), while the inhibitor bound site is slightly larger than the substrate-bound site (1181-1291 Å^3^ vs 933-996 Å^3^) (Supplementary Figs. 5D, 10B, C).

We further performed MD simulations to visualize the stability of Cmpd38J in the binding site, describe key interacting residues, and thereby identify a path forward for ligand optimization in future work (Fig. 5E, F and Supplementary Table 2). During the simulation, Cmpd38J remains stable in the observed binding site (Supplementary Fig. 11A, B). Clustering analysis of four simulation replicas reveals that the most consistent polar interaction is the hydrogen bond between Cmpd38J and the backbone nitrogen of G570^TM3^, which appears in the three most populated clusters. Interestingly, Cmpd38J’s carbonyl group demonstrates potential to form a salt bridge with the side chain of K1001^TM13^; however, this was only observed in the second and fourth most populated clusters. Additional molecular interactions include pi-pi stacking with F628^TM5^ and hydrogen bonding with T569^TM3^ and D831^TM8^, respectively. During MD simulations, we also observe that Cmpd38J’s interaction with NBCn2 is predominantly mediated by hydrophobic and van der Waals contacts, particularly through interactions with hydrophobic residues within TM1 and TM5 (Fig. 5F and Supplementary Figs. 10, 11). Cmpd38J maintains hydrophobic and pi-stacking interactions with the aromatic residues F1009^TM14^ and F628^TM5^ through its methyl-phenyl group. We observe that G570^TM3^ has the potential to play a key role in Cmpd38J binding, with G570^TM3^’s backbone nitrogen acting as a hydrogen bond acceptor (Fig. 5E, F). In addition, the ethyl-propionate tail of Cmpd38J extends into a hydrophobic subpocket in the core domain, characterized by P523^TM1^ and F527^TM1^.

We next set out to validate the importance of select Cmpd38J-NBCn2 interactions by testing the inhibitory activity of the compound at alanine mutants of F628, I632, D831, I834, K1001, and F1009 (Supplementary Fig. 12). However, out of the 6 residues tested, all but the K1001A mutant effectively abolished substrate transport, and uptake activity of K1001A was reduced compared to wildtype NBCn2 (Supplementary Fig. 12A, B). Curiously, 25 µM Cmpd38J appeared to more efficaciously inhibit transport at the K1001A mutant compared to what was observed at wildtype NBCn2. This observation is in partial agreement with our MD studies, which show a K1001-Cmpd38J salt bridge in only 6.13% of the total simulation frames and suggest that this interaction is not critical for compound binding (Fig. 5F and Supplementary Fig. 11C). To validate this finding, we further performed Cmpd38J concentration response studies at the K1001A mutant and find that Cmpd38J shows a considerably increased apparent potency at the K1001A mutant (IC50 = 5.3 µM) compared to wildtype (IC50 = 20.3 µM) (Supplementary Fig. 12D). These data appear to suggest that the K1001 side chain might sterically interfere with Cmpd38J binding, or compromise hydrophobic areas of the binding pocket, thereby overall weakening the interaction with Cmpd38J. However, we note that a K1001A mutation could also affect substrate transport, thereby increasing the apparent potency of substrate transport inhibition. Lastly, we performed enzyme-linked immunosorbent assays (ELISA) that validated that changes in substrate transport or Cmpd38J inhibition were not due to altered NBCn2 expression caused by the mutations (Supplementary Fig. 12C). Taken together, these studies identify key interactions between the inhibitor and the transporter, and provide a blueprint for rationally improving inhibitory potency in future work.

### Probing NBCn2 activity in primary culture and brain slices using Cmpd38J

The finding that Cmpd38J inhibits NBCn2 in HEK cells overexpressing the transporter strongly highlights that this scaffold may be used to develop useful and more potent probes to investigate the transporter’s mechanisms, physiology, and potential as a drug target. However, before we initiate labor and resource-intensive studies to generate highly potent probes, we herein first wanted to validate that Cmpd38J-based probes can be used to investigate NBCn2-linked function in more physiologically relevant models.

**Fig. 6:**
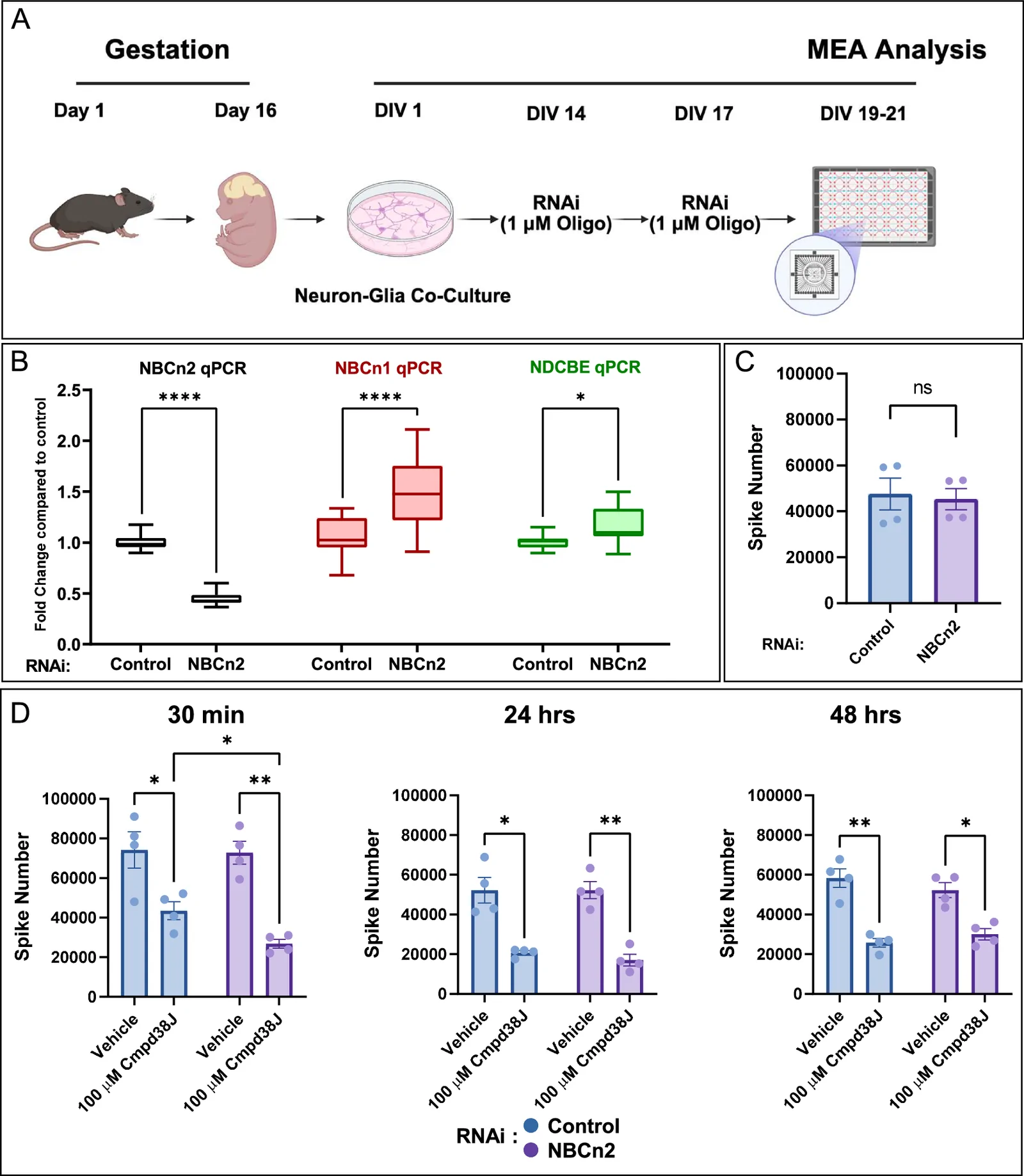
NBCn2 inhibitor reduces neuronal excitability in primary neuronal culture. **A** Schematic illustrating the generation of primary neuron-glia co-culture from mouse embryos and recording spontaneous neuronal activity via multi-electrode array (MEA) analysis following two consecutive treatments with control (scramble) or NBCn2 antisense oligos. Created in BioRender. Zhang, J. (2026) https://BioRender.com/bxoajzx. **B** Quantitative PCR (qPCR) showing NBCn2, NBCn1, and NDCBE mRNA expression after NBCn2-Knockdown (KD) via two consecutive additions of 1 µM NBCn2 antisense oligos (1–3 technical repeats; *n* = 5 independent samples). Data are normalized to control and displayed as box-and-whisker plots (center line = median; bounds = 25th/75th percentiles; whiskers = min/max). Two-way analysis of variance (ANOVA) with Sidak’s post hoc test: NBCn2 knockdown reduces NBCn2 (****P < 0.0001) and increases NBCn1 (****P < 0.0001) and NDCBE (*P = 0.0250) mRNA levels. **C**,** D** Spontaneous neuronal activity (spike counts over 10 min) recorded by MEA following NBCn2-KD (violet) or control (blue), with vehicle (DMSO) or 100 µM Cmpd38J (Compound38J). Neurons were from E16 C57BL/6 J mouse embryos; data shown as mean ± s.e.m. with individual data points. **C** Baseline neuronal activity after antisense oligo treatment (*n* = 4 independent samples from 9 embryos; 16 wells/plate, 64 total wells/group). No significant difference (ns) between groups (two-sided unpaired* t* test, *P* = 0.8012). **D** Neuronal activity from the same wells as in (**C**) split into Cmpd38J or vehicle treatment (32 wells/group; 8 technical replicates; *n* = 4 independent samples). Two-way ANOVA with Fisher’s LSD post hoc test revealed significant activity reduction between several conditions. 30 min - Control:Vehicle vs Control:Cmpd38J (*P = 0.0121); NBCn2-KD:Vehicle vs NBCn2-KD:Cmpd38J (**P = 0.0043); Control:Cmpd38J vs NBCn2-KD:Cmpd38J (*P = 0.0227). 24 h - Control:Vehicle vs Control:Cmpd38J (*P = 0.0131); NBCn2-KD:Vehicle vs NBCn2-KD:Cmpd38J (**P = 0.0075). 48 h - Control:Vehicle vs Control:Cmpd38J (**P = 0.0010); NBCn2-KD:Vehicle vs NBCn2-KD:Cmpd38J (*P = 0.0240). Source data are provided as a Source Data file.

Several studies reported on the expression of NBCn2 in the brain^3,37^, where the transporter appears to regulate neuronal excitability in the hippocampus, likely through its role as an acid extruder^2,5^. To first validate these observations in primary culture, we used an established in vitro murine brain neuron-glia co-culture model to explore the potential effect of NBCn2 down-regulation. Specifically, we isolated brain tissue from embryonic day 16 C57BL/6 J mouse embryos, prepared neuron-glia co-culture according to established protocols^38^, and plated cells onto multi-electrode array (MEA) plates to monitor spontaneous neuronal activity (Fig. 6A). We confirmed expression of NBCn2, NDCBE, and NBCn1 in our culture via qPCR, and set up a knockdown protocol using two separate treatments with 1 µM NBCn2 antisense oligos at DIV14 and DIV17 that reduces NBCn2 expression levels by ~ 50% at DIV19 (Fig. 6B). Strikingly, we found that NBCn2 knockdown appears to increase expression levels of NBCn1 by ~ 50% and NDCBE by ~ 30% compared to control conditions treating neurons with control oligos. Next we monitored spontaneous neuronal activity on DIV19 using the MEA assay, and observed no significant changes in spike number in NBCn2 knockdown cells compared to cells treated with control oligos (Fig. 6C). Next we added 100 µM Cmpd38J or vehicle (DMSO) to both neurons treated with NBCn2 antisense oligos or control oligos on DIV19, and measured spontaneous neuronal activity 30 min, 24 h, or 48 h post drug or vehicle treatment (Fig. 6D). We observe considerably reduced spike numbers in Cmpd38J treated versus vehicle treated neurons across all time points, and regardless of whether NBCn2 was knocked down or not. Strikingly, we further note an acute additive effect of Cmpd38J treatment and NBCn2 knockdown 30 min following drug treatment, which is no longer observed 24 h and 48 h after drug treatment. Finally, we determined the viability of the cells after drug treatment and data collection, and validated that none of the observed effects were due to the toxicity of the treatments (Supplementary Fig. 13). The neuronal activity data recorded 30 min after drug treatment, in particular, thus suggest a tentative link between NBCn2 inhibition and neuronal activity in our cellular model system (see Discussion). But our studies at the same time strongly argue for more comprehensive future studies to probe the utility of Cmpd38J-based compounds in interrogating NBCn2- as well as NDCBE- and NBCn1-linked biology.

**Fig. 7:**
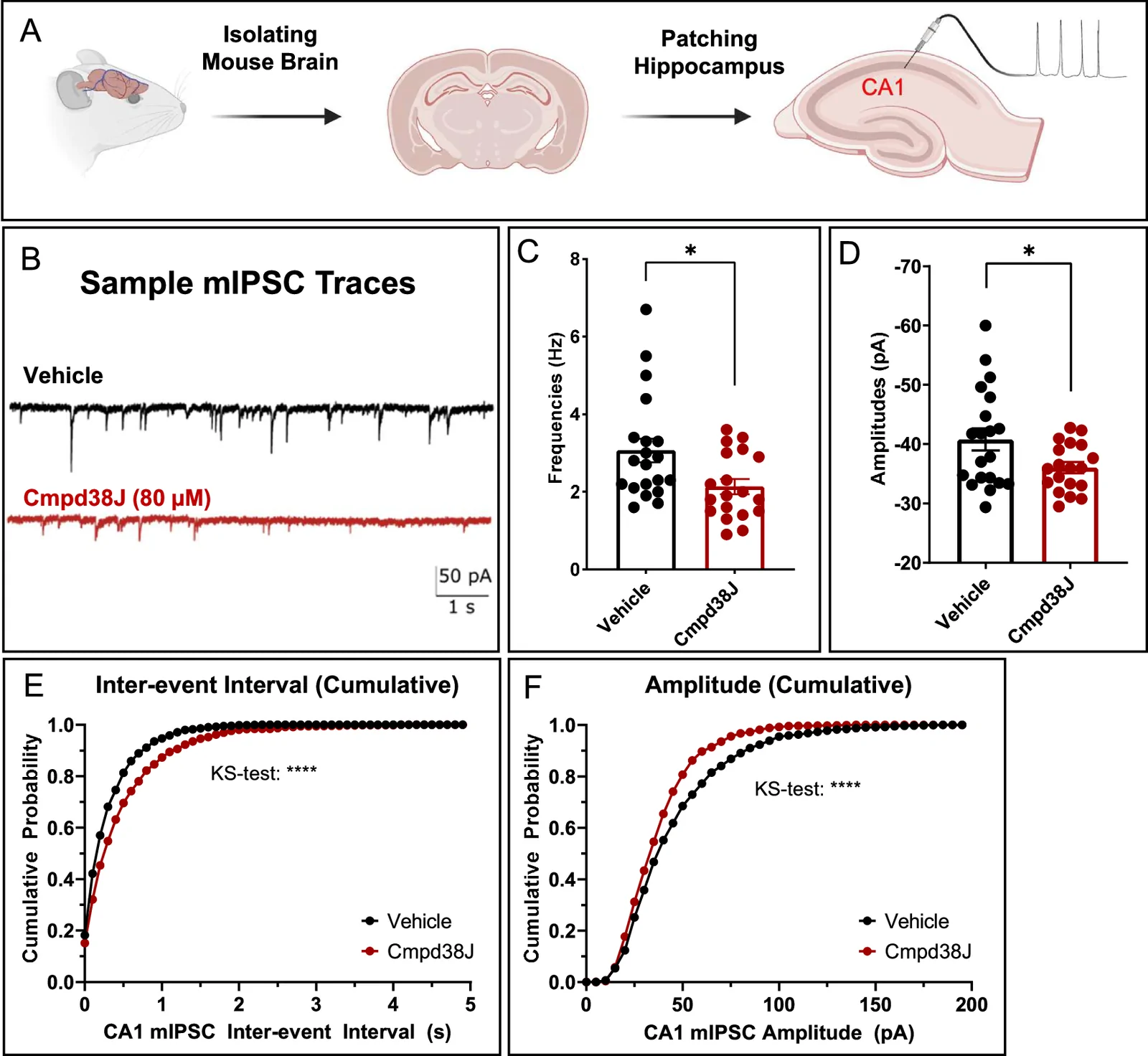
NBCn2 inhibitor reduces neuronal excitability in brain slices. **A** Schematic illustrating electrophysiological recordings done in the CA1 region of hippocampal slices from three male C57BL/6 J mice. Created in BioRender. Zhang, J. (2026) https://BioRender.com/18ab91m. **B** Sample traces of miniature inhibitory postsynaptic currents (mIPSCs) determined in aCSF supplemented with vehicle (black) or 80 µM Cmpd38J (Compound38J; red). **C**, **D** Average spike frequencies (**C**) and amplitudes (**D**) of mIPSCs recorded over 1 minute from cells in the presence of vehicle (black) or 80 µM Cmpd38J (red). Data denote mean ± s.e.m. We recorded neuronal activity from *n* = 20 (vehicle condition) and *n* = 19 (drug condition) independent cells across 3 brain slices. Reduction in spike frequencies (*P = 0.0148) and amplitudes (*P = 0.0304) was determined via a two-sided unpaired *t* test. **E**, **F** Cumulative probability plots of mIPSC intervals (**E**) and inter-event amplitudes (**F**) were generated from the first 50 events per cell in the presence of vehicle (black) and 80 µM Cmpd38J (red). Differences between vehicle and drug conditions were assessed using the Kolmogorov–Smirnov test (****P < 0.0001). Source data are provided as a Source Data file.

We next investigated Cmpd38J’s utility in studies done on brain tissue (Fig. 7). Previous studies highlighted that disruption of the *slc4a10* gene can protect from seizures^2^ and reduce neuronal activity^5^. NBCn2 could thus be a potential drug target for epilepsy and other seizure disorders, but structural changes in the brain associated with NBCn2 knockout^2^ obfuscate whether inhibition of NBCn2 function causes or contributes to the observed effects on neuronal signaling. Since NBCn2 knockout was shown to directly impair GABAergic neurotransmission in the hippocampus^5^, we set out to test whether Cmpd38J-mediated NBCn2 inhibition can similarly reduce inhibitory signaling (Fig. 7A–F). To this end, we performed electrophysiological recordings of miniature inhibitory postsynaptic currents (mIPSCs) on mouse brain slices in the presence and absence of 80 µM Cmpd38J. In brief, following previous studies of NBCn2^5^, brain slices were prepared from three male C57BL/6 J mice, and mIPSCs were recorded in the CA1 region of the hippocampus (Fig. 7A). Compared to vehicle, Cmpd38J treatment reduces both the frequencies and amplitudes of mIPSCs in mouse hippocampus (Fig. 7B–F), providing further evidence that NBCn2 inhibition indeed impairs neurotransmission at inhibitory synapses as observed in knockout animals^5^. We note, however, that Cmpd38J-mediated activity at other SLC4 transporters (Fig. 4D) requires the development of even more selective NBCn2 probes in future studies.

Although our functional studies in primary culture and brain slices are not aimed at comprehensively investigating NBCn2-linked physiology, our pharmacological studies nonetheless suggest that NBCn2-mediated transport is required for normal neuronal signaling. But more importantly, these studies validate the utility of our lead scaffold in interrogating NBCn2-linked biology and disease, and form a strong chemical foundation for developing optimized probes in future work. Such probes are urgently needed to further interrogate NBCn2’s molecular and physiological functions, and explore the tractability of this and related transporters as bona fide targets to treat seizure disorders, autism, and other neurological illnesses.

## Discussion

Through combined cryoEM and computational studies, we herein provide insight into NBCn2 substrate binding and contrast it to that of other SLC4 transporters. Under our conditions, we observe two Na^+^ ions and one CO_3_^2-^ ion in the canonical SLC4 substrate binding site^10,11,14,22,26^, which is distinct from that of other SLC4 transporters such as AE1^14^ or NDCBE^10^, and likely reflects transport mechanisms unique to NBCn2^8^. AE1 is a HCO_3_^-^/Cl^-^ exchanger^39^ and binds a single HCO_3_^-^ or Cl^-^ ion^40^, while NDCBE was reported to mediate Na^+^-driven CO_3_^2-^/Cl^-^ exchange, and cryoEM revealed one Na^+^ and one CO_3_^2-^ ion bound to NDCBE^10^. By contrast, NBCn2’s transport mechanisms remain ambiguous: Early studies suggested that NBCn2 exchanges Na^+^ and HCO_3_^-^ for H^+^ and Cl^- 3^, and other work indicated that NBCn2 co-transports two HCO_3_^-^ ions for each Na^+^ ion, thus requiring Cl^-^ exchange to retain electroneutrality^41^. However, independent studies suggest that NBCn2 mediates Cl^-^ self-exchange that is not coupled to Na^+^ or HCO_3_^-^/CO_3_^2-^ co-transport^8^. While our studies show two Na^+^ ions and one CO_3_^2-^ ion bound to the substrate binding pocket in the outward facing state, it remains unknown whether both or just one sodium ion is translocated during a transport cycle. That being said, our observation of a single bound CO_3_^2-^ ion does not agree with the previously proposed 1:2 Na^+^:HCO_3_^-^ transport ratio^41^, which would require two consecutive transport cycles with a combined translocation of a single Na^+^ ion. It should be noted that we do not observe any structural evidence for Cl^-^ ions in either the apo or substrate-bound NBCn2 states. It therefore remains to be seen what role Cl^-^ plays in NBCn2 mediated HCO_3_^-^/CO_3_^2-^ transport, given the different hypotheses. From a molecular point of view, this will require obtaining structures of additional NBCn2 transport states, and determination of structures in the presence of distinct ion compositions and concentrations.

Despite NBCn2 loss of function causing severe neurological disorders^5^, the transporter remains notably understudied compared to other SLC4 transporters, and there exist no useful compounds to investigate NBCn2’s role in human health and disease. We thus herein leveraged our structural data to generate NBCn2 modulators and initiated the development of useful probes, resulting in the discovery of a scaffold that inhibits NBCn2-mediated NaHCO_3_ transport. CryoEM revealed the binding mode of the most potent compound, Cmpd38J, which traces the ion entry channel in NBCn2 and binds the same core substrate binding pocket as Na^+^ and CO_3_^2-^. These observations indicate that Cmpd38J directly competes with ion binding, but also restricts access to the core substrate binding pocket as a channel blocker. MD simulations further suggest that Cmpd38J’s methyl-phenyl moiety tightly binds a pocket formed by TM5, TM8, TM13, and TM14, whereas the ethyl-propionate moiety exhibits considerable flexibility in the substrate access channel. The positioning of Cmpd38J’s methyl-phenyl moiety is thus similar to how the drug niflumic acid binds to AE1^14^, which does not affect substrate binding but does inhibit conformational changes associated with substrate translocation^14,42^. Accordingly, findings from our NBCn2-Cmpd38J cryoEM structure indicate that the methyl-phenyl group contributes to the compound’s inhibitory activity. Together, our structural insights suggest that Cmpd38J inhibits NBCn2 transport via (i) channel blockage^43^, (ii) substrate competition^44^, and (iii) translocation inhibition^42^, which are three distinct mechanisms that have previously been observed for individual AE1 inhibitors^14^.

We further observe inhibitory activity of Cmpd38J at NDCBE but not AE1, which is not surprising as the structurally highly conserved substrate binding sites of NBCn2, NDCBE, and NBCn1 can likely bind the inhibitor in a similar fashion. By contrast, the mechanistically distinct AE1 anion exchanger possesses a distinct substrate binding pocket architecture which likely precludes Cmpd38J binding. Overall, it therefore appears that our compound series likely possesses pan-inhibitory activity at NBCn2, NDCBE, and NBCn1, but the precise selectivity as well as activity at other sodium-dependent SLC4 transporters will need to be tested in future studies. Overall, our combined cryoEM and SAR studies of different Cmpd38J analogs in HEK cells provide a molecular blueprint to develop Cmpd38J into improved probes with increased potencies. For instance, we hypothesize that modification or substitution of the flexible ethyl-propionate moiety could substantially stabilize and strengthen transporter interactions. Using a similar structure guided approach, combined with cryoEM or docking studies of NDCBE and NBCn1, will similarly likely lead to probes with increased target selectivities.

In HEK cells Cmpd38J inhibits NBCn2 with an estimated IC50 of ~ 20 µM in the presence of 25 mM NaHCO_3_. While this is similar to previously observed drug IC50s at AE1^14^, we note that this assay is non-physiological, and it is difficult to assess true affinities and potencies. We nonetheless reasoned that the inhibitor’s estimated IC50 is sufficient to test and validate the use of this scaffold in physiological models of NBCn2 function. Studies have shown that NBCn2 functions as an acid extruder in the brain to control neuronal excitability^2,5,45,46^, and we thus tested the effect of NBCn2 knockdown and Cmpd38J treatment in both primary neuronal culture as well as brain slices. We observe that NBCn2 knockdown did not have any apparent effect on neuronal activity in primary murine neuron-glia co-culture as measured by MEA electrophysiology. We note that NBCn2 knockdown appears to simultaneously increase NBCn1 and NDCBE expression as determined by qPCR. Conceivably, increased NBCn1 and NDCBE expression could therefore represent adaptive changes that allow the neurons to compensate for reduced NBCn2 expression, ultimately preserving neuronal activity. Curiously, while Cmpd38J appeared to robustly reduce neuronal activity measured up to 48 hrs following treatment, we did observe an additive inhibitory effect of NBCn2 knockdown and Cmpd38J inhibition, 30 min following drug treatment. We surmise that Cmpd38J’s inhibitory activity at NBCn2, NDCBE, and likely NBCn1 unmasks NBCn2 knockdown-mediated effects on neuronal activity. However, the dissipation of these additive effects at 24 and 48 h is likely owed to further adaptive changes, which need to be studied in future work.

In brain slices, we find that Cmpd38J-mediated inhibition reduces mIPSCs in the hippocampus, recapitulating findings from NBCn2 knockout animals^5^. This suggests that loss of NBCn2 transport might contribute to previously observed effects on GABAergic signaling, which do not appear to be solely driven by NBCn2 knockout-related changes in brain structure^2,5^. However, these studies are complicated by Cmpd38J’s inhibitory effects on NDCBE and likely NBCn1. That being said, previous studies reported that NDCBE appears to primarily modulate glutamatergic signaling and NDCBE knockout does not appear to impact GABAergic signaling^47^. The same studies also showed that NDCBE knockout increases seizure latencies, thus possibly conferring anti-epileptic activity. Similarly, while NBCn1 has been found in GABAergic and glutamatergic neurons^48^, it was shown that NBCn1 knockout protects from glutamate-mediated neurotoxicity^49^. These studies highlight the complexity and challenge of studying the role of distinct SLC4 transporters in regulating brain function, which are further complicated by our finding that even knockdown of a single transporter likely leads to extensive adaptations and compensatory changes. However, we would also like to emphasize that Cmpd38J and similar transporter-selective and non-selective inhibitors are key to elucidating SLC4 function and (patho)physiology.

In light of studies that show that deletion of either NBCn2, NDCBE, or NBCn1 increases seizure thresholds^2,5,47–49^, it is conceivable that a pan-inhibitor such as Cmpd38J might be more efficacious in increasing seizure thresholds compared to any transporter-selective inhibitor. At the very least, these studies strongly argue for the exploration of targeting NBCn2 and related transporters to develop therapies for epilepsy and other seizure disorders - potentially using Cmpd38J-derived probes.

## Methods

### Ethics statement

All animal experiments in this study were approved by the Institutional Animal Care and Use Committee (IACUC) of the Icahn School of Medicine at Mount Sinai. All animals were housed and maintained on a 12:12-hour light:dark cycle (with lights on at 07:00) in a temperature- (20 ± 2 °C) and humidity-controlled (30–70%) vivarium and free access to food and water.

For primary neuron culture, we used timed-pregnant C57BL/6 J female mice at embryonic day 16 (E16), which were purchased from Jackson Laboratory. The pregnant mice were sacrificed by cervical dislocation to harvest embryos. Sex was not considered in this study. For the brain slice experiments, we used male C57BL/6 J mice aged 35–42 days (P35-P42) purchased from Jackson Laboratory, and the mice were sacrificed by decapitation after isoflurane anesthesia.

### NBCn2 Compounds

All compounds tested for NBCn2 inhibitory activity were obtained from commercial sources as outlined in Supplementary Tables 3, 4.

### Construct design and protein expression

The cryoEM construct of human NBCn2 contains truncations of residues 2–90 and 231–299 in loop regions of the N-terminal domain. This construct was cloned into a pFastBac vector with a C-terminal 3C protease tag and 10 × His tag as previously done for SLC4A1/AE1^14^ (The construct is available from the corresponding author upon request). To obtain protein for cryoEM studies, the Bac-to-Bac baculovirus expression system was used. Briefly, bacmid DNA produced in DH10Bac cells was mixed with FuGENE HD transfection reagent and added to 0.5 × 10^6^ Sf9 cells (Expression Systems) plated in Sf900 II media to generate a virus whose titers were further determined by flow cytometry using gp64-PE antibody (94-001S, Expression Systems) at 1:400 dilution. Virus was then used to infect Sf9 cells in ESF 921 insect cell culture medium (96-001-01, Expression Systems) at a multiplicity of infection of 3 for 48 hours at 27 °C, before cells were spun down and stored at – 80 °C until use.

### Protein purification

Cells from 5 L of culture were homogenized in a dounce-homogenizer in low-salt buffer containing 10 mM HEPES (pH 7.5), 10 mM MgCl_2_, 20 mM KCl, and protease inhibitor cocktail (1 µM Leupeptin, 1 µM E-64, 150 nM Aprotinin and 500 µM AEBSF). After ultracentrifugation, the cell membrane pellet was washed twice with high-salt buffer containing 10 mM HEPES (pH 7.5), 10 mM MgCl_2_, 20 mM KCl, 1 M NaCl, and protease inhibitor cocktail. Purified membranes were resuspended in 10 mM HEPES (pH 7.5), 150 mM NaCl, 10 mM MgCl_2_, 20 mM KCl, and 2 mg/ml iodoacetamide at 4 °C and incubated for 30 min under agitation. Then, membranes were solubilized with buffer containing 10 mM HEPES (pH 7.5), 150 mM NaCl, 1% (w/v) n-dodecyl-β-D-maltopyranoside (DDM), and 0.2% (w/v) cholesteryl hemisuccinate (CHS) at 4 °C for 2 h under agitation. The sample was centrifuged at 200,000 × *g* for 35 min and the supernatant was incubated with TALON IMAC resin (Clontech) and 20 mM imidazole under agitation overnight.

To obtain purified NBCn2, the resin was washed consecutively with buffer 1 (25 mM HEPES (pH 7.5), 500 mM NaCl, 0.1% (w/v) DDM, 0.02% (w/v) CHS, and 20 mM imidazole), buffer 2 (25 mM HEPES (pH 7.5), 500 mM NaCl, 0.05% (w/v) DDM, 0.05% (w/v) lauryl maltose neopentyl glycol (LMNG), and 0.02% (w/v) CHS), buffer 3 (25 mM HEPES (pH 7.5), 500 mM NaCl, 0.05% (w/v) LMNG, and 0.01% (w/v) CHS), and buffer 4 (25 mM HEPES (pH 7.5), 500 mM NaCl, 0.01% (w/v) LMNG, and 0.002% (w/v) CHS), exchanging the detergent from DDM to LMNG. NBCn2 was then eluted from the resin in 25 mM HEPES (pH 7.5), 500 mM NaCl, 0.01% (w/v) LMNG, 0.002% (w/v) CHS, and 250 mM imidazole. PD miniTrap G-25 columns (GE Healthcare) were used to remove imidazole, and the protein was analyzed by SDS-PAGE. To obtain monodisperse NBCn2 for cryoEM studies, the apo sample was purified via size-exclusion chromatography (SEC) in 20 mM HEPES (pH 7.5), 150 mM NaCl, 0.0011% (w/v) LMNG, 0.00011% (w/v) CHS, and 0.00025% GDN. For NBCn2 bound to substrate or inhibitor, 100 mM NaHCO_3_ or 100 µM Cmpd38J, respectively, was added to the SEC buffer. Finally, peak fractions were collected, and samples were concentrated to 2–5 mg/ml for grid preparation.

### CryoEM sample preparation, data collection and processing

To prepare the grids for cryoEM data collection, holey carbon EM grids (Quantifoil 300 copper mesh, R1.2/1.3) were glow-discharged, and three microliters of the protein samples were loaded on the grids. The EM-GP2 plunge freezer (Leica) was set to 95% humidity and 10 degrees, and the samples were frozen in liquid ethane and stored in liquid nitrogen.

The data for the NBCn2-apo and NBCn2-Cmpd38J complexes were collected at the New York Structural Biology Center, and the data of the NBCn2-substrate complex was collected at the Laboratory for BioMolecular Structure at Brookhaven National Laboratory. All data was collected on Titan Krios instruments equipped with Falcon IV or Gatan K3 direct detectors. The microscopes were operated at 300 keV accelerating voltage, and the pixel size of apo-, substrate-, and Cmpd38J-bound NBCn2 data collection was 0.833 Å, 0.4125 Å, and 1.069 Å, respectively. At least 4000 movies were collected for each structure at a dose rate of 61.36 e^–^/Å^2^, 50.00 e^–^/Å^2^, and 52.48 e^–^/Å^2^, respectively.

All data were imported and processed in cryoSPARC^50^, and movies were subjected to motion correction using MotionCor2^51^ followed by CTF estimation. We initially used blob picking to select and extract initial particle stacks, as well as generate templates following 2D classification and ab initio jobs. Particles were then picked using template picking, followed by extraction and 2D classification before ab initio reconstruction. After multiple rounds of heterogeneous refinement using reconstructions from bad particles as decoys, we continuously eliminated defective particles to sequentially produce higher and higher resolution maps. Finally, non-uniform refinement^52^ was performed to obtain the final map. For the NBCn2-Cmpd38J complex structure, we performed 3D classification to discard additional particles, likely from unliganded NBCn2, to obtain a final map for unambiguous Cmpd38J placement. Finally, ChimeraX^53^, PHENIX^54^, Coot^55^, and PyMOL^56^ were used to build the models and analyze the maps.

### Cellular bicarbonate uptake assay

To perform cellular NBCn2 and NDCBE substrate transport assays, we adapted our protocols from work on AE1^14^ and generated monoclonal cell lines that stably express NBCn2, NBCn2 mutants, or NDCBE upon tetracycline induction using the Flp-In T-REx 293 system (R78007, ThermoFisher; all constructs are available from the corresponding author upon request). NBCn2- or NDCBE-mediated substrate transport was then determined by measuring cellular pH changes between induced and uninduced cells in response to CO_3_^2-^/HCO_3_^-^ import. We used the dye BCECF-AM (2’,7’-bis-(2-carboxyethyl)-5-(and-6)-carboxyfluorescein, acetoxymethyl ester), whose fluorescence ratio (excitation 495/20 nm and 435/20 nm; emission 540/30 nm) changes in response to different pH, which has been used by us and others to determine SLC4-mediated transport^10,11,14^.

For the assay, cells were plated in a 96-well clear-bottom plate (Greiner) with 75,000 cells per well and incubated overnight with or without 2 µg/ml tetracycline in 200 µl DMEM, 10% FBS, and penicillin/streptomycin, 10 µg/ml blasticidin S, and 100 µg/ml hygromycin B. The next day, the media was discarded, and cells were incubated in Hank’s Balanced Salt Solution (HBSS) supplemented with 25 mM Tris-Cl (pH 7.4), 5 µM BCECF-AM, and 30 µM amiloride for 30 mins. Then, cells were incubated with HBSS supplemented with 25 mM Tris-Cl (pH 7.4), indicated concentrations of (putative) NBCn2 inhibitors or vehicle (DMSO), and 30 µM amiloride for 30 mins. After incubation, the cells were washed twice with 100 µl/well Na^+^-free buffer (25 mM Tris pH 7.4, 115 mM TMA-Cl, 2.5 mM K_2_HPO_4_, 1 mM CaCl_2_, 1 mM MgCl_2_, 5 mM glucose, and 30 µM amiloride), and then incubated in 100 µl of the same buffer containing NBCn2 inhibitors at indicated concentrations or DMSO for 20 mins. Then, the cells were incubated in the same buffer including 25 mM TMA-HCO_3_ and inhibitors/DMSO for another 5 mins and a baseline pH was measured using a multimode plate reader (Victor NIVO, Perkin Elmer). After this measurement, the buffer was aspirated, and HBSS supplemented with 25 mM Tris-Cl (pH 7.4), 25 mM NaHCO_3_, 30 µM amiloride, and inhibitors/DMSO was added to the wells, and the pH was measured every minute for 15 min. This buffer exchange leads to a precipitous drop in cellular pH, which then recovers over time, facilitated by NBCn2 or NDCBE, and differences between transporter-expressing cells (induced) and control cells (uninduced) are plotted as pH differences observed during the measurement and normalized to the maximal ΔpH. We note that this assay was optimized for compound screening at medium throughput, requiring the indicated addition of 25 mM HCO_3_^-^ to obtain sufficient signal. Since no CO_2_ was added, the majority of HCO_3_^-^ likely dissipates as CO_2_ from the solutions, and the exact substrate concentration is therefore unknown, which precludes the determination of inhibitory potencies outside of relative IC50s.

### ELISA

To assess NBCn2 mutation expression, ELISA was performed as follows: cells were plated in a 96-well clear-bottom plate with 75,000 cells per well and incubated overnight. The next day, we removed the media and incubated cells with 100 µl of 4% paraformaldehyde at room temperature for 15 mins, then washed the cells with 100 µl PBS three times. Cells were incubated with 100 µl PBS containing 0.01% digitonin at room temperature for 15 mins, followed by incubating cells with 100 µl PBS containing 3% BSA at room temperature for 2 h. Then, the cells were incubated with human NBCn2 rabbit polyAb (Proteintech, Cat No. 27197-1-AP) at a 1:1000 dilution at 4 °C overnight. The next day, cells were washed with 100 µl of PBS three times and incubated with fluorescein 488-conjugated anti-rabbit IgG (Jackson ImmunoResearch, Cat No. 711-545-152) at room temperature for 2 h. After incubation, cells were washed with 200 µl PBS three times and then measured using a multimode plate reader (Victor NIVO, Perkin Elmer). After the measurement, cells were stained with 100 µl of 0.2% Janus green for 5 mins to normalize for cell count. Subsequently, cells were washed 5 times with MilliQ water. After washing, we added 200 µl of 0.5 M HCl to each well and incubated for 10 mins. Cells were read in the SpectraMax LUM at 595 nm wavelength.

### Virtual ligand screening

The herein-determined cryoEM structure of NBCn2 in the apo form was used for virtual screening with Glide^33–35^ in the Schrödinger suite (version 2022-1). To prepare the structure for docking, we used the Protein Preparation Wizard from the Schrödinger suite, where the structure was refined by optimization and minimization^57^. The structure was energy minimized until RMSD was 0.30 Å for heavy atoms with the OPLSe3 force field^58^. SiteMap was utilized to identify and define the substrate binding site for docking^59^. The receptor grids were then generated based on the substrate binding site that also showed the best SiteScores, using two pockets, one on Chain A (sitemap_NBCn2_v1_site_3) and the other one on Chain B (sitemap_NBCn2_v1_site_4). In the grid generation step, each of these selected sites was used as the grid site and the Receptor Grid Generation tool was utilized.

We virtually screened a library of 2.4 million lead-like compounds and a library of 800 K fragments from the ZINC15 database^60,61^ against the substrate-binding site of the apo NBCn2 structure. The previously prepared grid files were used as inputs for the Virtual Screening Workflow (VSW) tool of Glide, Maestro, which performs a three-step virtual screening process^33–35^. First, we performed a high-throughput virtual screen (HTVS) and selected the top 10% of compounds based on their scoring metrics. These selected compounds underwent additional virtual screening using standard-precision (SP) docking in Glide, where the 10% top scoring compounds were docked using Glide’s extra-precision (XP) docking^34^. Next, to assess the relative binding affinity of the compounds, we used molecular mechanics generalized Born surface area solvation (MM-GBSA) in Prime^62^, with top-ranking ligand-protein complexes (top 2000) identified in the virtual screening as input.

Finally, the top 200 compounds based on MM-GBSA from each virtual screen were subjected to visual inspection using PyMOL^56^ to discard likely false positive predictions. Erroneous docking often occurs in large screens, where typical errors include docking poses with high internal energies or unbound polar groups, as well as molecules with strained conformations^36,63^. 37 compounds were ultimately purchased and tested.

### Loop modeling

MODELLER^64,65^ (version 10.5) was used to model the missing extracellular loop of the substrate-bound NBCn2 structure used in the simulations. In particular, to generate multi-template homology models of Na^+^, Na^+^ and CO_3_^2-^-bound NBCn2, we used the AutoModel function in MODELLER based on NDCBE (PDB: 7RTM), an AlphaFold^28^-generated full-length NBCn2 model, as well as respective substrate-bound NBCn2 structures as templates. The ions were transferred from the substrate-NBCn2 structure into the generated models. The best scoring models using Z-DOPE^64,65^ (i.e., − 0.82772 for CO_3_^2-^ bound NBCn2) were used in the simulations.

### Site identification by ligand competitive saturation (SILCS) simulations

SILCS^25^ (SilcsBio LLC, version 2024.1) and GROMACS^66^ (version 2020.3) were used to map the potential cation and anion selective binding sites in the Na^+^, Na^+^ and CO_3_^2-^-bound NBCn2 monomer after modeling missing extracellular loop residues using MODELLER^64,65^ as described above without including the ions in the model. Model refinement was performed using Protein Preparation Wizard^57^ in the Schrödinger Suite. Following the standard membrane protein system set up protocol in SILCS, the substrate-bound NBCn2 structure without any ion or water molecules was embedded in a 9:1 POPC: cholesterol bilayer cell membrane system. The CHARMM36 force field^67^ was used for the protein, lipids, and ions, and the CHARMM General Force Field^68^ was used for the fragment molecules, as described previously^10,25^. The TIP3P water model was used in the simulation^69^. Probes used in the simulation were benzene, propane, dimethyl ether, methanol, formamide, imidazole, acetate, methylamine and water molecules. Ten discrete systems with different initial velocities were prepared after random distribution of probes. Before GCMC/MD simulations, energy minimization and 6-step pre-equilibration were performed to slowly relax the bilayer system at 298 K and 1 bar. A weak harmonic positional restraint was applied to all Cα atoms during the entire SILCS simulations with a force constant of 0.1 kcal/mol/Å^2^. The initial GCMC simulations were done to redistribute probes. The GCMC/MD simulation for each system was run for 100 cycles, with the combined MD simulation time of 1 μs for ten systems (100 ns × 10). Once the simulation was completed, occupancy maps (FragMaps) of ten individual simulation systems were generated using a default grid spacing of 1 Å. FragMaps from individual simulations were combined and converted into GFE (grid-free energy maps) FragMaps. Each voxel in the GFE FragMaps contains GFE values for a specific probe type. FragMaps generated from acetate, carboxylate carbon and methylamine nitrogen were shown to represent the anion and cation selective binding sites, respectively, with GFE energy thresholds as − 0.7 kcal/mol. Other FragMaps, such as generic hydrogen bond donor and acceptor, were also generated but are not discussed here.

### MD simulations

Although the NBCn2 structure was solved as a dimer, it was suggested that dimerization is not essential for transport through SLC4 members and transport occurs in each monomer independently^10,26^ and, accordingly, only one monomer was used in the simulations. MD simulations were performed with GROMACS^66^ (v2020.3), using the CHARMM36m^70^ all-atom force field and the TIP3P water model^69^. The input structures for the MD simulations were structures determined in this study: Cmpd38J-bound NBCn2 and substrate-bound NBCn2 structures with the extracellular loop modeled using MODELLER as described above. The input structures were embedded in a POPC lipid bilayer in a periodic box with 22.5 Å layers of water on both sides of the membrane and 150 mM NaCl (Supplementary Table 2). The CHARMM General Force Field^68^ was used for Cmpd38J. The bilayer membrane system underwent a systematic minimization and six-step equilibration process following the standard CHARMM-GUI^71,72^ protocol for GROMACS, which includes sequential simulations in the NVT and NPT ensembles, with position restraints on protein, ligand, and lipid atoms gradually switched off. Four independent replicate MD simulations using the same initial coordinates with different initial velocities were performed at 303.15 K using the v-rescale method and 1 bar using the Parrinello-Rahman method with semi-isotropic coupling for a combined time of 1 μs for Cmpd38J-bound NBCn2, and 1.2 μs for substrate-bound NBCn2. Particle Mesh Ewald was used for the long-range electrostatic interactions, and the non-bonded interactions were cut-off and switched off at 12 and 10 Å, respectively. MDAnalysis^73^ (v 2.2.0) and ProLIF^74^ (v 2.0.1) were used for trajectory analysis and protein-ligand interactions, respectively. Clustering analysis of Cmpd38J was carried out by concatenating trajectories of four replicas, and then we calculated RMSD on all heavy atoms of Cmpd38J and residues of NBCn2 within 5 Å of Cmpd38J from the concatenated trajectory. The gromos algorithm^75^ with a cutoff as 1.2 Å was used. Clusters which occupy less than 0.5% of total conformations were not included in the visual inspection.

### Molecular docking and hit optimization

Potential analogs of the hit Cmpd38 were identified through a chemical similarity search of the ZINC20 database by setting a Tanimoto Coefficient (Tc) cutoff as 0.4. Tanimoto values were calculated using Morgan fingerprints and the Tanimoto similarity function in RDKit (v2022.03.5)^76^. All similarity values are reported as decimals. To characterize the binding modes of Cmpd38 and its analogs using molecular docking, we first removed the ligand from the cryoEM structure of the NBCn2-Cmpd38J complex structure. NBCn2 was then prepared with the Maestro Protein Preparation Wizard of the Schrödinger suite (v2022-1) under default parameters^57^. The binding site was defined by generating a grid with the Receptor Grid Generation Panel. The binding site outlining box was defined by supplying ligand centroid coordinates of Cmpd38J in the complex structure. LigPrep was performed under the default parameters using SMILES of the Cmpd38 analogs as input. Docking was performed using Glide^33–35^ by setting 5 poses per ligand, written out as output. Finally, we used MM-GBSA with Prime in the Schrödinger suite to estimate the relative binding affinity between ligand and protein, where a more negative value of ΔG binding indicates higher binding affinity^62^. The correlation between the best MM-GBSA per ligand and its experimental bicarbonate inhibition effect was calculated and visualized using the ggstatsplot R package (v0.11.1)^77^.

### Pocket volume analysis

PyVOL (v1.7.8)^78^ was used to calculate substrate binding site volumes using the apo-NBCn2, substrate-bound NBCn2 and Cmpd38J-bound NBCn2 structures as input, with minimum and maximum probe radii set as 1.4 Å and 3.4 Å, respectively.

### Ion modeling using AlphaFold3

AlphaFold3 (AF3)^28^ predictions were performed using the publicly released DeepMind AlphaFold3 package (v3.0.1; https://github.com/google-deepmind/alphafold3). The Docker image was converted into a Singularity image and executed on the Minerva HPC cluster. AF3 was used to predict the structure of the NBCn2 monomer (Uniprot ID: Q6U841) using two input conditions: (1) NBCn2 with two sodium ions, one carbonate ion, and three water molecules specified as ligands based on the cryo-EM substrate-bound structure, and (2) NBCn2 with two sodium ions only. A total of 1100 models were generated using 220 random seeds, with five models produced per seed. Predicted structures were aligned to the observed substrate-bound NBCn2 structure, and RMSD values were calculated. Models with RMSD values exceeding 3 Å relative to the reference structure were excluded from further analysis. Molecular interactions and coordination of Na^+^(1), Na^+^(2), and the carbonate ion were characterized using PLIP (v3.0.0) analysis^29^ and interatomic distance measurements.

### Preparation and treatment of primary murine brain neuron-glia co-cultures

Primary murine brain neuron-glia co-cultures were prepared and analyzed following the general guidelines described by Napoli and Obeid^38^. In particular, brain specimens were isolated from a total of nine E16 C57BL/6 J mouse embryos. Following the removal of meninges, freshly isolated embryonic brain tissue was mechanically triturated in a serum-containing cell preparation media (ATCC Dulbecco’s Modified Eagle’s Medium containing 10% fetal bovine serum and 1% penicillin-streptomycin). For qPCR studies, dissociated cells in the serum-containing cell preparation media were seeded onto 24-well BioCoat Poly-D Lysine-Treated flat-bottom microplates (Corning) at a density of 500,000 cells per well. After 24 h, the cultured media was completely switched to a serum-free, chemically defined, B-27-supplemented Neurobasal-Plus culture media (Gibco B-27 Plus Neurobasal Culture System containing 0.5 mM Gibco GLutaMAX-I supplement and 1% penicillin-streptomycin). Cells were re-fed every 3-4 days by exchanging 50% of the media in the wells with the fresh serum-free culture media.

### Spontaneous neuronal activity assessment

For spontaneous neuronal activity studies, cells were plated onto multi-electrode array (MEA) plates (Axion Biosystems). In particular, MEA plates were first coated with a commercial Poly-D-Lysine solution following the manufacturer’s instructions and were then seeded with freshly isolated brain cells at a density of 60,000 cells per well. Freshly seeded neuron-glia co-cultures in MEA plates were maintained for 24 h in the serum-containing cell preparation media. The culture media was then completely switched to the serum-free, B-27-supplemented Neurobasal-Plus culture media system. Cells were refed every 3-4 days by exchanging 50% of the media in the wells with the fresh serum-free culture media. Spontaneous neuronal activity was then assessed using an Axion Maestro MEA reader (Axion Biosystems). All neuronal activities were monitored and recorded for 10 minutes using the AxiS 2.4 software; quantitative analysis of the recording was also conducted using the AxisS2.4 software.

### NBCn2 Knockdown via antisense oligos

NBCn2 AUMsilenceTM ASO (NBCn2 antisense oligo sequence: TTAGTATTCGGATCAGGTAGC) and AUMscrTM oligo (scrambled control oligo sequence: GCCGTTTACGTCGTCACAGA) (AUM BioTech) were diluted into the culture media and directly layered over the cultured cells at a final concentration of 1 µM on DIV14 and DIV17. Cmpd38J was solubilized in DMSO. The Cmpd38J solution and vehicle (DMSO) solution were then diluted into the cultured media and layered over the cultured cells at a final concentration of 100 µM.

### Quantitative real-time polymerase chain reaction (qPCR) studies

qPCR studies were essentially conducted as described^79^. Primary cells in 24-well plates were washed with ice-cold PBS, and total RNA was prepared from individual wells using the RNeasy Mini Kit (Qiagen), following the manufacturer’s instructions. Contents of mNBCn2, mNBCn1, mNDCBE, and mGAPDH RNAs were assessed by qPCR using mNBCn2 (FOR: GAAAGAGGGACAGAGAGAGAGA, REV: CATCGTCCTCAGTTCCAAGAATA), mNBCn1 (FOR: CCAAGTTTCTGGGAATTCGTGAACAG, REV: CTGGCATGAGGTCATCAAGCCAAC)^80^, mNDCBE (FOR: ATCGGCACCACCGCACTCAT, REV: AGCAGGGCTTCCCGCACTTT)^81^, and mGAPDH (FOR: AGGTCGGTGTGAACGGATTTG, REV: TGTAGACCATGTAGTTGAGGTCA) qPCR primer sets. Data were normalized relative to those for mGAPDH RNA. Relative mNBCn2, mNBCn1, and mNDCBE RNA contents in the experimental group were expressed relative to those in the control group using the 2^-ΔΔCt^ method^82^.

### Cell cytotoxicity assay

Cell cytotoxicity in neurons was measured using the Cell Counting Kit-8 (CCK-8; Dojindo, CK04-05) and the CytoTox 96 Non-Radioactive Cytotoxicity Assay (LDH; Promega, G1780). For the CCK-8 assay, following experimental treatments (NBCn2 knockdown or spike number quantification), the CCK-8 reagent was diluted in fresh neuronal culture medium, and the culture medium was completely replaced with CCK-8–containing medium. Cells were incubated for 1 h at 37 °C. Subsequently, 100 µL of supernatant from each well was transferred to a new 96-well plate, and absorbance was measured at 450 nm using a microplate reader. LDH (lactate dehydrogenase) release was measured according to the manufacturer’s instructions. Briefly, after the experiment treatment, 50 µl of culture medium from each well was transferred to a new plate and mixed with 50 µl of CytoTox 96 reagent. Samples were incubated for 30 mins at room temperature in the dark, after which 50 µl of stop solution was added. Absorbance was measured at 490 nm using a microplate reader.

### Determination of mIPSCs in brain slices

Electrophysiological recordings from CA1 neurons in acute hippocampal slices were performed as previously described^83–85^. In brief, horizontal brain slices (300 µm thick) were prepared from three male C57BL/6 J mice (P35-42) using a high-sucrose cutting solution containing: 85 mM NaCl, 75 mM sucrose, 2.5 mM KCl, 1.3 mM NaH_2_PO_4_, 24 mM NaHCO_3_, 0.5 mM CaCl_2_, 4 mM MgCl_2_, and 25 mM D-glucose. Slices were equilibrated in aCSF: 120 mM NaCl, 2.5 mM KCl, 1 mM NaH_2_PO_4_, 26.2 mM NaHCO_3_, 2.5 mM CaCl_2_, 1.3 mM MgSO_4_, and 11 mM D-glucose (290 mOsm) at 31.5 °C for 30 min, followed by 1 h at room temperature. For recordings, slices were transferred to a chamber containing aCSF supplemented with 0.5 µM tetrodotoxin, 10 µM CNQX, and 50 µM D-APV. In experiments involving Cmpd38J treatment, slices were incubated with 80 µM Cmpd38J for 1 h prior to recordings. Miniature inhibitory postsynaptic currents (mIPSCs) were recorded using an internal solution containing: 140 mM CsCl, 0.5 mM EGTA, 4 mM Mg-ATP, 0.3 mM Na_2_-GTP, 10 mM HEPES, 5 mM Na_2_-phosphocreatine, and 2 mM QX-314 (pH 7.3, adjusted with CsOH, ~ 300 mOsm). Cells were voltage-clamped at a holding potential of − 70 mV using a Multiclamp 700B amplifier (Molecular Devices), and data were acquired at 4 kHz with a 1 kHz low-pass filter using Clampfit 10 (Molecular Devices). Miniature events were manually selected starting at the third minute of recording and analyzed over a 1 min window using template matching in Clampfit 10 with a 5 pA detection threshold. We patched a total of 39 cells, and recorded from 20 cells in the presence of vehicle, and from 19 cells in the presence of Cmpd38J. Average frequencies and amplitudes over 1 min of recording are shown in Fig. 7C, D, respectively. We also analyzed the first 50 events from each cell during the 1 min recording, and calculated cumulative probability distributions that are shown in Fig. 7E, F.

### Statistical analysis

All values are expressed as mean ± s.e.m. Differences between means were analyzed using either *t* test, one-way ANOVA, or two-way ANOVA, where indicated. In all analyses, the null hypothesis was rejected at the 0.05 level. All statistical analyses were performed using GraphPad Prism (GraphPad Software, San Diego, CA, USA).

### Reporting summary

Further information on research design is available in the Nature Portfolio Reporting Summary linked to this article.

## Supplementary information

Supplementary Information

Reporting Summary

Transparent Peer Review file

## Source data

Source Data File

## Data Availability

Density maps and structure coordinates have been deposited in the Electron Microscopy Data Bank (EMDB) and the PDB. A structure of NBCn2-Apo has been deposited under EMD-48304 and PDB-9MIX, a structure of NBCn2-NaHCO_3_ has been deposited under EMD-48318 and PDB-9MJO, and a structure of NBCn2-Cmpd38J has been deposited under EMD-48320 and PDB-9MK6. Our study further includes a comparison with previous structures PDB-7RTM and PDB-7TY7. Source data are provided with this paper. Loop modeling, SILCS fragment map and MD simulation data are available on Zenodo (10.5281/zenodo.14983143). AlphaFold3 modeling data are available on Zenodo (10.5281/zenodo.20278709). Source data are provided in this paper.

## Code availability

Analysis scripts for MD simulations and AlphaFold3 modeling are available on GitHub (https://github.com/knk9596/SLC4A10_Modeling) and Zenodo (10.5281/zenodo.20278709).
