# Supplementary material for: Structural insights enable drug discovery for the neuronal NBCn2 carbonate transporter
Source: Nat Commun. 2026 Jul 22;17:8923. doi: 10.1038/s41467-026-75444-4 (PMC13503941; doi:10.1038/s41467-026-75444-4)
Supplement: Supplementary file 1 [file 41467_2026_75444_MOESM1_ESM.pdf]

Supplementary Information for

Structural insights enable drug discovery for the neuronal NBCn2 carbonate transporter

Shifan Yang[#], Yihan Zhao[#], Sezen Vatansever, Gregory Zilberg, Michael J. Capper, Jinglong Zhang, Joshua Stamos, Keino Hutchinson, Audrey L. Warren, Alexander C. Stone, Anwar Abbassi, Eric Purisic, Lap Ho, Aiqun Li, Jinye Dai, Avner Schlessinger*, Bin Zhang*, and Daniel Wacker*

*To whom correspondence should be addressed: Avner Schlessinger (avner.schlessinger@mssm.edu), Bin Zhang (bin.zhang@mssm.edu), Daniel Wacker (daniel.wacker@mssm.edu)

This file includes:

Supplementary Figures 1-13

Supplementary Tables 1-4

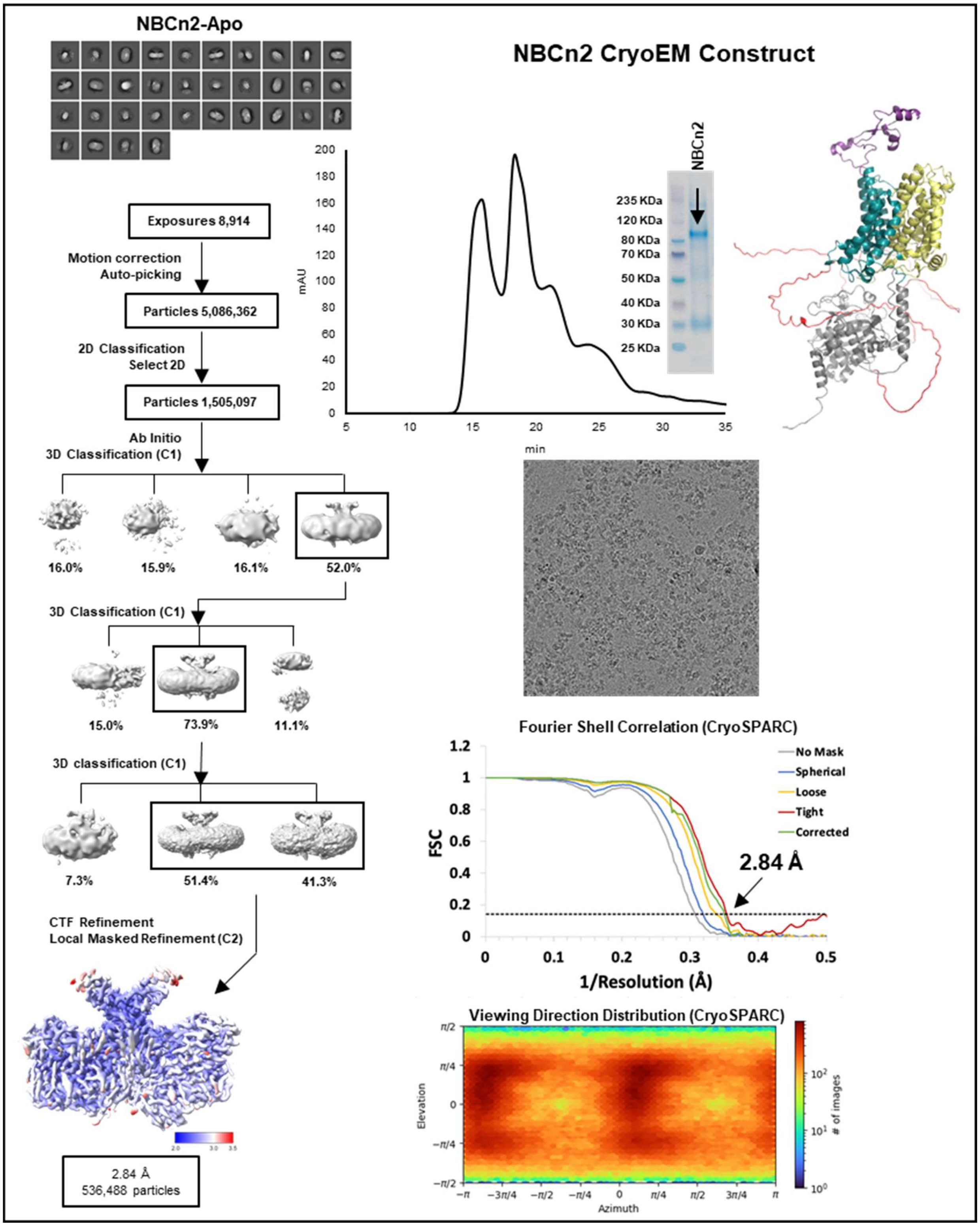
NBCn2-Apo
NBCn2 CryoEM Construct
Exposures 8,914
Motion correction
Auto-picking
Particles 5,086,362
2D Classification
Select 2D
Particles 1,505,097
Ab Initio
3D Classification (C1)
16.0%
15.9%
16.1%
52.0%
3D Classification (C1)
15.0%
73.9%
11.1%
3D classification (C1)
7.3%
51.4%
41.3%
CTF Refinement
Local Masked Refinement (C2)
2.84 Å
536,488 particles
mAU
min
NBCn2
235 KDa
120 KDa
80 KDa
70 KDa
50 KDa
40 KDa
30 KDa
25 KDa
Fourier Shell Correlation (CryoSPARC)
No Mask
Spherical
Loose
Tight
Corrected
FSC
2.84 Å
1/Resolution (Å)
Viewing Direction Distribution (CryoSPARC)
Elevation
Azimuth
# of images

Supplementary Fig. 1 | CryoEM structure determination of apo-NBCn2. Analytical size exclusion chromatography and SDS-PAGE show monodisperse and pure protein of apo-NBCn2. CryoEM construct is shown as AF3 model, with truncated regions shown in red, disordered regions of the N-terminal domain and C-terminus shown in gray, and ordered regions shown in purple, teal, and yellow. Data were collected on 300 keV Krios, a representative micrograph is shown, and processed in cryoSPARC: particles were picked from motion corrected micrographs, subjected to 2D classification (representative classes are shown), followed by ab initio model building and 3D classification. After multiple rounds of 3D classification, the final particle stack was subjected to local CTF refinement followed by local refinement of the masked NBCn2 membrane domain with imposed C2 symmetry. Final map was obtained with GS-FSC indicating a global resolution of 2.84 Å applying the 0.143 cutoff. The model was built and refined in PHENIX. Calculations in cryoSPARC indicate local resolutions of up to 2.5 Å around substrate binding site. Viewing direction analysis (cryoSPARC) indicates sufficient coverage and isotropic distribution of views in final particle stack. Source data are provided as a Source Data file.

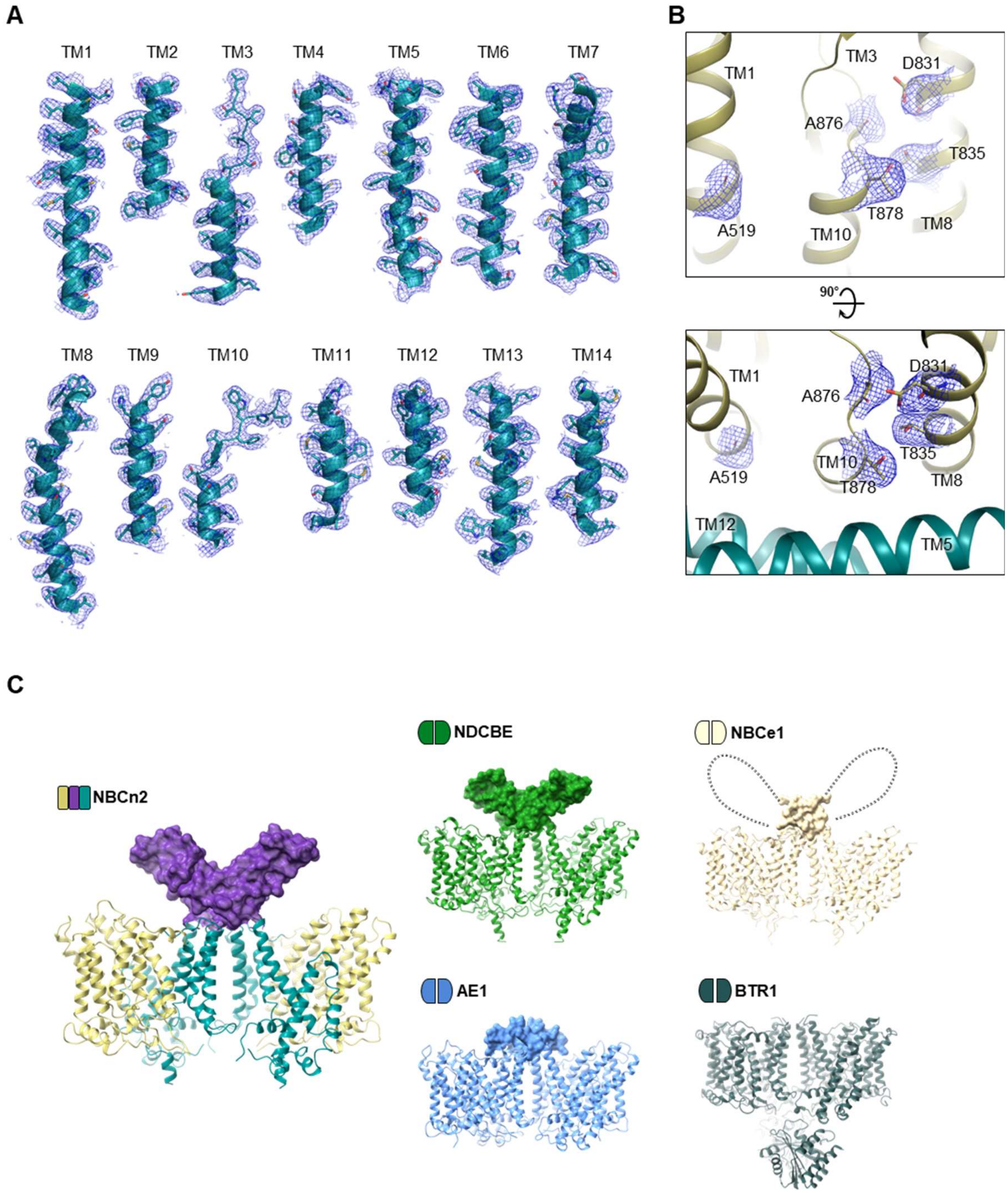

Supplementary Fig. 2 | Representative cryoEM density of apo-NBCn2 and comparison of different SLC4 transporter architectures. A, CryoEM densities (marine mesh) for apo-NBCn2 transmembrane helices (teal) using a contour level of 3σ. **B**, CryoEM densities (marine mesh) for residues in the substrate binding site of apo-NBCn2 using a contour level of 6σ. **C**, Comparison of NBCn2 with other SLC4 structures highlights structural differences in the overall architecture.

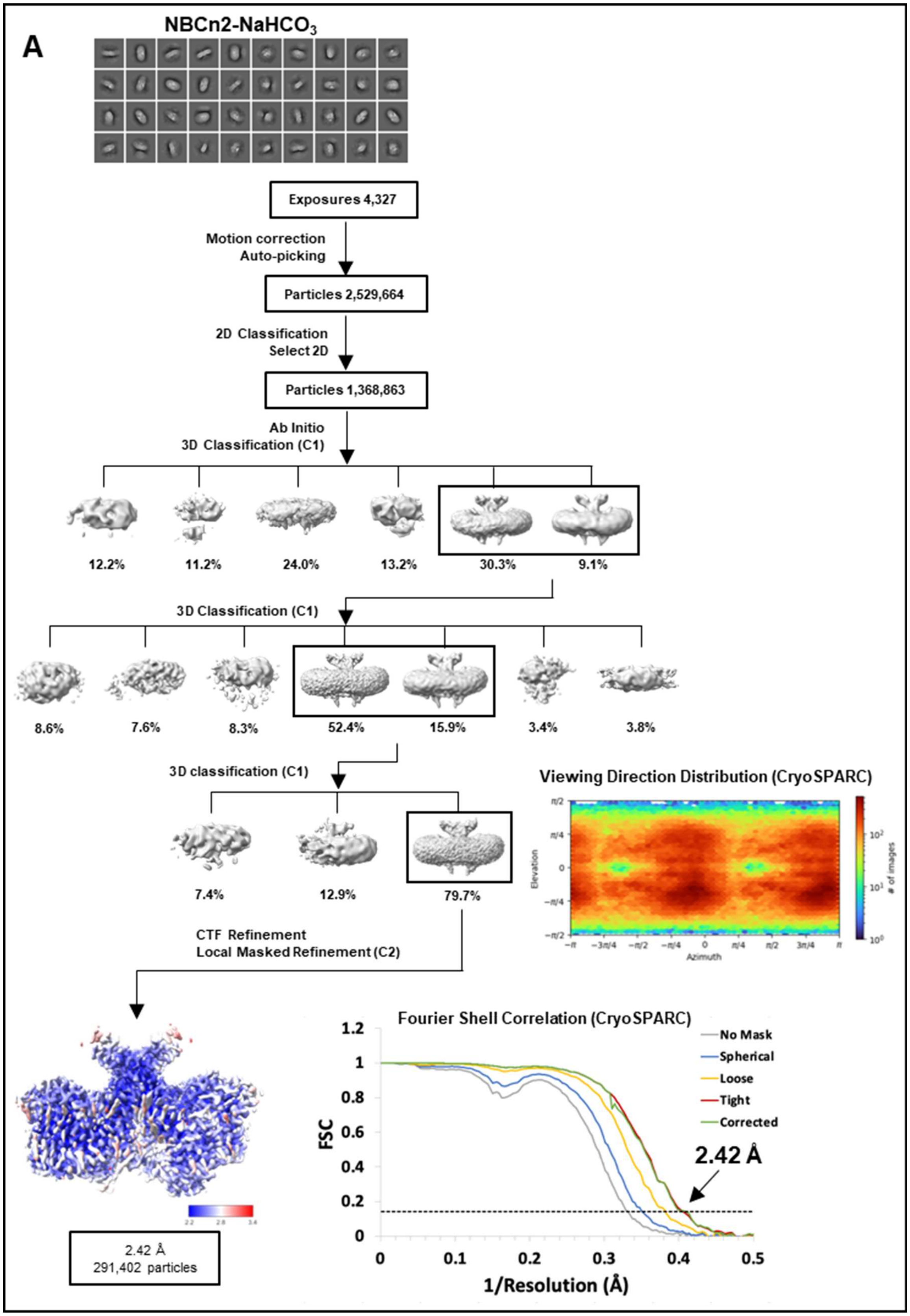
A
NBCn2-NaHCO$_3$
Exposures 4,327
Motion correction
Auto-picking
Particles 2,529,664
2D Classification
Select 2D
Particles 1,368,863
Ab Initio
3D Classification (C1)
12.2%
11.2%
24.0%
13.2%
30.3%
9.1%
3D Classification (C1)
8.6%
7.6%
8.3%
52.4%
15.9%
3.4%
3.8%
3D classification (C1)
7.4%
12.9%
79.7%
Viewing Direction Distribution (CryoSPARC)
Elevation
Azimuth
# of images
CTF Refinement
Local Masked Refinement (C2)
2.42 Å
291,402 particles
Fourier Shell Correlation (CryoSPARC)
No Mask
Spherical
Loose
Tight
Corrected
2.42 Å
FSC
1/Resolution (Å)

Supplementary Fig. 3 | CryoEM structure determination of substrate-bound NBCn2. Data were collected on 300 keV Krios and processed in cryoSPARC: particles were picked from motion corrected micrographs, subjected to 2D classification (representative classes are shown), followed by ab initio model building and 3D classification. After multiple rounds of 3D classification, the final particle stack was subjected to local CTF refinement followed by local refinement of the masked NBCn2 membrane domain with imposed C2 symmetry. Final map was obtained with GS-FSC indicating a global resolution of 2.42 Å applying the 0.143 cutoff. The model was built and refined in PHENIX. Calculations in cryoSPARC indicate local resolutions of up to 2.2 Å around substrate binding site. Viewing direction analysis (cryoSPARC) indicates sufficient coverage and isotropic distribution of views in final particle stack. Source data are provided as a Source Data file.

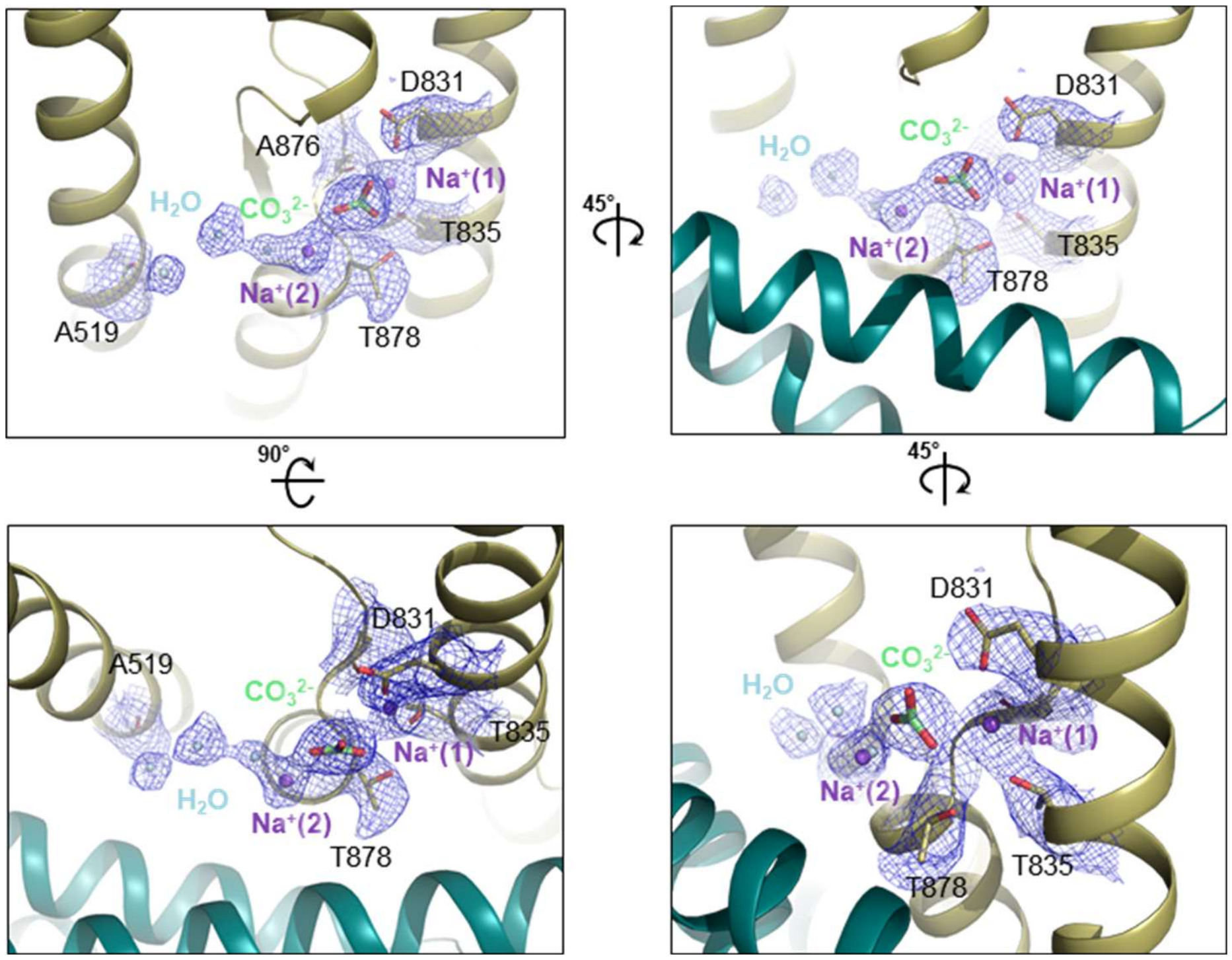

Supplementary Fig. 4 | Representative cryoEM density of substrate-bound NBCn2. CryoEM densities (marine mesh) for residues, ions, and water molecules in the NBCn2 substrate binding site using a contour level of 6σ.

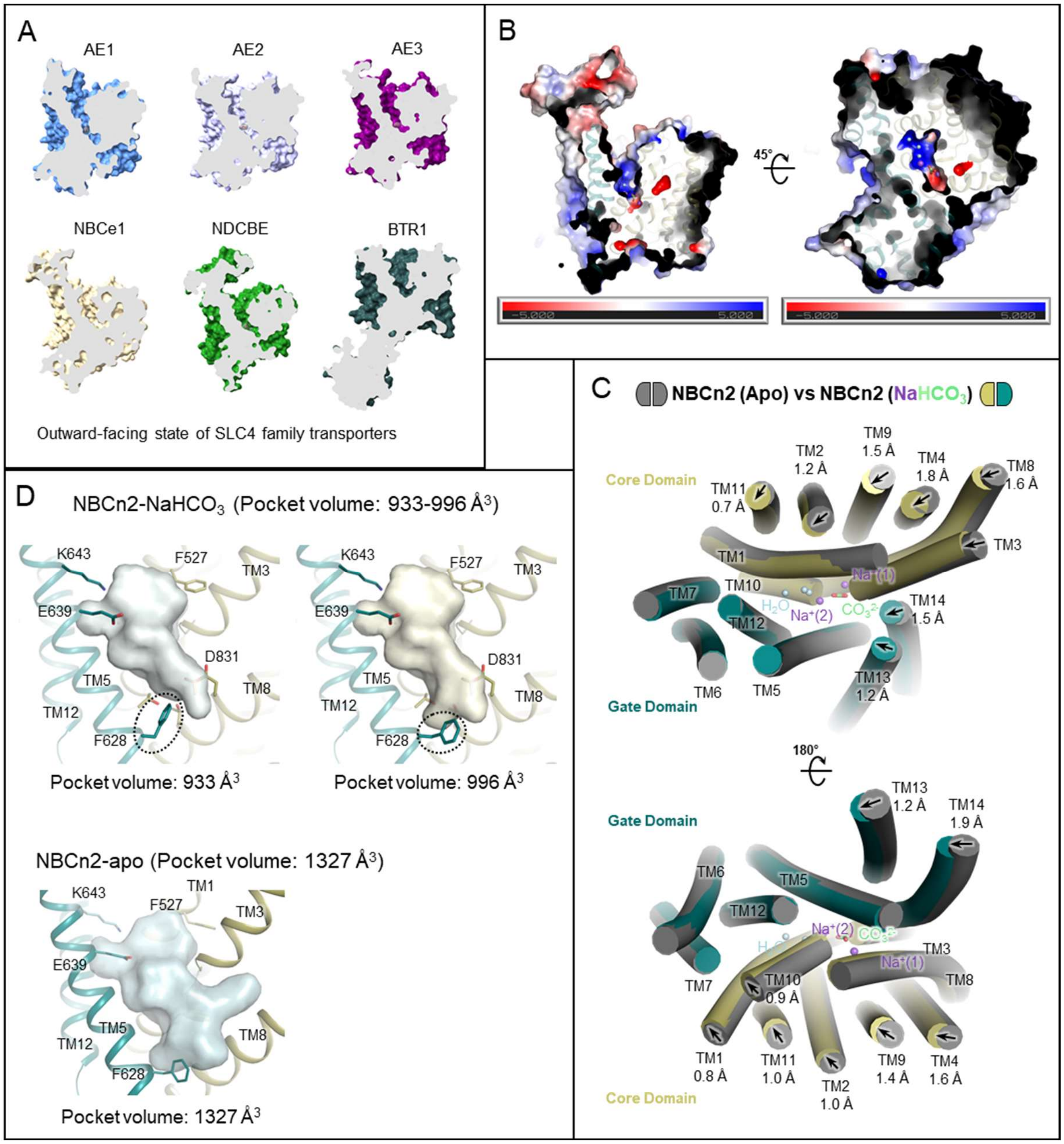

Supplementary Fig. 5 | Analysis of NBCn2's substrate binding pocket and comparison to other SLC4 transporters. A, Sliced side view of different SLC4 structures showing similar substrate entry channels that highlight the outward facing state of the transporters. **B**, Sliced side views of NBCn2 from the membrane (left) and extracellular side (right) colored by charge distribution highlighting positively (blue) and negatively (red) charged surfaces. **C**, Global structural changes between apo- (gray) and substrate-bound NBCn2 (colored by domain). **D**, Calculation of pocket volume in apo and substrate-bound NBCn2. F628 shows distinct rotamer states in the substrate-bound state that result in distinct pocket volumes.

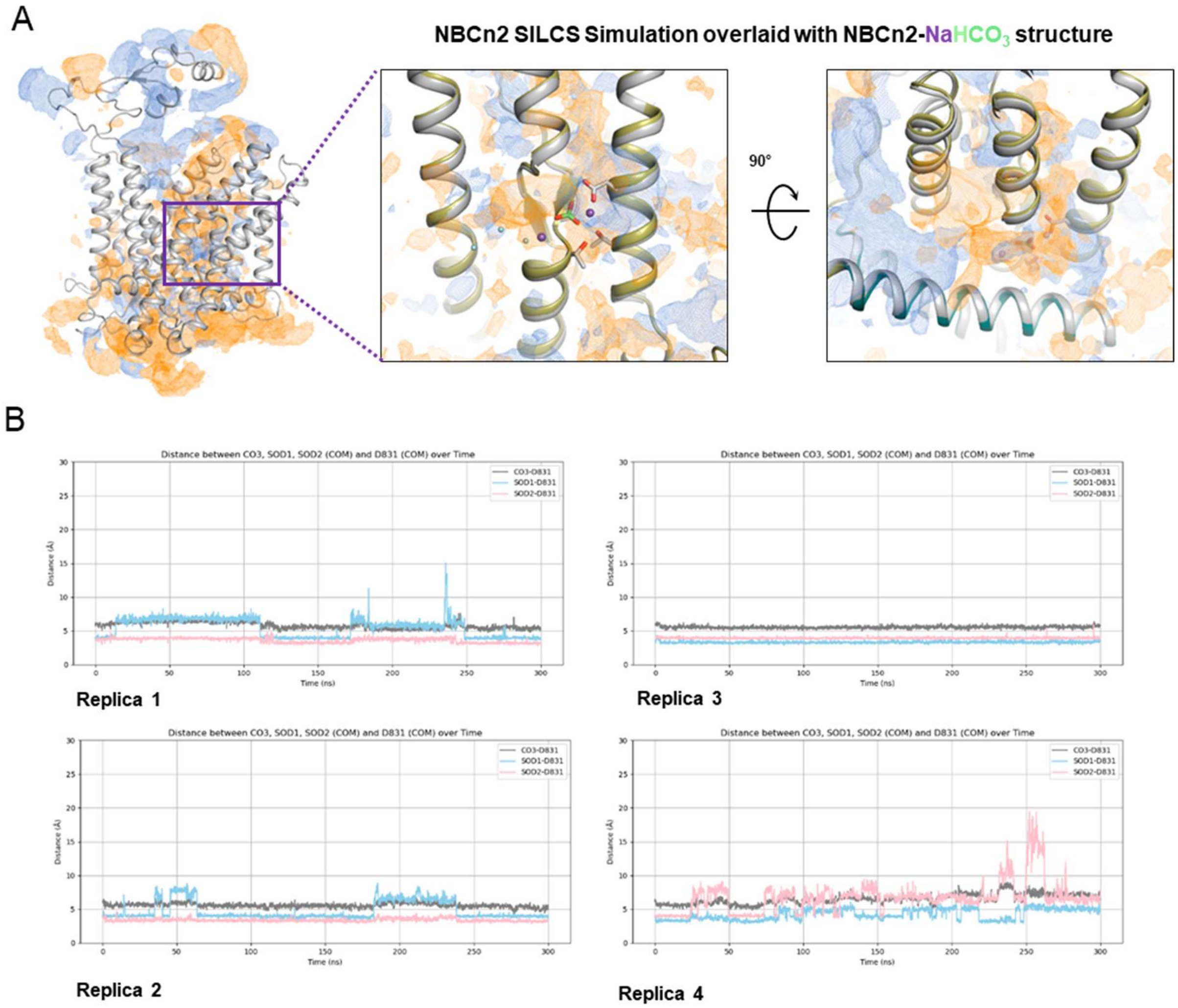

Supplementary Fig. 6 | Computational modeling and MD simulations of substrate-bound NBCn2. A, SILCS Fragment maps of NBCn2 (GFE=-0.7 kcal/mol) demonstrate the potential cation and anion binding sites, visualized by orange and blue mesh, respectively. Substrate-bound NBCn2 structure and SILCS Fragment maps of NBCn2 were aligned. **B**, Distances between Na^+(1), Na^+(2) and CO_3^{2-} and the NBCn2 substrate binding pocket over the course of the MD simulations shown for all 4 replicas. Na^+(1) is colored blue, Na^+(2) is colored pink and CO_3^{2-} is colored gray. Source data are provided as a Source Data file.

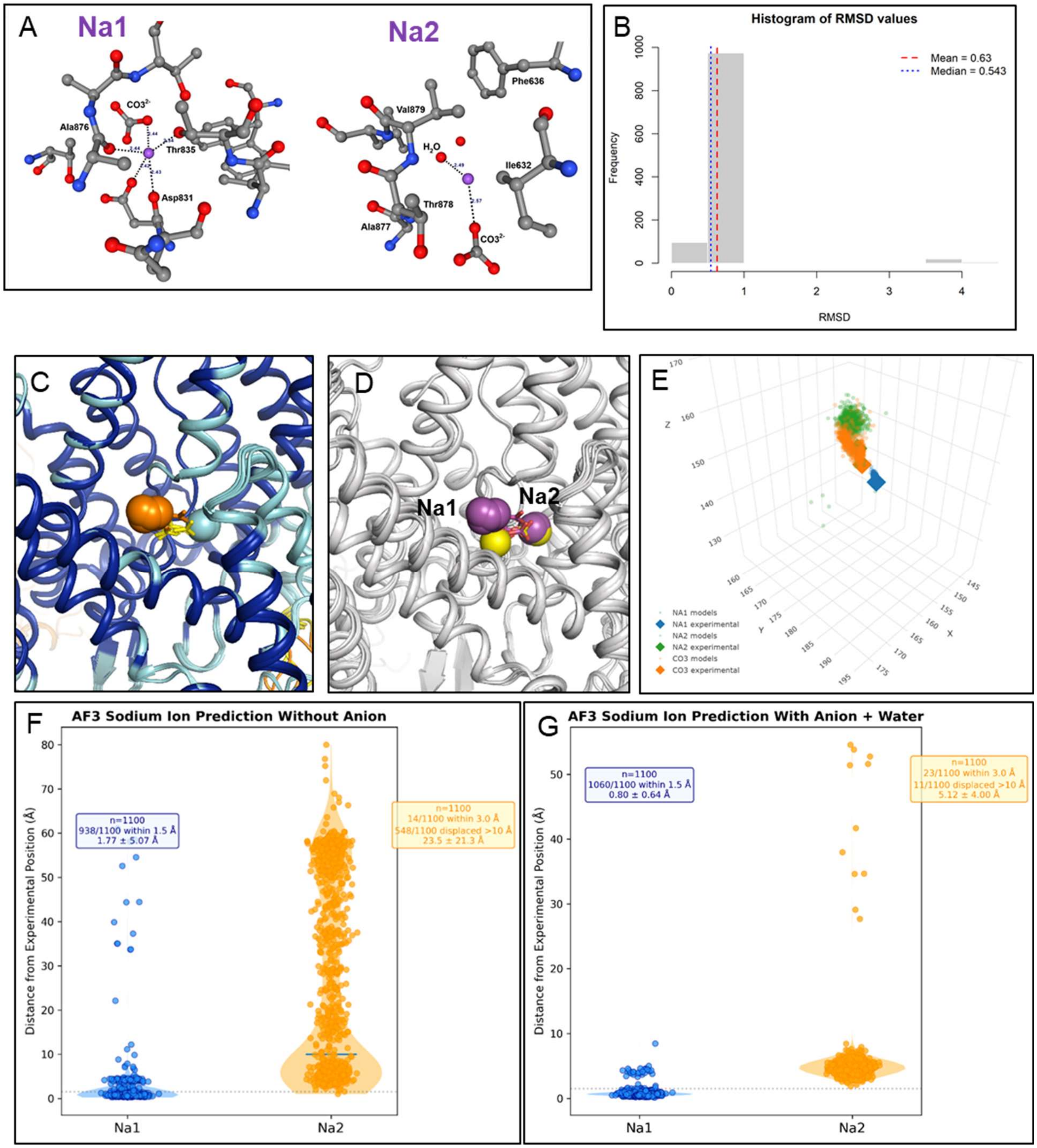

A
Na1
Na2
CO3²⁻
Ala876
Thr835
Asp831
Val879
Phe636
H2O
Ile632
Thr878
Ala877
CO3²⁻
B
Histogram of RMSD values
Mean = 0.63
Median = 0.543
Frequency
RMSD
C
D
Na1
Na2
E
NA1 models
NA1 experimental
NA2 models
NA2 experimental
CO3 models
CO3 experimental
F
AF3 Sodium Ion Prediction Without Anion
Distance from Experimental Position (Å)
n=1100
938/1100 within 1.5 Å
1.77 ± 5.07 Å
n=1100
14/1100 within 3.0 Å
548/1100 displaced >10 Å
23.5 ± 21.3 Å
Na1
Na2
G
AF3 Sodium Ion Prediction With Anion + Water
Distance from Experimental Position (Å)
n=1100
1060/1100 within 1.5 Å
0.80 ± 0.64 Å
n=1100
23/1100 within 3.0 Å
11/1100 displaced >10 Å
5.12 ± 4.00 Å
Na1
Na2

Supplementary Fig. 7 | Ion coordination modeling by AF3. A, Observed sodium-oxygen interactions of Na^+(1) and Na^+(2) according to CheckMyMetal server analysis using substrate-bound NBCn2 structure. **B**, RMSD distribution of 1,100 models relative to the cryo-EM structure. **C**, pLDDT values of five top-ranking AF3 models. Models are shown using AF3's default color scheme. **D**, Alignment between the top five AF3 models sorted by ranking scores and cryo-EM structure. Experimental derived coordinates of ions are shown in yellow, and predicted coordinates of ions are shown in purple. **E**, Three-dimensional coordinate distribution of ions from the cryo-EM structure and AF3 models. Experimentally derived coordinates are represented as diamonds, and AF3-predicted coordinates are shown as dots. **F**-**G**, AF3 modeling predicting experimental positions of Na^+(1) and Na^+(2) in the absence (**F**) and presence (**G**) of the anion and water molecules. Source data are provided as a Source Data file.

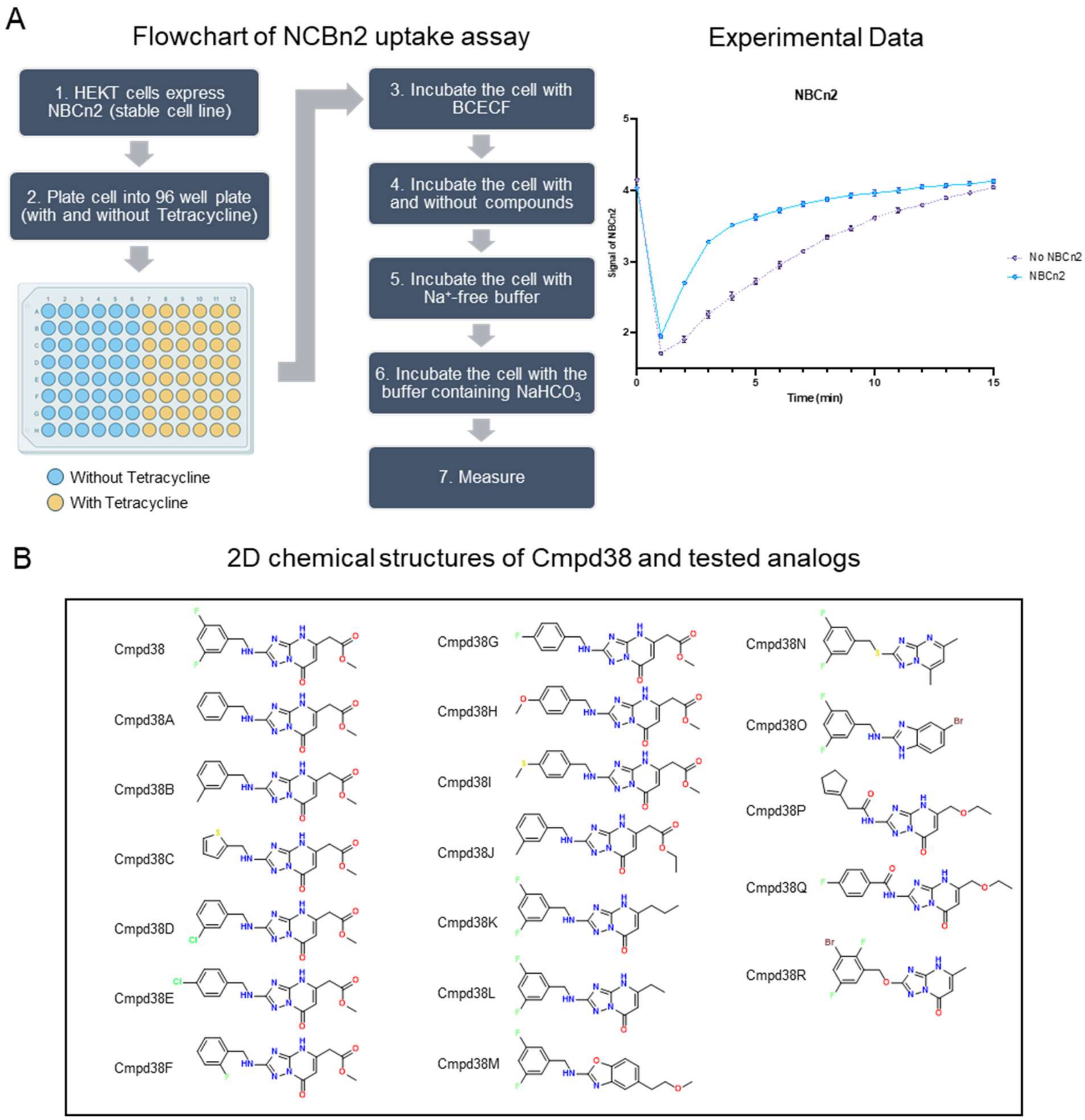

Supplementary Fig. 8 | Determining NBCn2-mediated substrate transport in HEK cells and analogs of NBCn2 inhibitor scaffold identified by virtual ligand screening. **A**, Flowchart of NBCn2 uptake assay (left) and example data (right) showing NBCn2-mediated recovery from acid load for cells expressing NBCn2 (blue) compared to uninduced cells (purple). Representative sample data (n=1) denotes mean± s.e.m. from 4 technical repeats. **B**, 2D chemical structures of Cmpd38 and analogs tested in this manuscript. Source data are provided as a Source Data file.

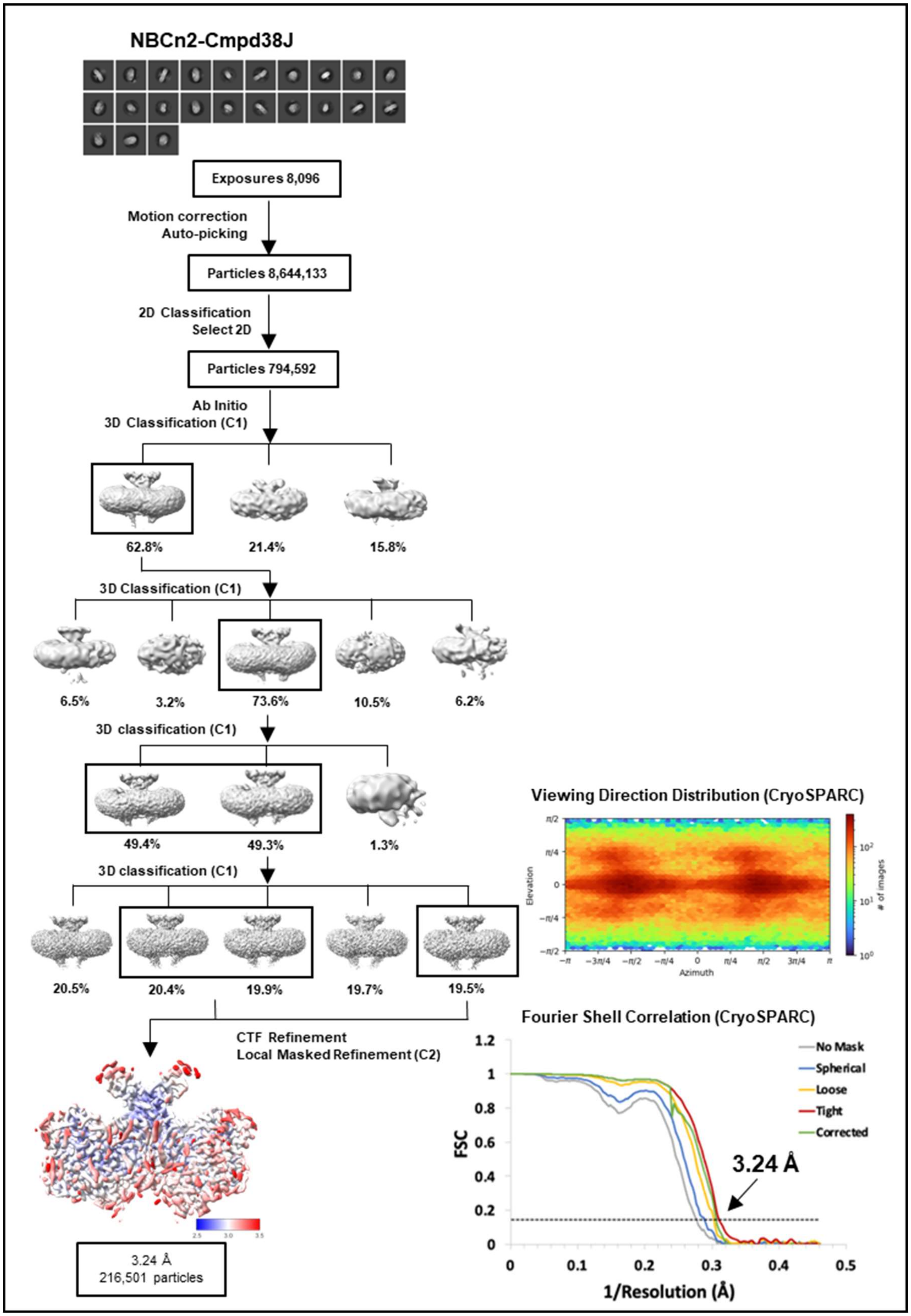
NBCn2-Cmpd38J
Exposures 8,096
Motion correction
Auto-picking
Particles 8,644,133
2D Classification
Select 2D
Particles 794,592
Ab Initio
3D Classification (C1)
62.8%
21.4%
15.8%
3D Classification (C1)
6.5%
3.2%
73.6%
10.5%
6.2%
3D classification (C1)
49.4%
49.3%
1.3%
3D classification (C1)
20.5%
20.4%
19.9%
19.7%
19.5%
CTF Refinement
Local Masked Refinement (C2)
3.24 Å
216,501 particles
Viewing Direction Distribution (CryoSPARC)
Elevation
Azimuth
# of images
Fourier Shell Correlation (CryoSPARC)
No Mask
Spherical
Loose
Tight
Corrected
FSC
3.24 Å
1/Resolution (Å)

Supplementary Fig. 9 | CryoEM structure determination of inhibitor-bound NBCn2. Data were collected on 300 keV Krios and processed in cryoSPARC: particles were picked from motion corrected micrographs, subjected to 2D classification (representative classes are shown), followed by ab initio model building and 3D classification. After multiple rounds of 3D classification, the final particle stack was subjected to local CTF refinement followed by local refinement of the masked NBCn2 membrane domain with imposed C2 symmetry. Final map was obtained with GS-FSC indicating a global resolution of 3.24 Å applying the 0.143 cutoff. The model was built and refined in PHENIX. Calculations in cryoSPARC indicate local resolutions of up to 2.7 Å around substrate binding site. Viewing direction analysis (cryoSPARC) indicates sufficient coverage and isotropic distribution of views in final particle stack. Source data are provided as a Source Data file.

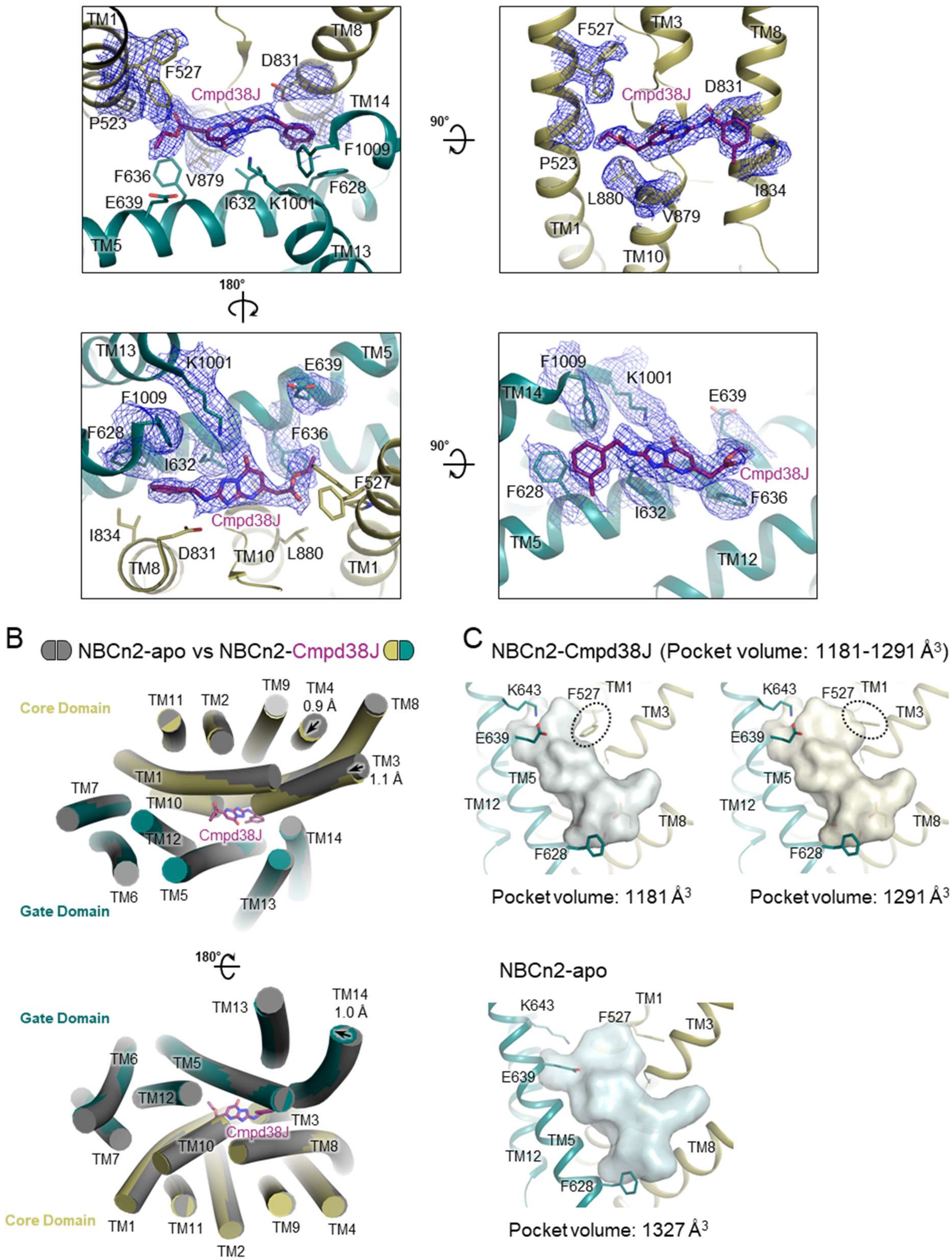

TM1
F527
D831
TM8
Cmpd38J
TM14
P523
F1009
F636
V879
F628
E639
I632
K1001
TM5
TM13
90°
F527
TM3
TM8
Cmpd38J
D831
P523
L880
V879
I834
TM1
TM10
180°
TM13
K1001
E639
TM5
F1009
F628
F636
I632
F527
Cmpd38J
I834
TM8
D831
TM10
L880
TM1
90°
F1009
K1001
TM14
E639
Cmpd38J
F628
F636
I632
TM5
TM12
B
NBCn2-apo vs NBCn2-Cmpd38J
Core Domain
TM11
TM2
TM9
TM4
0.9 Å
TM8
TM3
1.1 Å
TM1
TM7
TM10
TM12
Cmpd38J
TM14
TM6
TM5
TM13
Gate Domain
180°
Gate Domain
TM13
TM14
1.0 Å
TM6
TM5
TM12
Cmpd38J
TM3
TM7
TM10
TM8
Core Domain
TM1
TM11
TM9
TM4
TM2
C
NBCn2-Cmpd38J (Pocket volume: 1181-1291 Å³)
K643
F527
TM1
TM3
E639
TM5
TM12
TM8
F628
Pocket volume: 1181 Å³
Pocket volume: 1291 Å³
NBCn2-apo
K643
F527
TM1
TM3
E639
TM5
TM12
TM8
F628
Pocket volume: 1327 Å³

Supplementary Fig. 10 | Representative cryoEM density of inhibitor-bound NBCn2 and analysis of binding pocket volume. A, CryoEM densities (marine mesh) for Cmpd38J and nearby transporter residues in the NBCn2 inhibitor binding site using a contour level of 6σ. **B**, Global structural changes between apo- (gray) and Cmpd38J-bound NBCn2 (colored by domain). **C**, Calculation of pocket volume in substrate-bound NBCn2, with distinct F527 rotamer states resulting in distinct pocket volumes (top). Pocket volume in apo-NBCn2 for comparison (bottom).

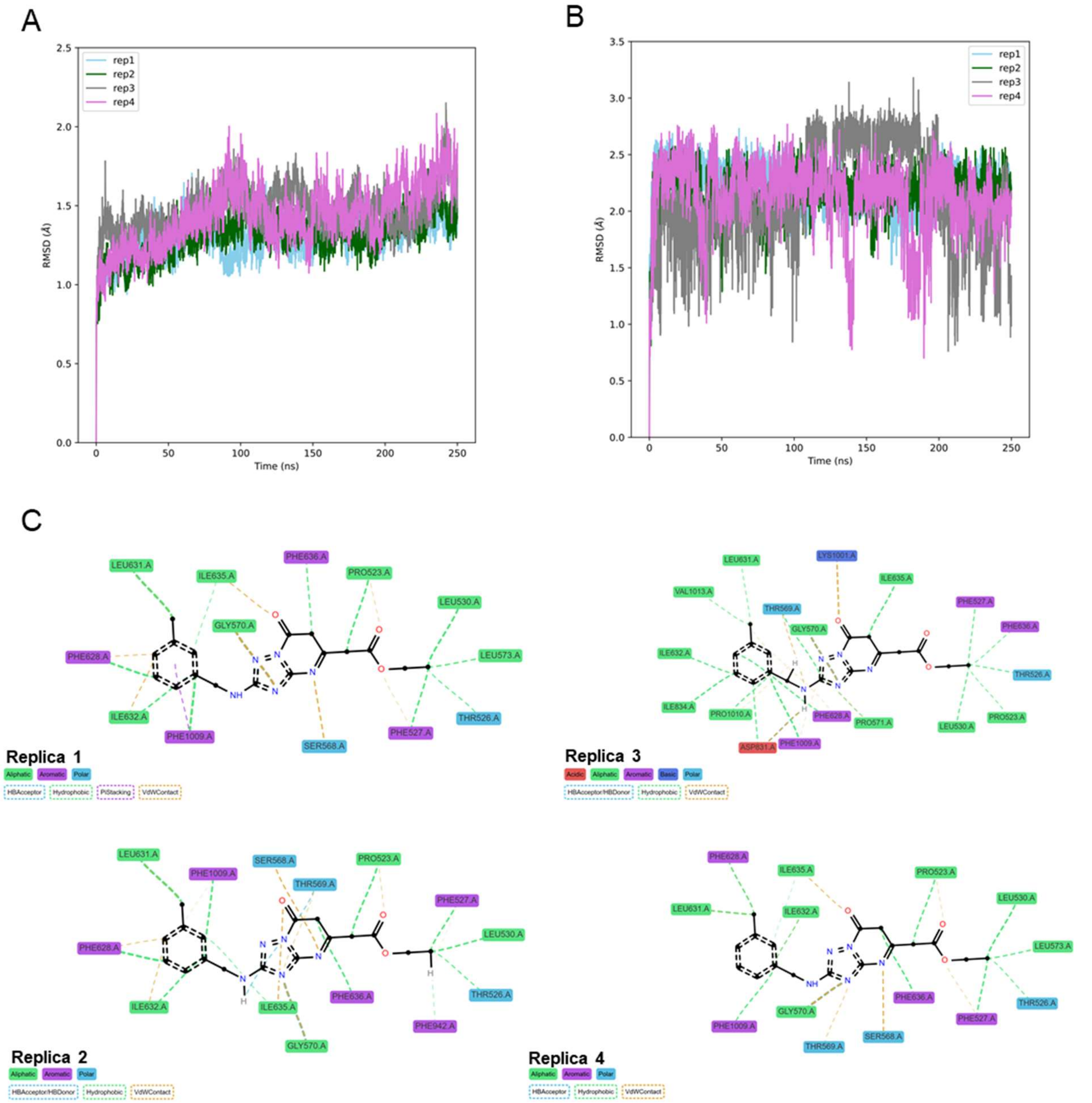

Supplementary Fig. 11 | Analysis of inhibitor binding at NBCn2 via MD simulation. **A**, RMSD values of the transmembrane domain regions of NBCn2 from the MD simulations of NBCn2 with 4 replicas. **B**, RMSD values of Cmpd38J from the MD simulations of NBCn2 with 4 replicas. **C**, Molecular interaction between NBCn2 and Cmpd38J over the simulation time for each replica. Source data are provided as a Source Data file.

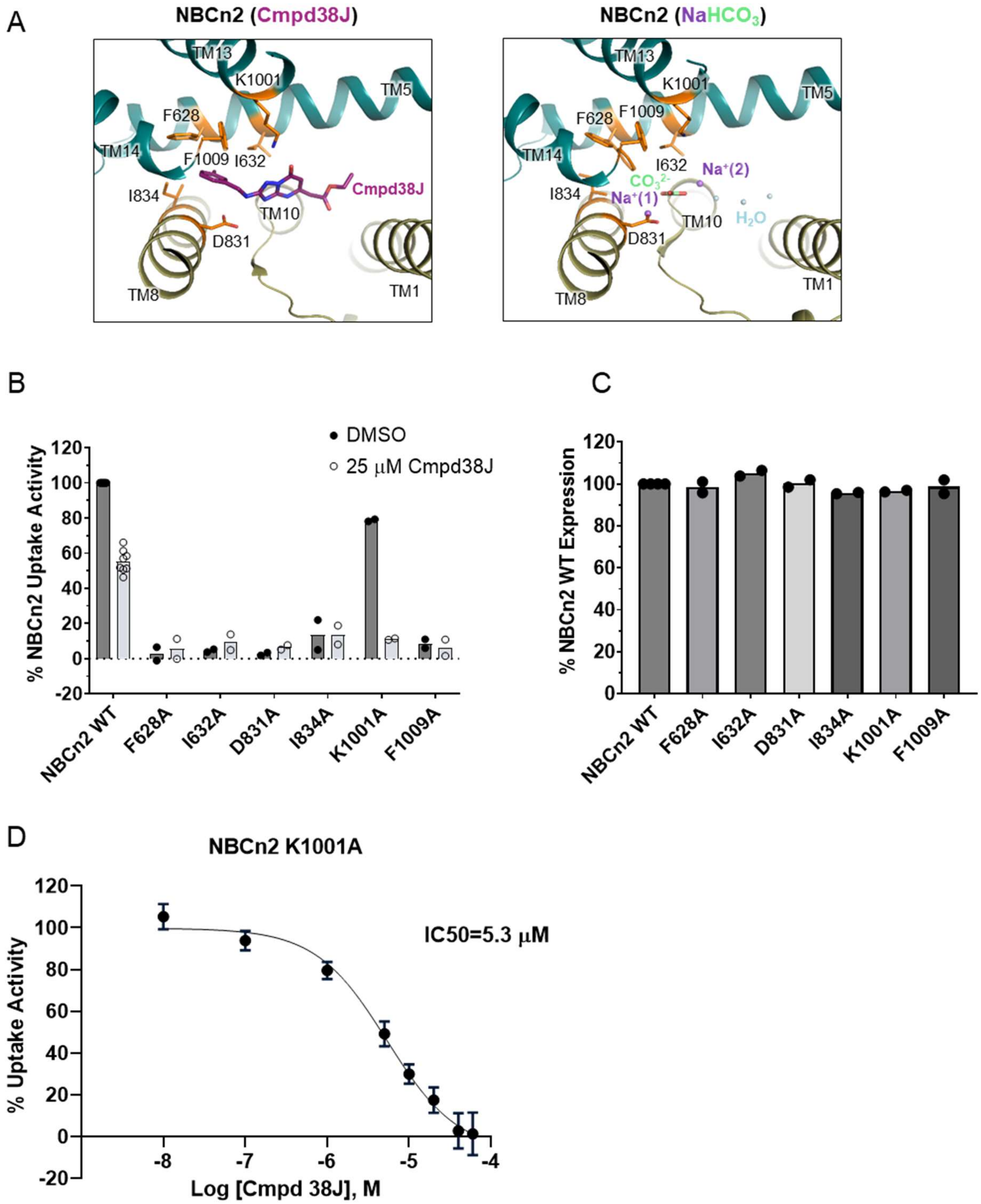

A
NBCn2 (Cmpd38J)
TM13
K1001
TM5
F628
TM14
F1009
I632
Cmpd38J
I834
TM10
D831
TM8
TM1
NBCn2 (NaHCO3)
TM13
K1001
TM5
F628
F1009
TM14
I632
Na+(2)
CO3 2-
I834
Na+(1)
TM10
H2O
D831
TM8
TM1
B
% NBCn2 Uptake Activity
DMSO
25 μM Cmpd38J
NBCn2 WT
F628A
I632A
D831A
I834A
K1001A
F1009A
C
% NBCn2 WT Expression
NBCn2 WT
F628A
I632A
D831A
I834A
K1001A
F1009A
D
NBCn2 K1001A
% Uptake Activity
IC50=5.3 μM
Log [Cmpd 38J], M

Supplementary Fig. 12 | Structure-activity relationship (SAR) studies probing Cmpd38J-NBCn2 interactions via site-directed mutagensis. A, Key residues in the NBCn2 substrate binding site interacting with Cmpd38J. **B**, NBCn2 uptake studies in the presence and absence of 25 µM Cmpd38J or vehicle. Experiments were performed with 4 technical repeats and are averaged from n=2 independent experiments for the mutants, and n=8 independent experiments for wildtype NBCn2. Data are represented as mean ± s.e.m, and have been normalized to wildtype NBCn2-mediated uptake in the presence of vehicle. **C**, ELISA measuring the expression level of wildtype and mutant NBCn2. Experiments were performed with 6 technical repeats and are averaged from =2 independent experiments for the mutants and n=4 for wildtype NBCn2. Data are represented as mean ± s.e.m, and have been normalized to wildtype NBCn2 expression levels. **D**, Concentration-response studies of Cmpd38J at the NBCn2 K1001A mutant. Experiments were performed with 4 technical repeats and are averaged from n=4 independent experiments. Data are represented as mean ± s.e.m and have been normalized. Source data are provided as a Source Data file.

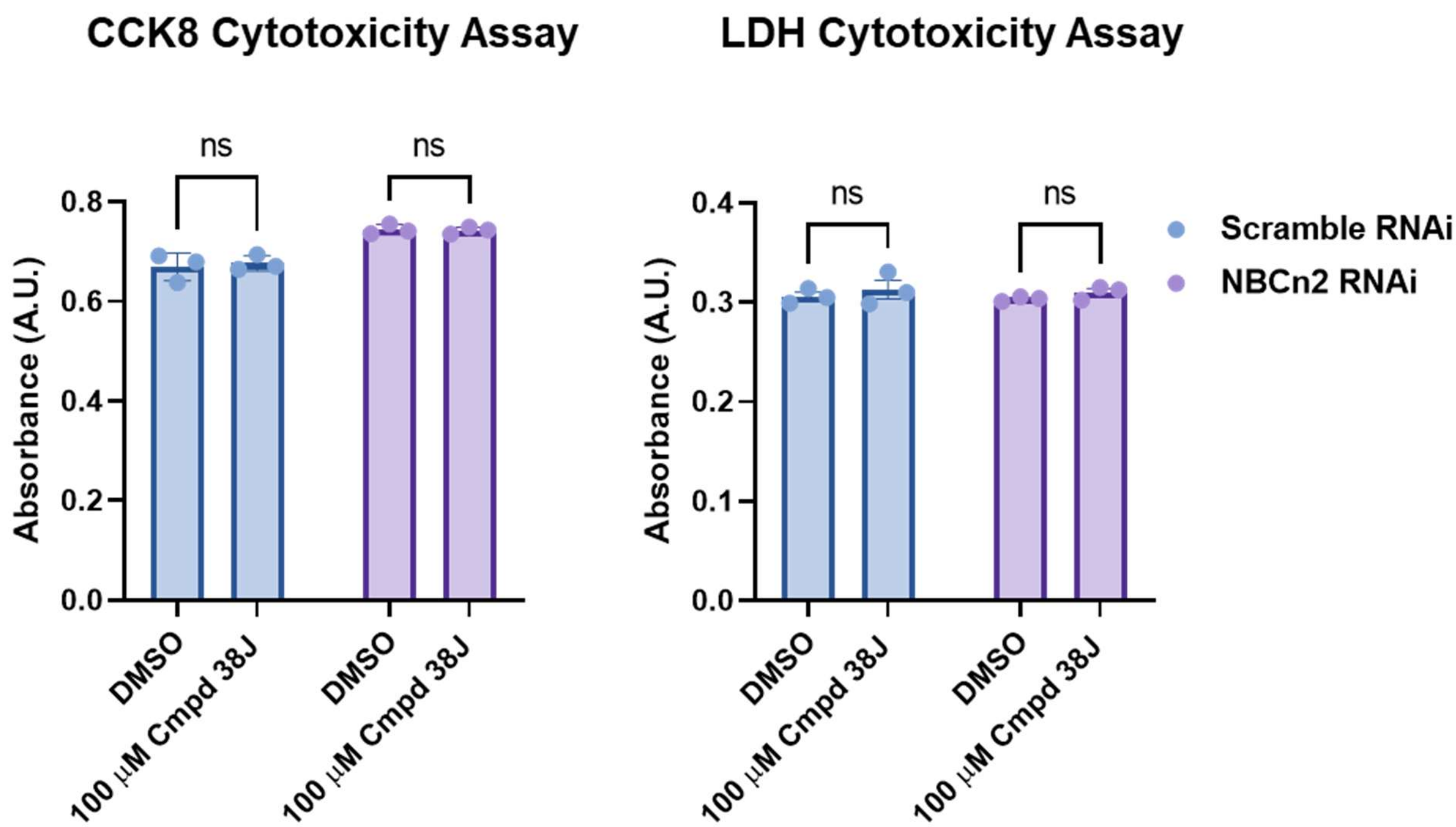

Supplementary Fig. 13 | Viability of primary neuron-glia co-culture treated with antisense oligos and drug. Cellular toxicity of treatments was determined via CCK8 and LDH cytotoxicity assays. Experiments were performed with 3-7 technical repeats and are averaged from n=3 independent experiments. Data are represented as mean ± s.e.m. Differences between DMSO and drug treated groups are not statistically significant (ns) according to two-way analysis of variance using Fisher's least significance difference post hoc test. CCK8: Scramble:DMSO vs Scramble:100 μM Cmpd38J (P = 0.6650); NBCn2:DMSO vs NBCn2:100 μM Cmpd38J (P = 0.8443). LDH: Scramble:DMSO vs Scramble:100 μM Cmpd38J (P = 0.3047); NBCn2:DMSO vs NBCn2:100 μM Cmpd38J (P = 0.1459). Source data are provided as a Source Data file.

Supplementary Table 1 | Cryo-EM data collection, refinement and validation statistics.

	**NBCn2-Apo** (EMD-48304) (PDB 9MIX)	**NBCn2-NaHCO$_3$** (EMD-48318) (PDB 9MJO)	**NBCn2-Cmpd38J** (EMD-48320) (PDB 9MK6)
**Data collection and processing**			
Voltage (kV)	300	300	300
Electron exposure (e^{-}/Å^2)	61.36	50.00	52.48
Defocus range (μm)	-0.5 to -1.8	-0.5 to -1.8	-0.5 to -1.8
Pixel size (Å)	0.833	0.4125	1.069
Symmetry imposed	C2	C2	C2
Initial particle images (no.)	5086362	2529664	8644133
Final particle images (no.)	536488	291402	216501
Map resolution (Å)	2.85	2.45	3.24
FSC threshold	0.143	0.143	0.143
Map sharpening B-factor (Å^2)	-169.3	-79.5	-150.7
**Refinement**			
Model composition			
Non-hydrogen atoms	9120	8638	9190
Protein residues	1134	1076	1142
Ligands	12	14	12
R.m.s. deviations			
Bond lengths (Å)	0.006	0.004	0.004
Bond angles (°)	0.800	0.589	0.614
Validation			
Clashscore	5	6.37	4.36
Poor rotamers (%)	0.00	0.00	0.00
Ramachandran plot			
Favored (%)	96.79	97.92	98.07
Allowed (%)	3.21	2.08	1.93
Disallowed (%)	0	0	0

Supplementary Table 2 | MD simulation system set up.

	**Cmpd38J-bound NBCn2**	**Substrate-bound NBCn2 (Na^{+}-Na^{+}-CO_3^{2-})**
Simulation box	110 × 110 × 132 Å	110 × 110 × 133 Å
Number of atoms	148604	157323
Number of waters	33363	34130
Salt concentration	0.15M NaCl	0.15M NaCl
Number of lipids	150:142 POPC	150:144 POPC

Supplementary Table 3 | Experimentally tested compounds from virtual ligand screen.

Compound	Vendor	Catalog Number	SMILES
Cmpd24	Molport	6343792	Nc1ccc2[nH]c(=O)nc(-c3ccccc3)c2c1
Cmpd25	Molport	30523456	N#Cc1cccnc1-c1ccc2[nH]ccc2c1
Cmpd26	Molport	99246929	Cc1[nH]c(=O)[nH]c(=O)c1-c1ccc2[nH]ccc2c1
Cmpd27	Molport	F6246-0812	Cc1ccccc1NC(=O)Cn1nc(-c2cccnc2)n(C2CC2)c1=O
Cmpd28	Molport	F5458-0060	O=C(NCCn1ncccc1=O)Nc1ccc2OCOc2c1
Cmpd29	Molport	F6258-1217	Clc1ccccc1\C=C\C(=O)NCCn1cnc(cc1=O)C1CC1
Cmpd30	Molport	3685600	Cc1[nH]c(=O)c(N)cc1-c1ccccc1
Cmpd31	Molport	7010120564	Cc1ccc(N2CC(=O)C(=C2N)c2nc3ccccc3c(=O)[nH]2)c(C)c1 |c:9|
Cmpd32	Molport	STL352345	CC(=O)Nc1cccc(c1)-c1n[nH]c(=S)n1C
Cmpd33	Molport	STK243552	COCc1n[nH]c(N)c1-c1ccc(OC)cc1
Cmpd34	Molport	STL259858	COCc1n[nH]c(N)c1-c1cccc(OC)c1
Cmpd35	ChemDiv	D043-0065	Cc1ccc(CNc2nc3[nH]c(C)c(C)c(=O)n3n2)cc1
Cmpd36	ChemDiv	D043-0072	Cc1nc2nc(NCc3ccccc3F)nn2c(O)c1C
Cmpd37	ChemDiv	D205-0475	O=C(Nc1nc2[nH]c(-c3ccccc3)cc(=O)n2n1)c1ccccc1F
Cmpd38	ChemDiv	D298-0389	COC(=O)Cc1cc(=O)n2nc(NCc3cc(F)cc(F)c3)nc2[nH]1
Cmpd39	ChemDiv	D298-0391	CCc1cc(=O)n2nc(NCc3cc(F)cc(F)c3)nc2[nH]1
Cmpd40	ChemDiv	D412-0453	CCc1ccc(CNc2nc3[nH]c(CNC(C)C)cc(=O)n3n2)cc1
Cmpd41	ChemDiv	P574-2341	CCc1nc(N)n(CC(=O)Nc2ccc(F)c(F)c2)c(=O)c1C
Cmpd42	ChemDiv	P574-2363	CCc1nc(N)n(CC(=O)Nc2ccc(F)cc2C)c(=O)c1C
Cmpd43	ChemDiv	P574-4689	COCc1cc(=O)n(CC(=O)Nc2ccc(Cl)cc2)c(N)n1
Cmpd44	ChemDiv	P574-4690	COCc1cc(=O)n(CC(=O)Nc2ccc(F)cc2Cl)c(N)n1
Cmpd45	ChemDiv	Y021-4671	C[C@@H]1SC(NC(=O)COc2ccccc2)=NC1=O
Cmpd46	ChemDiv	Y204-1012	O=C(O)[C@H]1[C@@H]2C=C[C@@H](C2)[C@@H]1C(=O)N1CCN(Cc2ccccc2)CC1
Cmpd47	ChemDiv	Y205-7957	O=C(O)[C@@H]1[C@@H](C(=O)N2CCN(C3CCCCC3)CC2)[C@@H]2C=C[C@H]1C2
Cmpd48	ChemDiv	Y205-7965	O=C(O)[C@H]1C2CCC(CC2)[C@@H]1C(=O)N1CCN(C2CCCCC2)CC1
Cmpd49	Enamine	Z51438291	FC1=CC=C(NC(=O)CN2C=NC3=CC=C(Cl)C=C3C2=O)C(F)=C1
Cmpd50	Enamine	Z608550276	CC=1C=CC=CC1CNC=2C=CC=C3NN=CC23
Cmpd51	Enamine	Z28139772	O=C(CN1C=NC=2C=CC=CC2C1=O)NC3=CC=CC(Cl)=C3
Cmpd52	Enamine	Z928770442	O=C1CC2(CCSC2)C(=O)N1CC(O)COC=3C=CC(F)=CC3
Cmpd53	Enamine	Z2203549027	OCC(=O)NC1COC2=CC=C(F)C=C2C1
Cmpd54	Enamine	Z95681301	O=C(CC1=CSC(=N1)N2CCCC2=O)NCC3=CC=CC=C3F
Cmpd55	Enamine	Z54321775	CC=1NC2=CC=CC=C2C1C=NNC(=N)N
Cmpd56	Enamine	Z1082776722	CN1N=CC=2C(=O)NC(=NC21)NCC(O)C=3C=CC(Cl)=CC3
Cmpd57	Enamine	Z5458248283	CN(CC(=O)NCCOC=1C=CC=C(F)C1)C=2N=CN=C3NC=NC23
Cmpd58	Enamine	Z445230638	COCC1=CC=CC(NCC2=CNN=C2C)=C1
Cmpd59	Enamine	Z56977384	FC(F)(F)C1=CC=CC(NC(=O)CC2SC3=NCCN3C2=O)=C1
Cmpd60	Chem-Impex	11050	C1C(=O)N[C@H](C(=O)N1)CC2=CNC3=CC=CC=C32

Supplementary Table 4 | Experimentally tested Cmpd38 analogs.

Compound	Vendor	Catalog Number	SMILES
Cmpd38A	ChemDiv	D298-0001	COC(=O)Cc1cc(=O)n2nc(NCc3ccccc3)nc2[nH]1
Cmpd38B	ChemDiv	D298-0081	COC(=O)Cc1cc(=O)n2nc(NCc3cccc(C)c3)nc2[nH]1
Cmpd38C	ChemDiv	D298-0096	COC(=O)Cc1cc(=O)n2nc(NCc3cccs3)nc2[nH]1
Cmpd38D	ChemDiv	D298-0086	COC(=O)Cc1cc(=O)n2nc(NCc3cccc(Cl)c3)nc2[nH]1
Cmpd38E	ChemDiv	D298-0036	COC(=O)Cc1cc(=O)n2nc(NCc3ccc(Cl)cc3)nc2[nH]1
Cmpd38F	ChemDiv	D298-0056	COC(=O)Cc1cc(=O)n2nc(NCc3ccccc3F)nc2[nH]1
Cmpd38G	ChemDiv	D298-0031	COC(=O)Cc1cc(=O)n2nc(NCc3ccc(F)cc3)nc2[nH]1
Cmpd38H	ChemDiv	D298-0021	COC(=O)Cc1cc(=O)n2nc(NCc3ccc(OC)cc3)nc2[nH]1
Cmpd38I	ChemDiv	D298-0347	COC(=O)Cc1cc(=O)n2nc(NCc3ccc(SC)cc3)nc2[nH]1
Cmpd38J	ChemDiv	D298-0084	CCOC(=O)Cc1cc(=O)n2nc(NCc3cccc(C)c3)nc2[nH]1
Cmpd38K	ChemDiv	D298-0394	CCCc1cc(=O)n2nc(NCc3cc(F)cc(F)c3)nc2[nH]1
Cmpd38L	ChemDiv	D298-0391	CCc1cc(=O)n2nc(NCc3cc(F)cc(F)c3)nc2[nH]1
Cmpd38M	Enamine	Z1611172339	COCCc1ccc2oc(NCc3cc(F)cc(F)c3)nc2c1
Cmpd38N	Enamine	Z1778562164	Cc1cc(C)n2nc(SCc3cc(F)cc(F)c3)nc2n1
Cmpd38O	Enamine	Z3242198883	Fc1cc(F)cc(CNc2nc3cc(Br)ccc3[nH]2)c1
Cmpd38P	Enamine	PV-004461701774	CCOCc1cc(=O)n2nc(NC(=O)CC3=CCCC3)nc2[nH]1
Cmpd38Q	Enamine	PV-003006981438	CCOCc1cc(=O)n2nc(NC(=O)c3ccc(F)cc3)nc2[nH]1
Cmpd38R	Enamine	PV-002228602414	Cc1cc(=O)n2nc(OCc3cc(F)cc(Br)c3F)nc2[nH]1
