# Supplementary material for: Structural insights enable drug discovery for the neuronal NBCn2 carbonate transporter
Source: Nat Commun. 2026 Jul 22;17:8923. doi: 10.1038/s41467-026-75444-4 (PMC13503941; doi:10.1038/s41467-026-75444-4)
Supplement: Supplementary file 3 [file 41467_2026_75444_MOESM3_ESM.pdf]

nature portfolio

Peer Review File

Structural insights enable drug discovery for the neuronal NBCn2 carbonate transporter

Corresponding Author: Dr Daniel Wacker

This file contains all reviewer reports in order by version, followed by all author rebuttals in order by version.

Version 0:

Reviewer comments:

Reviewer #1

(Remarks to the Author)
Presented is a study on the SLC4 transporter, NBCn2, that included determination of the 3D structure of the protein in 2 forms using CryoEM. These structures were then used to undertake MD and SILCS simulations to yield information on the mechanism of the transporter. This was followed by docking against a known site from which active ligands are identified and the binding location verified through determation of an additional CryoEM structure. The need for both structural information as well as ligands that inhibit the protein's activity are important to elucidate details of the biological role of the transporter as well as set the foundation for development of therapeutics. Accordingly, this is an important study with the results being presented in a clear and comprehensive fashion. Some minor concerns follow.

1) The authors briefly mention that the preotein was glycosylated based on the CryoEM data but no additional information is presented. Has Mass Spec analysis been undertaken to verify the that glycosylation is present? Is there a possible functional role? This potential needs to be discussed.

2) Page 11: The Tanimoto values are very low. They are typically presented on percent values from 0 to 100%. Values of 7.1 and 6.1 may be more appropriate though even those values are very low for organic compounds. Please double check this.

3) What force field was used for the ligands in the SILCS and MD simulations?

Reviewer #2

(Remarks to the Author)
First, I must state that I am reviewing this manuscript as a physiologist. I am unable to assess the structural data and trust that the editor has engaged a structural biologist to review that aspect.

As the authors acknowledge, this is not the first SLC4 structure but nonetheless includes some unexpected and important findings that may not have been possible by homology modeling alone.

MAJOR

The number one thing that I'd want to learn from a study such as this is whether NBCn2 is an electroneutral NBC with HCO3-dependent, Cl-Cl self-exchange activity (i.e., Na+-HCO3-Cl/Cl exchange) or a Na+-driven Cl/HCO3 exchanger. The authors will note that even proponents of the first hypothesis find that NBCn2 operates as a Na+-driven Cl-HCO3 exchanger in the absence of extracellular Cl. I feel that this issue is not fully addressed in this study. The presence of two Na+ sites with a single CO3= site, although different from NDCBE, is not anticipated by either hypothesis. Do the authors find any evidence of Cl binding, or an aqueous pathway through which Cl could pass? Are the binding sites symmetrical within a dimer? A greater attempt towards reconciliation of the hypotheses would be helpful.

The authors claim of a selective NBCn2 inhibitor should be validated by testing of the lead compound on other SLC4 proteins. The cell-culture/slice assays are convincing in that regard because both preparations robustly express all three electroneutral NBCs. Is the knockdown of NBCn2 and the addition of drug additive with regards to excitability?

The cell assay conditions are unlikely to be as the authors report. It is not possible to maintain a solution of pH 7.4 and [HCO3] of 25 mM in room air. The relationship between pH/HCO3/pCO2 is described by the Henderson-Hasselbalch equation. If pH is indeed 7.4 then, without substantial atmospheric CO2 to hold it in equilibrium, the majority of the added HCO3 will dissipate as CO2 and only ~0.25mM will remain (another possibility is that pH is much greater than 7.4). Thus the assay is not performed under physiological conditions, nor is it a robust assay because [HCO3] is probably much lower than Kd for the transporter. The authors should carefully re-evaluate their findings if [HCO3] is a critical parameter for interpretation and they should also be clear in their methods that the assay solution contained <1 mM HCO3.

MINOR
Line 1: The use of 'at' in the title is awkward. Suggest rephrasing and suggest including the transporter name.
Line 53: '…other studies..' suggest replacing with '..an independent study..'
Line 85: The authors should clarify whether removing 231-299 leaves a functional transporter.
Line 458: What is the evidence for reversibility?
Figure 6F-H: The statistics appear weaker than anticipated. Would the authors get a more robust outcome, or gain additional insight, by reporting (as a single metric) the drop in frequency of high amplitude events?

Reviewer #3

(Remarks to the Author)
The authors present cryo-EM structures of the brain carbonate/Na+ symporter SLC4A10, highlighting determinants for substrate binding and transport, and further continue to screen for small molecule SLC4A10 inhibitors. Two lead compounds were further investigated, including substituent modifications, IC50 estimation by dose-response curves, and initial screening in physiological models, including neuronal culture and brain slices.

This is an important transporter for regulating intracellular pH and the insights into the structural basis for its mechanism and modulation by small molecules will be of great interest to the transporter community and has further potential in the development of anti-seizure medications.

Overall, this is a solid manuscript, but there are a number of clarifications and further experiments required to validate some of the main conclusions drawn.

Major points

Ion coordination
With only two oxygen atom interactions the coordination of the Na2 site is very unconvincing. Na+ coordination needs at least 5 ligands. Please consider: alchemical perturbation calculations, check-my-metal server, AlphaFold 3 prediction, ITC measurements of mutations and/or transport assays to validate sodium binding at the Na2 site.

Inhibition studies
Please show the extent of inhibition of the small molecules vs. non-expressed protein rather than based on normalization to the compound38 (Fig. 4a). With the normalization currently carried out, its difficult to assess how much of the inhibition in cells might be due to a systemic off-target effect. The IC50 curves do not show a clear sigmoidal dose-response and further concentrations are needed for accurate fitting (Fig. 4b). While the cryo EM maps support the ligand binding of Cmpd 38J, the structural themselves are insufficient to prove that this is its mode of inhibition. Please mutate key coordinating residues to show either reduced or complete loss of inhibition by Cmpd 38J. It still was unclear to me if Cmpd 38J was specific to SLC4A10. Please show the IC50 to the other members NDCBE and AE1.

Minor comments
- Lines 84-85: It would help to include a figure showing, based on the AF3 model, the construct used for structural studies and it's unclear to me the importance of the regions omitted.
- Lines 87-90 or Figure 1: it would be helpful to state that the structures obtained are in LMNG
- Lines 442-445: What are physiological substrate conditions that a drug would have to compete with?

Reviewer #4

(Remarks to the Author)
I co-reviewed this manuscript with one of the reviewers who provided the listed reports. This is part of the Nature Communications initiative to facilitate training in peer review and to provide appropriate recognition for Early Career Researchers who co-review manuscripts.

Version 1:

Reviewer comments:

Reviewer #1

(Remarks to the Author)
The authors have addressed my concerns.

Reviewer #2

(Remarks to the Author)
I thank the reviewers for addressing my concerns. I am satisfied that their thoughtful approach and inclusion of new and revised data have produced a more robust manuscript.

Reviewer #3

(Remarks to the Author)
Overall, the manuscript has been clearly improved, but there are a few remaining issues that we would still like to see resolved.

Major comments

Lines 375-386: The effect observed for K1001A is very interesting. It would make sense to see whether the effect of this mutation is due to an increased affinity for Cmpd38J or due to a different reason. A rudimentary IC50 determination would be helpful to be able to discuss this mutation in more detail, especially because this mutation/effect is not touched on again at all in the discussion.

Lines 181-183: To make this statement, the same predictions/analyses without HCO3 and/or the waters should result in an unchanged Na1 site and the loss of Na2. This can be checked very quickly with AF.

Figure 4A: I would consider making the bars greyscale and only coloring the bars for 38D, 38J, and 38Q, which will make it easier to highlight these particularly strong inhibitors.

Minor comments

Figure 1D the NaHCO3 label is misaligned
Lines 310-313: I would add that this rather affects an accurate prediction of Cmpd38Js affinity rather than selectivity – you show that it is selective for NBCn2 and NDCBE over AE1
Throughout the manuscript: Misspelled propionate
Extended Data Figure 12 B: It would be easier to view this data with residue labels under the axis and two separate colors for DMSO/Cmpd38J. This would reduce the number of colors and make it easier to link the residues to the bars.

Reviewer #4

(Remarks to the Author)
I co-reviewed this manuscript with one of the reviewers who provided the listed reports. This is part of the Nature Communications initiative to facilitate training in peer review and to provide appropriate recognition for Early Career Researchers who co-review manuscripts.

Version 2:

Reviewer comments:

Reviewer #3

(Remarks to the Author)
Im more than satisfied with the final updates to the paper and have no furher concerns.

Reviewer #4

(Remarks to the Author)
I co-reviewed this manuscript with one of the reviewers who provided the listed reports. This is part of the Nature Communications initiative to facilitate training in peer review and to provide appropriate recognition for Early Career

Researchers who co-review manuscripts.

Open Access This Peer Review File is licensed under a Creative Commons Attribution 4.0 International License, which permits use, sharing, adaptation, distribution and reproduction in any medium or format, as long as you give appropriate credit to the original author(s) and the source, provide a link to the Creative Commons license, and indicate if changes were made.
In cases where reviewers are anonymous, credit should be given to 'Anonymous Referee' and the source.
The images or other third party material in this Peer Review File are included in the article's Creative Commons license, unless indicated otherwise in a credit line to the material. If material is not included in the article's Creative Commons license and your intended use is not permitted by statutory regulation or exceeds the permitted use, you will need to obtain permission directly from the copyright holder.
To view a copy of this license, visit https://creativecommons.org/licenses/by/4.0/

We would like to thank the reviewers for their thorough investigation of our work, as well as their helpful suggestions and comments. We apologize for the delay in providing a revised version, but the requested experiments, particularly the cloning and generation of cell lines of NBCn2 mutants and the studies in neurons are very time consuming. We would also like to note that during revision, our previous studies of how NBCn2 knockdown reduces neuronal activity in primary culture could not be reproduced, and our studies show that RNAi toxicity was the likely problem. We apologize for this, and have since performed more thorough experiments that are described in detail in our response to reviewer 2 and have been included in the revised manuscript. We would like to point out that these finding do not change the overall thesis or findings of our work and, in fact, add significant depth and rigor to this study.

Reviewer #1 (Remarks to the Author):

Presented is a study on the SLC4 transporter, NBCn2, that included determination of the 3D structure of the protein in 2 forms using CryoEM. These structures were then used to undertake MD and SILCS simulations to yield information on the mechanism of the transporter. This was followed by docking against a known site from which active ligands are identified and the binding location verified through determation of an additional CryoEM structure. The need for both structural information as well as ligands that inhibit the protein's activity are important to elucidate details of the biological role of the transporter as well as set the foundation for development of therapeutics. Accordingly, this is an important study with the results being presented in a clear and comprehensive fashion. Some minor concerns follow.

1) The authors briefly mention that the preotein was glycosylated based on the CryoEM data but no additional information is presented. Has Mass Spec analysis been undertaken to verify the that glycosylation is present? Is there a possible functional role? This potential needs to be discussed.

We did not perform any mass spec analysis to assess glycosylation of the protein either produced from Sf9 cells for structural studies, or expressed in HEK cells for uptake experiments. Previous reports have speculated (without evidence) that NBCn2 glycosylation is required for efficient folding and trafficking (PMID: 18061361), but other studies have shown that the function of other SLC4 transporters such as NBCe1 (PMID: 12604466) and AE1 (PMID: 8019599) is unaffected by removal of their glycosylation. We have now included a brief statement in the revised manuscript discussing these findings (Line 99-102).

2) Page 11: The Tanimoto values are very low. They are typically presented on percent values from 0 to 100%. Values of 7.1 and 6.1 may be more appropriate though even those values are very low for organic compounds. Please double check this.

We thank the Reviewer for this thoughtful comment. We would like to clarify that the Tanimoto coefficients reported in the manuscript are expressed as decimal values, not percentages. Thus, values such as 0.071 and 0.061 correspond to 7.1% and 6.1%, respectively.

To improve clarity and prevent confusion, we have revised the Methods section to include the following sentence: “Tanimoto values were calculated using Morgan fingerprints and the Tanimoto similarity function in RDKit. All similarity values are reported as decimals.” (Line 839-841)

3) What force field was used for the ligands in the SILCS and MD simulations?

We thank the Reviewer for this important question. The CHARMM General Force Field was used for fragment molecules in the SILCS simulations and for compound 38J in the MD simulations.

To clarify this in the manuscript, we have revised the Methods section to include the following:

“The CHARMM36 force field was used for the protein, lipids, and ions, and the CHARMM General Force Field was used for the fragment molecules, as described previously.” (Lines 787-789)

“The CHARMM General Force Field was used for Cmpd38J.” (Line 819-820)

Reviewer #2 (Remarks to the Author):

First, I must state that I am reviewing this manuscript as a physiologist. I am unable to assess the structural data and trust that the editor has engaged a structural biologist to review that aspect.

As the authors acknowledge, this is not the first SLC4 structure but nonetheless includes some unexpected and important findings that may not have been possible by homology modeling alone.

MAJOR

The number one thing that I'd want to learn from a study such as this is whether NBCn2 is an electroneutral NBC with HCO3-dependent, Cl-Cl self-exchange activity (i.e., Na+-HCO3-Cl/Cl exchange) or a Na+-driven Cl/HCO3 exchanger. The authors will note that even proponents of the first hypothesis find that NBCn2 operates as a Na+-driven Cl-HCO3 exchanger in the absence of extracellular Cl. I feel that this issue is not fully addressed in this study. The presence of two Na+ sites with a single CO3= site, although different from NDCBE, is not anticipated by either hypothesis. Do the authors find any evidence of Cl binding, or an aqueous pathway through which Cl could pass? Are the binding sites symmetrical within a dimer? A greater attempt towards reconciliation of the hypotheses would be helpful.

The reviewer raises an important issue, however, we have purposefully limited interpretation of our data in the context of transport mechanisms. This is due to i) our explicit focus on developing novel SLC4 probes, as well as ii) caveats of herein presented structures that confine what can be learned about transport. First, we are very confident in our finding of an OF state of NBCn2 being able to accommodate two Na^+ ions and one CO_3^{2-} ion. That being said, these structures are static snapshots, and, in the absence of NBCn2 structures determined in different states (e.g. IF state) or in the presence of different ion compositions/concentrations, it is challenging to make reliable assumptions from a static structure about which ions are even being transported.

Sodium:

For instance, while CO_3^{2-} is likely translocated during the transport cycle, it remains unknown whether both sodium ions, only one sodium ion, or perhaps neither of the sodium ions are being translocated. Wang et al report on Na^+ flux (PMID: 10993873), so we surmise based on this work that at least one of the sodium ions we observe is being transported. For instance, hypotheses that only a single Na^+ ion is co-transported with CO_3^{2-}, while the other remains bound as a co-factor (e.g. Na^+(1)) or simply serves as a charge to guide the anion to the substrate binding site (e.g. Na^+(2)), are conceivable. Proposed models of Na^+-driven Cl^-/HCO_3^- exchange thus do not necessarily disagree with our findings.

On the other hand, our data does not agree with another study reporting on an estimated 1:2 $Na^+:HCO_3^-$ transport stoichiometry (PMID: 14592810). While we can only speak to binding in the OF state, we do not observe a second bound CO_3^{2-}/HCO_3^- ion in our structure. Based on our structural observation, a 1:2 $Na^+:HCO_3^-$ ratio could only be accomplished by two consecutive translocation cycles AND the unlikely event that one cycle is accompanied by Na^+ co-transport while the other is not.

Chloride:

We do not see any density that we could reliably interpret as chloride in either our "apo" or substrate-bound structure, and both structures were obtained in the presence of 100 mM NaCl. We also do not observe any additional cavities or paths through which chloride (or any other ion) could pass, outside the canonical substrate-binding site. Since closely related transporters such as SLC4A1/AE1 are capable of transporting Cl^- via the canonical substrate binding site, it seems unlikely that NBCn2 would evolve a second "channel" to transport or conduct Cl^-.

The canonical substrate binding sites within the individual protomers of the dimer are symmetrical by definition: we applied C2 symmetry during structure refinement to accomplish the resolutions necessary to reliably interpret ion and water densities, which renders both protomers identical in structure and composition. That being said, the clarity and strength of the ion densities suggests that both protomers contain nearly identical ion configurations, as averaging two structurally or compositionally distinct pockets would severely impact

quality and resolution. Because of the absence of any evidence for Cl^-, we simply cannot speak to the role of this ion in NBCn2-mediated transport since the absence of proof is not proof of absence.

Lastly, we would like to reiterate that the goal of our structural work was to elucidate the NBCn2 substrate-binding site for subsequent development of small molecule NBCn2 inhibitors. Mechanistic studies that bring more clarity to NBCn2's transport mechanisms will require determination of additional transporter states obtained in the presence of distinct ion concentrations, and have to be paired with rigorous functional work. We have now attempted to clarify the points above in the discussion of the revised manuscript (Lines 470-478) as well as the results (Lines 224-226). We further pointed out that more structures are required to provide direct molecular insight into the elusive role of Cl^- at NBCn2 or transport mechanisms in general (Line 478-481).

The authors claim of a selective NBCn2 inhibitor should be validated by testing of the lead compound on other SLC4 proteins. The cell-culture/slice assays are *not* convincing in that regard because both preparations robustly express all three electroneutral NBCs. Is the knockdown of NBCn2 and the addition of drug additive with regards to excitability?

We thank the reviewer for this helpful comment. We would like to point out that we did not claim to have developed a selective NBCn2 inhibitor. This would be an unrealistic goal given that NBCn2 is an entirely undrugged and undercharacterized protein, with this seemingly being the first study focused on NBCn2-targeted probe discovery. That being said, inspired by the reviewers comment (and that of reviewer 3), we tested compound's ability to bind other transporters, and have now determined the activity of cmpd38J at both AE1(SLC4A1), a mechanistically distinct SLC4 transporter, as well as the closely related transporter NDCBE(SLC4A8) (Fig A). Unsurprisingly, Cmpd38J does not inhibit AE1 at the concentrations tested, but Cmpd38J does inhibit NDCBE with a slightly lower IC50 of ~40 µM compared to an IC50 of ~20 µM at NBCn2 (Fig A). Due to i) potential differences in substrate Km at different transporters, and ii) assay conditions optimized for throughput that do not allow to accurately control substrate concentrations, these data, however, do not provide a way to accurately quantify the selectivity of one transporter over others. This data has now been included as Figure 4D in the revised manuscript, and a description of the results has been added in Line 305-313.

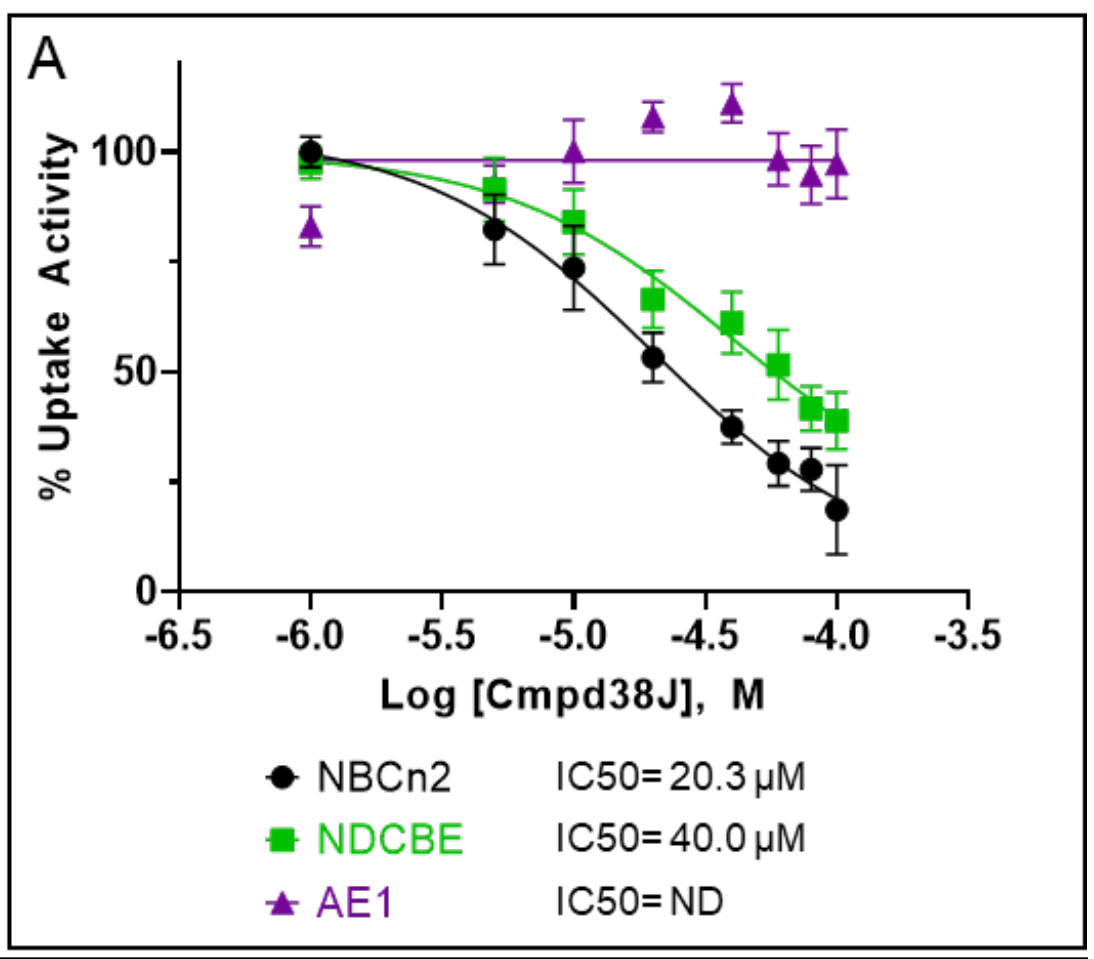

Fig A | Cmpd38J inhibits NBCn2 and NDCBE, but not AE1.

We further wanted to explore the reviewer's excellent point regarding additivity of cmpd38J and NBCn2 knockdown. When we set up for these experiments, a lab member noticed that the previously used RNA concentrations of 4 µM were toxic to the neurons, regardless of whether NBCn2 antisense RNA or control RNA was used (Fig B). We have thus removed all originally reported data collected in primary neurons from the manuscript, and performed more comprehensive experiments: First, we procured new batches of RNA from the vendor and established new RNA treatment conditions that were not toxic to the cells. We ultimately settled on a new protocol in which we treated neurons on DIV14 and DIV17 with 1 µM RNA, and performed Cmpd38J or vehicle treatment on DIV19 (Fig. C). Under these conditions, the neurons did not show any reduced viability as measured by two orthogonal assays (Fig D), but NBCn2 mRNA levels were reduced by ~50% (Fig E).

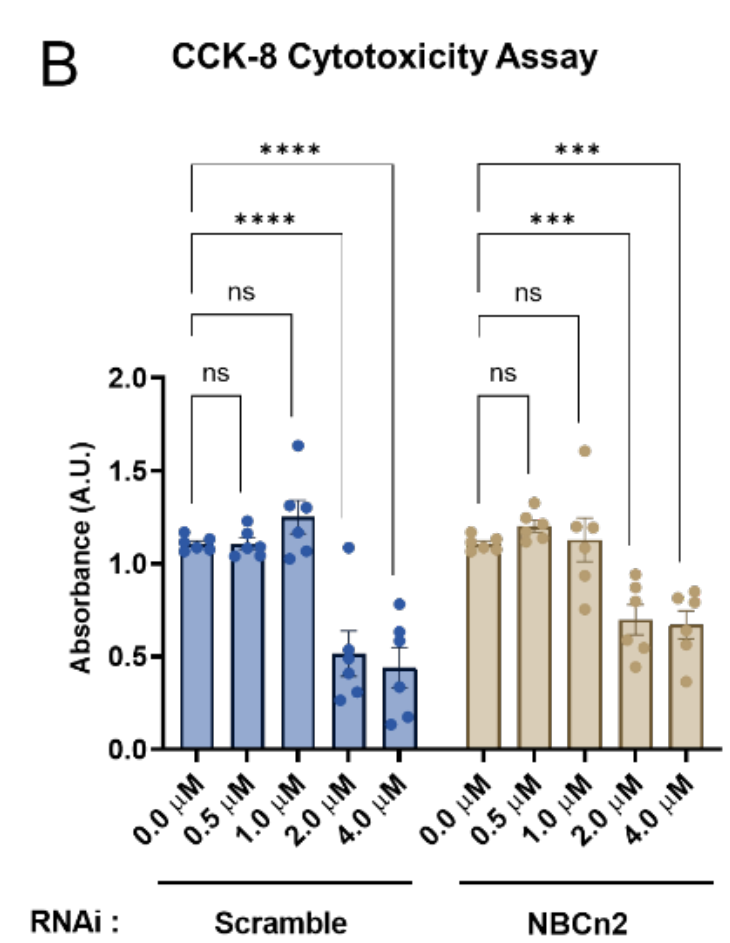

Fig B | Toxicity of different RNAi concentrations in primary neurons

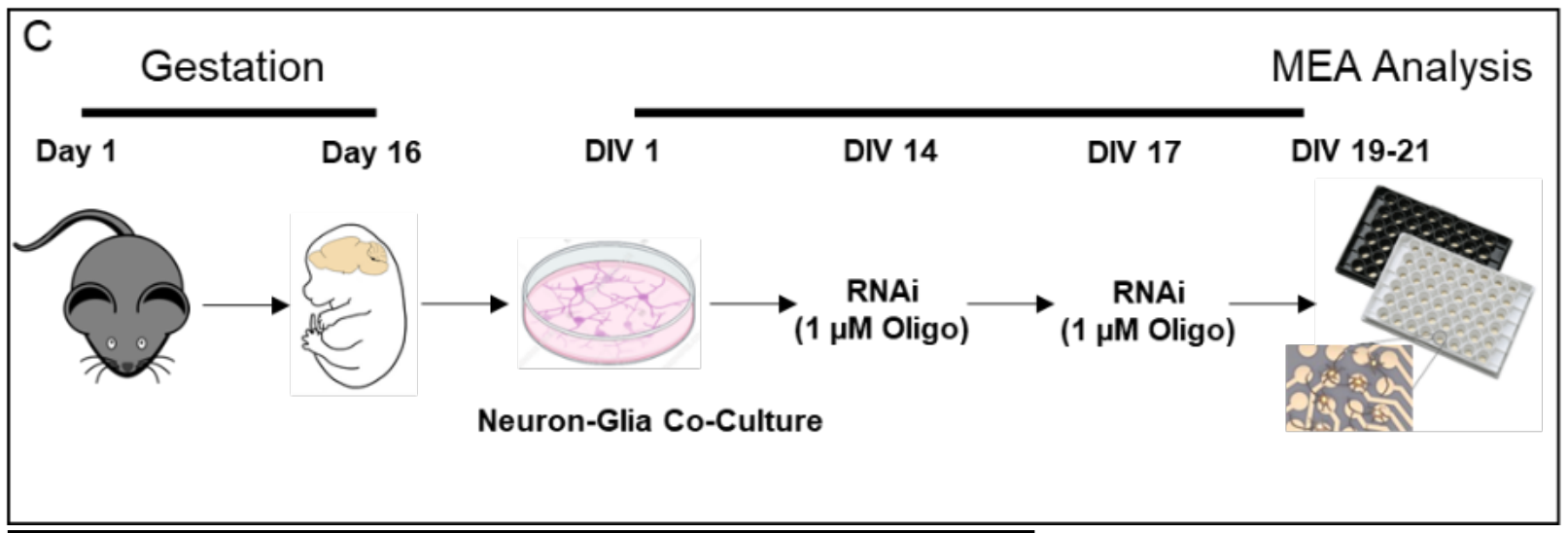

Fig C | New RNAi treatment protocol

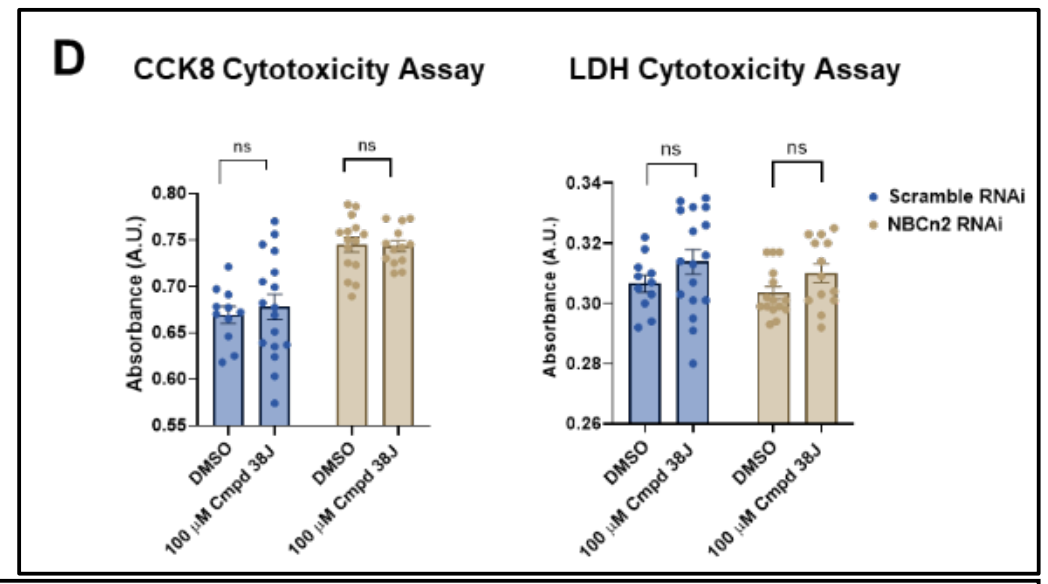

Fig D | Combined RNAi/Drug treatment is not toxic in primary neurons

That being said, when we quantified neuronal activity at DIV19 using our multi-electrode array assay, we did not observe any differences in cells treated with NBCn2 antisense RNA compared to neurons treated with the control RNA in the absence of drug treatment (Fig F). We nonetheless expanded our previous experiments and measured neuronal activity in neurons treated with control RNA and NBCn2 antisense RNA at different time points following treatment with vehicle or Cmpd38J (Fig G). We do observe a sustained reduction in neuronal activity 30 min, 24 hrs, and 48 hrs following treatment with Cmpd38J but not vehicle in both neurons treated with NBCn2 antisense RNA or control RNA (Fig G). Curiously, in the 30 min condition, Cmpd38J treatment does indeed show a lower neuronal activity in NBCn2 knockdown neurons compared to control neurons. This potentially indicates an acute additive effect of NBCn2 knockdown and pharmacological inhibition, but the effect is not observed at 24 hrs or 48 hrs.

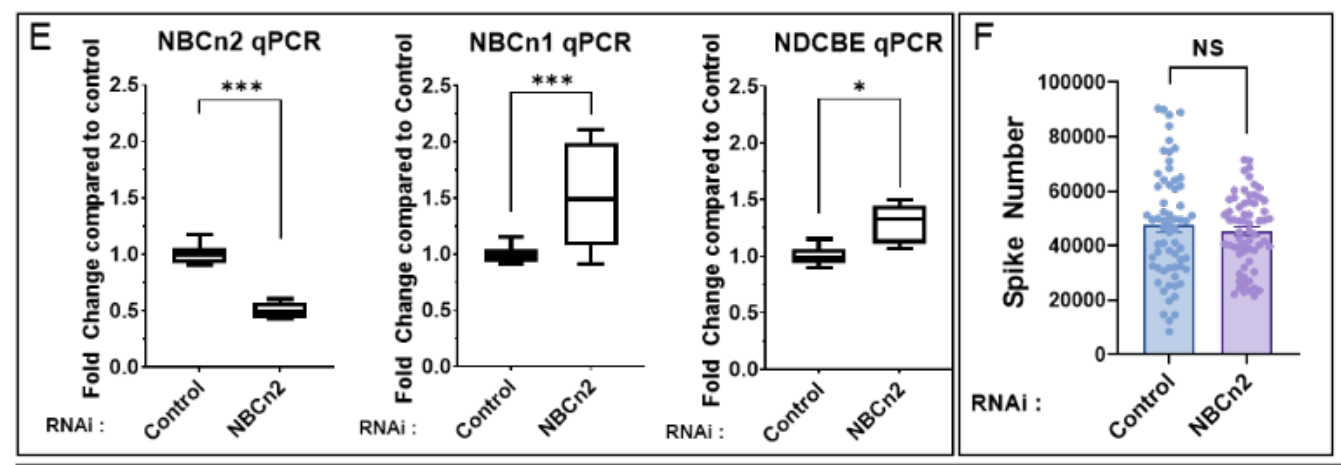

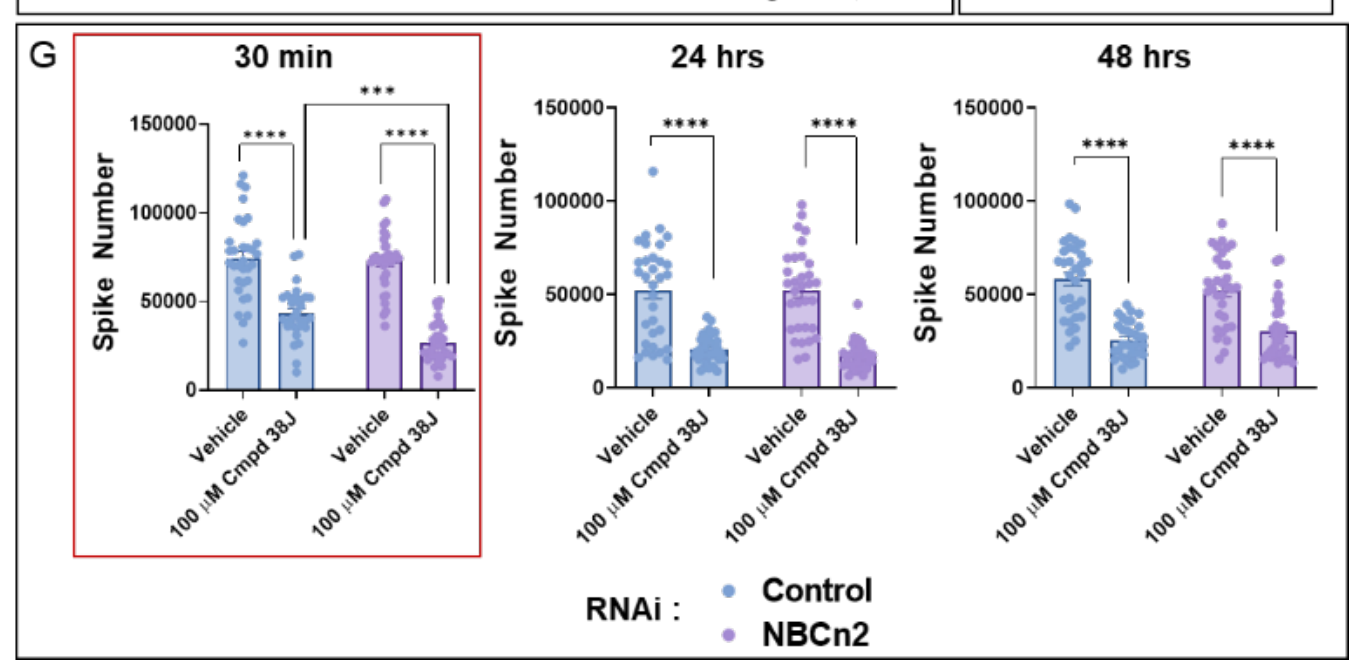

Fig E | qPCR monitoring expression of NBCn2, NBCn1, and NDCBE
Fig F | Neuronal activity in NBCn2 knockdown primary culture treated with RNAi measured by MEA assay
Fig G | Neuronal activity in primary culture following RNAi and drug treatment measured by MEA assay

We suspect that Cmpd38J-mediated reduction of neuronal activity is likely caused by inhibition of NBCn2, NDCBE, and NBCn1 in our neurons, as is indicated by its inhibitory effect on NDCBE (Fig A). We thus performed qPCR experiments and validated that NDCBE and NBCn1 are also expressed in our neuronal culture (Ct<30), though relative gene expression levels cannot be quantified due to probe dependence in qPCR experiments. Strikingly, we find that treatment with NBCn2 antisense RNA increases the expression levels of both NDCBE and NBCn1 (Fig E), which could be a compensatory reaction to NBCn2 knockdown.

Overall our findings suggest several conclusions:

1. Primary neurons express NDCBE, NBCn1, and NBCn2, but exact protein levels have to be determined in future studies.
2. Knockdown of NBCn2 alone has no effect on neuronal activity, likely due to i) NDCBE and NBCn1 contributing to redundancy of SLC4-mediated regulation of excitability, and ii) increased expression of NDCBE and NBCn1 following NBCn2 knockdown.
3. Cmpd38J reduces neuronal activity in all conditions, likely via inhibition of all three electroneutral SLC4 transporters (and potentially unknown targets of Cmpd38J).
4. NBCn2 knockdown and Cmpd38J additively reduce neuronal activity 30 min following drug treatment, which could be due to Cmpd38J inhibiting NDCBE, NBCn1, and NBCn2, thereby strengthening the impact of NBCn2 knockdown on neuronal activity.
5. Additive effects of NBCn2 knockdown and Cmpd38J inhibition dissipate over time, which could be due to adaptive changes.

We have since included our data in Figures C-G in a new Figure 6 and Extended Data Figure 13, and rewritten this section of the manuscript (Lines 405-428) to reflect our new findings.

The cell assay conditions are unlikely to be as the authors report. It is not possible to maintain a solution of pH 7.4 and [HCO3] of 25 mM in room air. The relationship between pH/HCO3/pCO2 is described by the Henderson-Hasselbalch equation. If pH is indeed 7.4 then, without substantial atmospheric CO2 to hold it in equilibrium, the majority of the added HCO3 will dissipate as CO2 and only ~0.25mM will remain (another possibility is that pH is much greater than 7.4). Thus the assay is not performed under physiological conditions, nor is it a robust assay because [HCO3] is probably much lower than Kd for the transporter. The authors should carefully re-evaluate their findings if [HCO3] is a critical parameter for interpretation and they should also be clear in their methods that the assay solution contained <1 mM HCO3.

We agree with the reviewer that the HEK cell based assays are NOT performed under physiological conditions, and that it is unknown how much [HCO3] our solutions truly contain. That being said, the goal of this HEK-cell based assay was not to determine Ki values or determine activity under physiologically relevant buffer conditions. Instead, we aimed to establish and execute an assay that produces the maximal possible signal for robust compound screening in 96-well format. We have previously already performed experiments prepared with 25 mM [HCO3] at pH 7.4 for similar purposes at AE1 (PMID: 37679563). Data from our assay optimization shows that buffers prepared with lower than 25 mM show significantly reduced signal windows that are not conducive to compound screening in our medium/high throughput assay (Fig H) as we cannot supply CO2 in this system. We note that in our assay, increased [HCO3] concentration leads to a larger acid load (as indicated by a stronger reduction in BCECF fluorescence). This is likely the main cause of the increased signal window as NBCn2-expressing cells more rapidly and efficaciously import available [HCO3] to counteract the acid load.

H

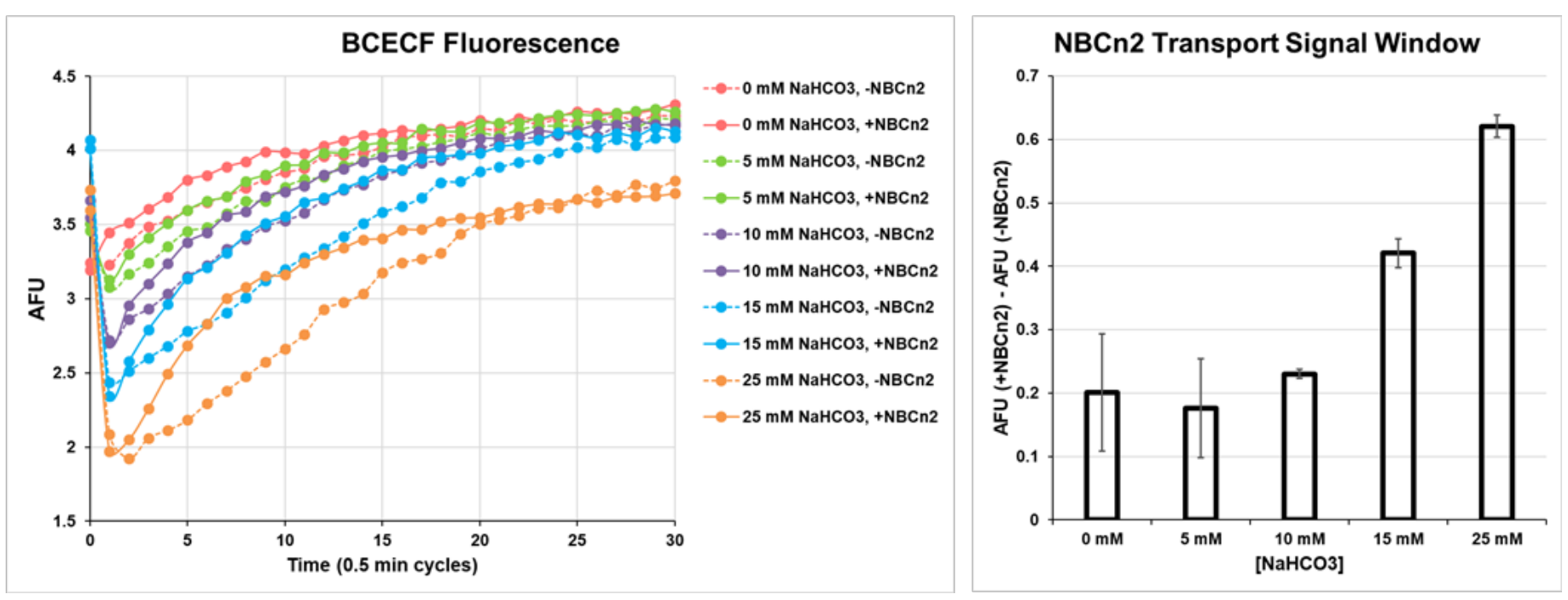

Fig H | Optimization of BCECF assay in NBCn2-expressing HEK cells

That being said, the reviewer's point still stands. In response, we have now removed any speculation on Ki ranges for our compounds, as this would require accurate Km values and determination of IC50 values with substrate concentrations closer to the Km in our assays. We further clarified in the methods that the indicated buffer conditions were necessary to obtain sufficient signal, but that uptake was likely done with substantially less than 25 mM [HCO3] (Line 733-737). We further point out in the discussion that "this assay is non-physiological and it is difficult to assess true affinities and potencies" (Line 520-521).

MINOR

Line 1: The use of 'at' in the title is awkward. Suggest rephrasing and suggest including the transporter name.

We have now replaced the title with "Structural studies and drug discovery targeting the neuronal NBCn2 carbonate transporter"

Line 53: '…other studies..' suggest replacing with '..an independent study..'

Done

Line 85: The authors should clarify whether removing 231-299 leaves a functional transporter.

We now clarify that modifications to the N-terminal domain unlikely affect function, as complete removal of this domain at other transporters leaves the function intact (Line 86-87). It should be noted that these modifications were only done for the cryoEM construct, and all functional experiments were performed with the full length transporter.

Line 458: What is the evidence for reversibility?

There is currently no evidence for this and we have since removed this point

Figure 6F-H: The statistics appear weaker than anticipated. Would the authors get a more robust outcome, or gain additional insight, by reporting (as a single metric) the drop in frequency of high amplitude events?

We appreciate the reviewer's suggestion, but the "weak" statistics misrepresent the robustness of the effect. We patched a total of 39 cells for the experiment (20 for vehicle and 19 for Cmpd38J conditions), and analyzed data recorded over 1 minute. Plots in our new Figure 7C and 7D (formerly Figure 6G and 6H) show mean amplitudes and mean frequencies over the course of 1 min of recording. Averaging of the data eliminates a lot of data. To more fully capture the effects of treatment with compound or vehicle beyond summary statistics, we analyzed the first 50 events recorded during the 1 min measurements for each cell. Figure I shows the data from this analysis that contains 1,000 cumulative events for vehicle (20 cells x 50 events) and 950 events for Cmpd38J (19 cells x 50 events). Frequencies cannot be recorded based on single events, which is why they have been replaced with Inter-event intervals (IEIs). Using a Kolmogorov-Smirnov test, we compared Amplitudes and IEIs and observe a highly significant drug/vehicle difference with P<0.0001 for both (Fig I). Since differences are difficult to see in this analysis, convention is to transform these data into histogram based cumulative probability plots, as was done in a cited previous study on mIPSC measurements in brain slices from wt and NBCn2 KO mice (PMID: 37459438). We previously already reported these data in Extended Data Figure 11, but have now moved these data into the main text as Figure 7E and 7F. We have now added our KS analysis of all recorded events by addition of "KS-test: ****" to both panels as we have done previously (PMID: 34135511). We have now clarified these aspects in the methods and the figure legend of Figure 7.

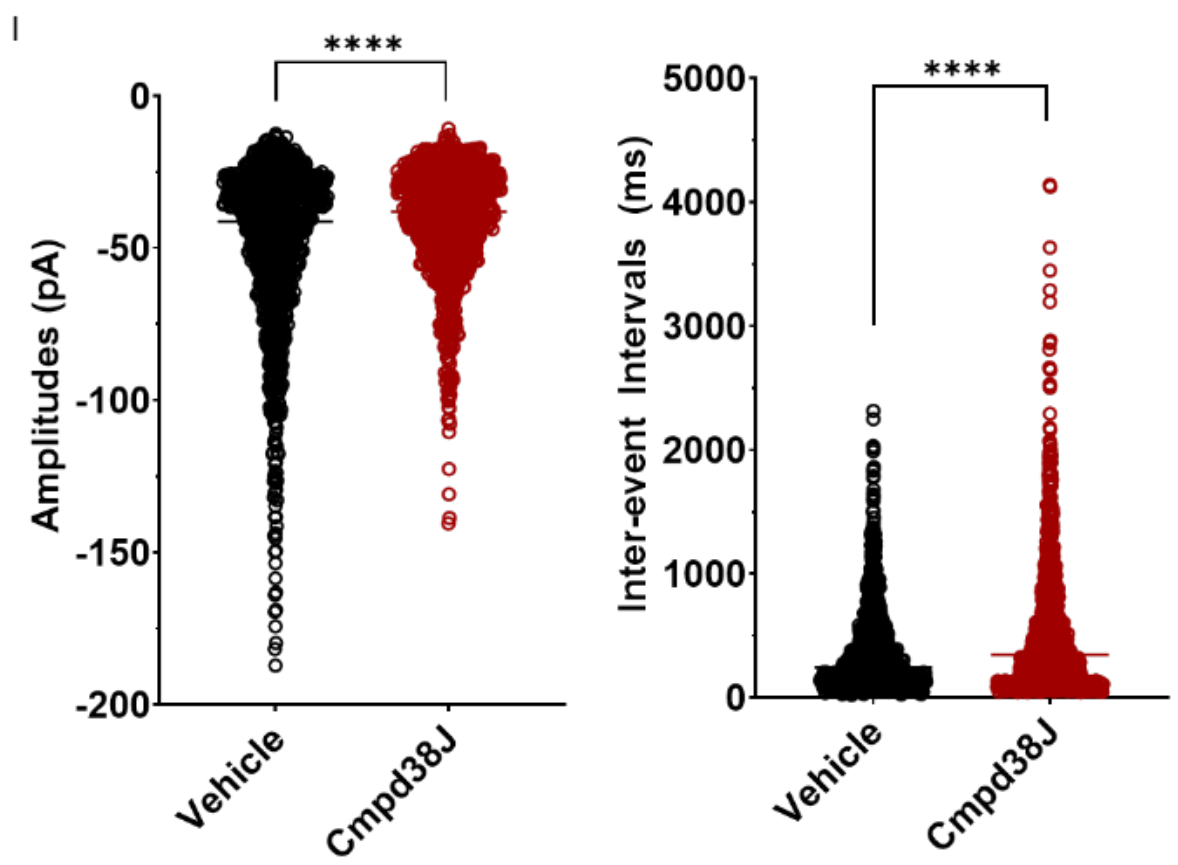

Fig I | Kolmogorov-Smirnov test based on 50 first recorded events from each patched cell.

Reviewer #3 (Remarks to the Author):

The authors present cryo-EM structures of the brain carbonate/Na+ symporter SLC4A10, highlighting determinants for substrate binding and transport, and further continue to screen for small molecule SLC4A10 inhibitors. Two lead compounds were further investigated, including substituent modifications, IC50 estimation by dose-response curves, and initial screening in physiological models, including neuronal culture and brain slices.

This is an important transporter for regulating intracellular pH and the insights into the structural basis for its mechanism and modulation by small molecules will be of great interest to the transporter community and has further potential in the development of anti-seizure medications.

Overall, this is a solid manuscript, but there are a number of clarifications and further experiments required to validate some of the main conclusions drawn.

Major points

Ion coordination

With only two oxygen atom interactions the coordination of the Na2 site is very unconvincing. Na+ coordination needs at least 5 ligands. Please consider: alchemical perturbation calculations, check-my-metal server, AlphaFold 3 prediction, ITC measurements of mutations and/or transport assays to validate sodium binding at the Na2 site.

We thank the Reviewer for this thoughtful comment and suggestion.

First, we would like to point out that, as is typically done for cryoEM structures, we only built the atoms/ions for which we obtained cryoEM density. We acknowledge that $Na^+(2)$ “in reality” likely has more than two interactions, but we did not observe any reliable density for additional ligands. This indicates that the additional water molecules, which likely make up the remaining ligands, are probably more delocalized. Because of that, we now performed computational experiments suggested by the reviewer, to provide further validation.

To evaluate the coordination environment of both $Na^+(1)$ and $Na^+(2)$ in our NBCn2 structure, we first analyzed the structure using the CheckMyMetal server. As shown in Fig. J, Na+(1) was found to form five metal-ligand interactions with a valence value of 0.9, categorized as “acceptable.” $Na^+(2)$ exhibited two coordination interactions (Fig. J), one with water (2.49 Å) and one with carbonate (2.57 Å), resulting in a valence score of 0.3, flagged as “dubious”.

To further explore the possible coordination environment of both sodium ions in an alternative approach, we used AlphaFold3 (AF3) to predict the structure of the NBCn2 monomer in complex with two sodium ions, one carbonate ion, and three water molecules. A total of 1,110 models were generated by using different random seeds, with five models generated per seed. Overall, the predicted protein structures were consistent, with RMSD values clustering around ~0.6 Å across most models (Fig. K). We excluded 28 of 1,110 models from the following ion coordinate and metal–ligand interaction analyses because their RMSD values exceeded 3 Å.

We used structural alignment and quantitative coordinate comparison to analyze the generated protein-ion complexes by AF3. Alignment of the top-ranked model with the NBCn2 cryo-EM structure revealed a highly conserved $Na^+(1)$ binding site (Fig. L, M, N). AF3 correctly placed both $Na^+(2)$ and carbonate within the substrate-binding pocket between TM3 and TM10, although they showed less precise positional agreement than $Na^+(1)$. Moreover, the relative positioning of both sodium ions and the carbonate ion was consistent with the cryo-EM structure. Quantitative analysis of ion xyz coordinates (Fig. N) further supported these observations. $Na^+(1)$ coordinates clustered tightly and closely matched the experimental structure. $Na^+(2)$ showed a tight cluster near the experimental coordinates with a limited number of clear outliers.

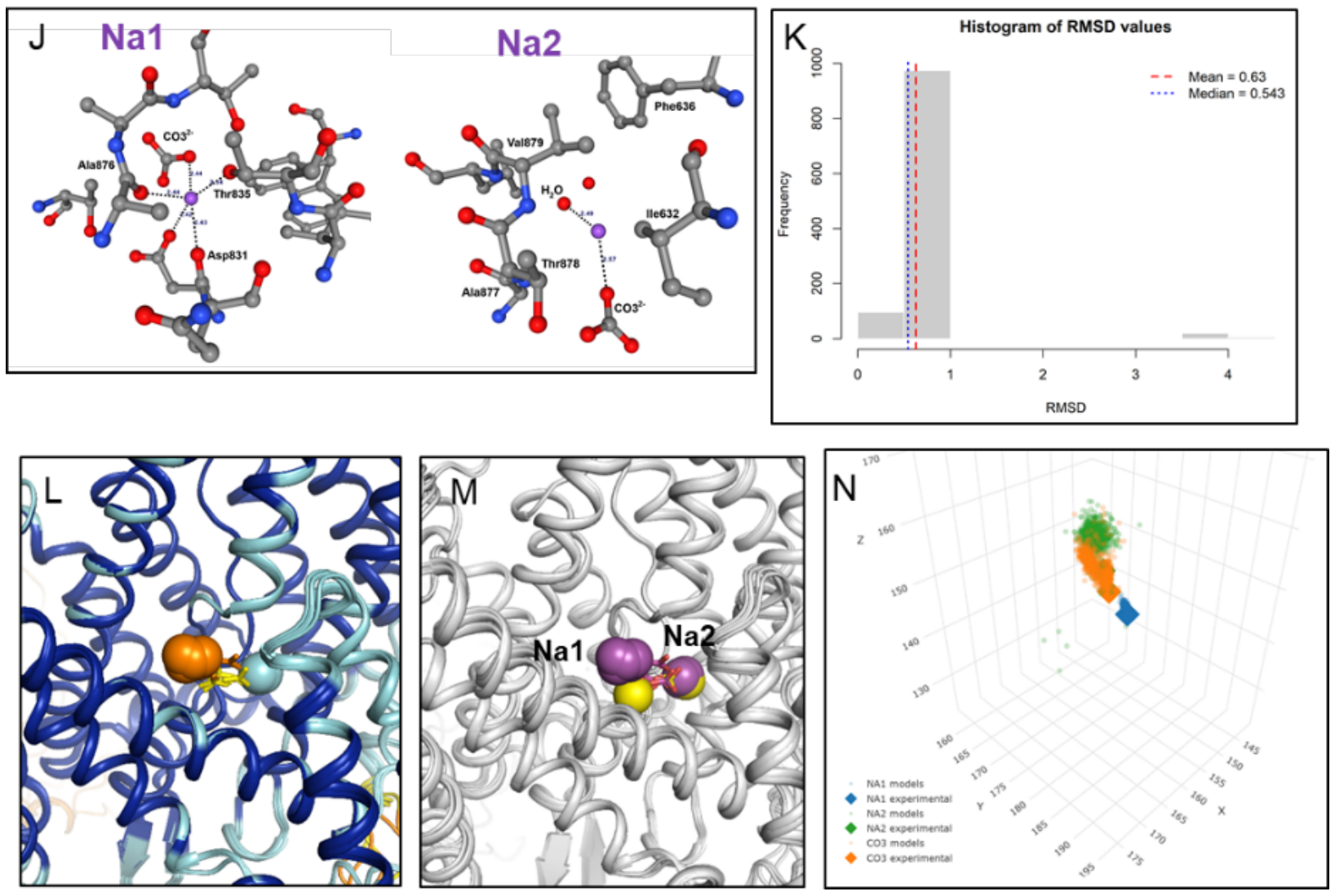

Fig J | Sodium-oxygen interactions of Na1 and Na2 according to check-my-metal server analysis
Fig K | RMSD distribution of 1,100 AF3 models relative to the cryo-EM structure.
Fig L | The five top-ranking AF3 models are superposed and colored by per-residue pLDDT values. Models are colored using AF3 color scheme.
Fig M | Alignment of the top five AF3 models sorted by ranking scores and cryo-EM structure. Experimental ions are shown in yellow, and predicted ions are shown in purple.
Fig N | Three-dimensional (xyz) coordinate distribution of ions from the cryo-EM structure and 1,072 AF3 models. Experimental coordinates are represented as diamonds, and AF3-predicted coordinates are shown as dots.

We further characterized the molecular interactions of both sodium ions and carbonate using Protein-ligand interaction (via PLIP) and distance measurements. As shown in Fig. O, AF3 accurately recapitulated the critical residues coordinating Na^+(1): Asp831, Ala876, and Thr835. Across all AF3 models, density plots of sodium–oxygen distances for these residues fell predominantly within the optimal coordination range of 2.4–2.5 Å. Carbonate also shows a strong density peak within the ideal coordination range for Na^+(1) (Fig. O). As previously shown in Fig. J, the CheckMyMetal server identified the same residues as Na^+(1) ligands in the cryo-EM structure, supporting the consistency between AF3 predictions and the experimental structure. Regarding metal-oxygen interaction of Na^+(2), as shown in Fig. O, we observe that across AF3 models, water molecules (HOH) show a strong density peak within the ideal coordination range, and carbonate (CO_3^{2-}) frequently forms sodium–anion interactions, sometimes within 3 Å. Importantly, AF3 predicts that Na^+(2) is most often coordinated by one to two oxygen atoms, predominantly from water and carbonate, with only occasional contributions from nearby protein residues such as Glu639 with flexible sidechain.

We acknowledge that Na^+(2) coordination is less canonical, and that the pLDDT values for Na^+(2), water, and carbonate are lower than Na^+(1) and protein, warranting caution in overinterpreting these predictions. However, it is noteworthy that AF3 consistently models Na^+(2) in the correct neighborhood of the observed site, forming interactions with the same molecules (carbonate and water) observed in our structure (Fig. L).

Together, these findings support our initial hypothesis that Na^+(2) binding in NBCn2 is mediated primarily by solvent and anion interactions. We have now added the data shown in Figures I-M in Extended Data Figure 7. We further discuss our results in Lines 169-183 to provide support for our observations from structural studies.

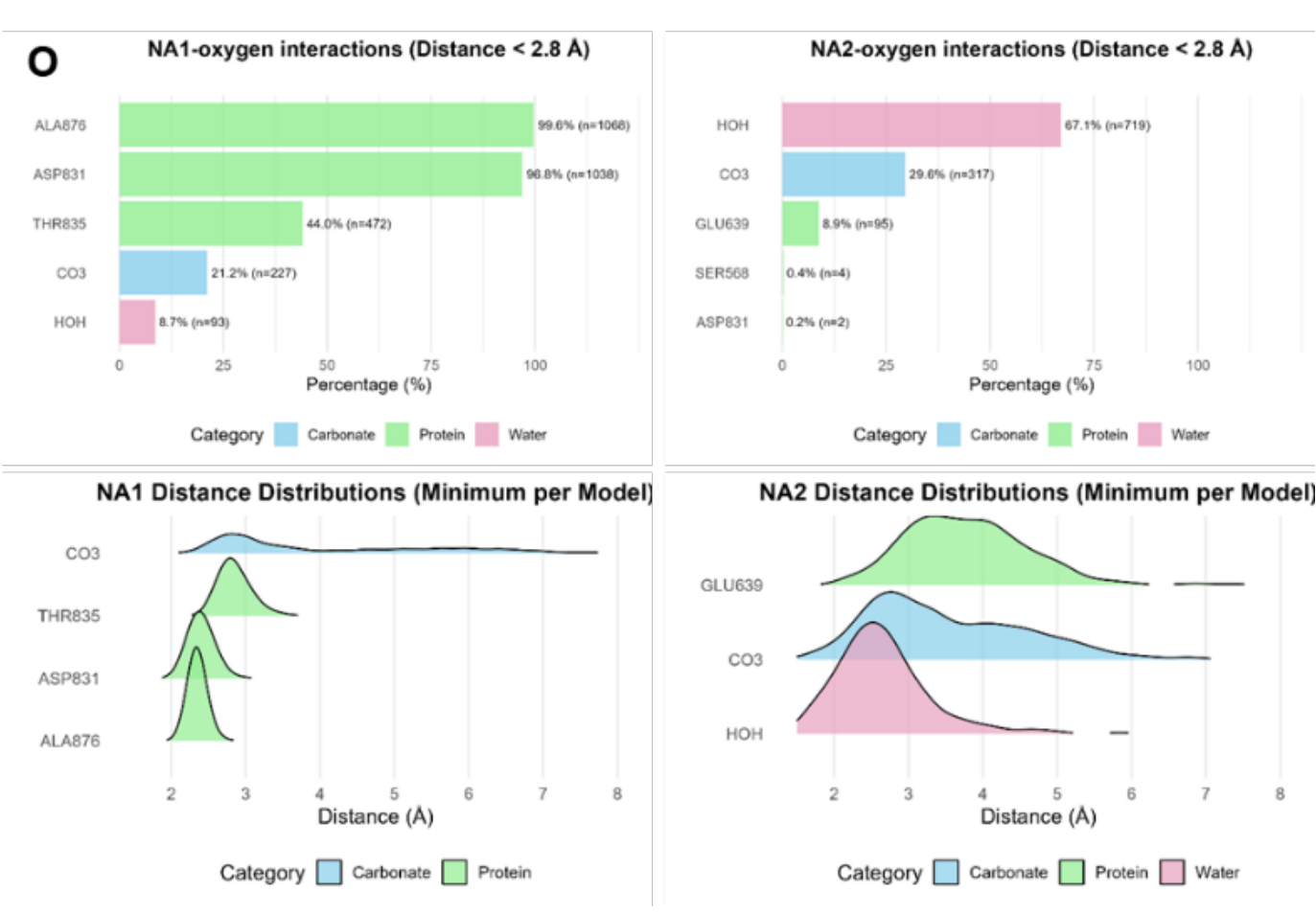

Fig O | Predicted Na1–ligand and Na2–ligand interactions across AF3 models. Percentage of AF3 models exhibiting Na^+(1) (Top Left) or Na^+(2) (Top Right) oxygen interactions within 2.8 Å, categorized by protein residues, carbonate, and water. Ridgeline plot of Na^+(1)–ligand (Bottom Left) and Na^+(2)–ligand (Bottom Right) interaction distances across AF3 models.

Inhibition studies

Please show the extent of inhibition of the small molecules vs. non-expressed protein rather than based on normalization to the compound38 (Fig. 4a). With the normalization currently carried out, its difficult to assess how much of the inhibition in cells might be due to a systemic off-target effect. The IC50 curves do not show a clear sigmoidal dose-response and further concentrations are needed for accurate fitting (Fig. 4b). While the cryo EM maps support the ligand binding of Cmpd 38J, the structural themselves are insufficient to prove that this is its mode of inhibition. Please mutate key coordinating residues to show either reduced or complete loss of inhibition by Cmpd 38J. It still was unclear to me if Cmpd 38J was specific to SLC4A10. Please show the IC50 to the other members NDCBE and AE1.

We thank the reviewer for these helpful suggestions. We have now changed the data in Figure 4A to represent uptake data normalized to vehicle control as was done in Figure 3 (Fig O). The concentration response data shown in Figure 4B indeed do not show a complete sigmoidal curve due to lack of data points at higher compound concentrations that should indicate a stable minimum uptake activity. Unfortunately, we are unable to collect such data since compound solubility in our uptake buffer becomes a problem above 100 µM, and increasing DMSO (vehicle) concentrations from our compound stock start to impact cellular health. Our uptake assay is very useful for medium/high throughput compound screening, as well as comparing relative potencies, but it is not suitable to accurately determine absolute potencies via Ki values due to the nature of the assay (indirect pH measurements, lack of control over substrate concentrations). We still suspect that Cmpd38J has higher affinity for NBCn2 than indicated by our IC50 estimates, but that will undoubtedly require orthogonal uptake assays (e.g. in liposomes). Such assays, however, have not been established for NBCn2 and would go well beyond the scope of our study that already shows compound-target interactions via structure, HEK cell transport, as well as neuronal activity data in primary culture and brain slices.

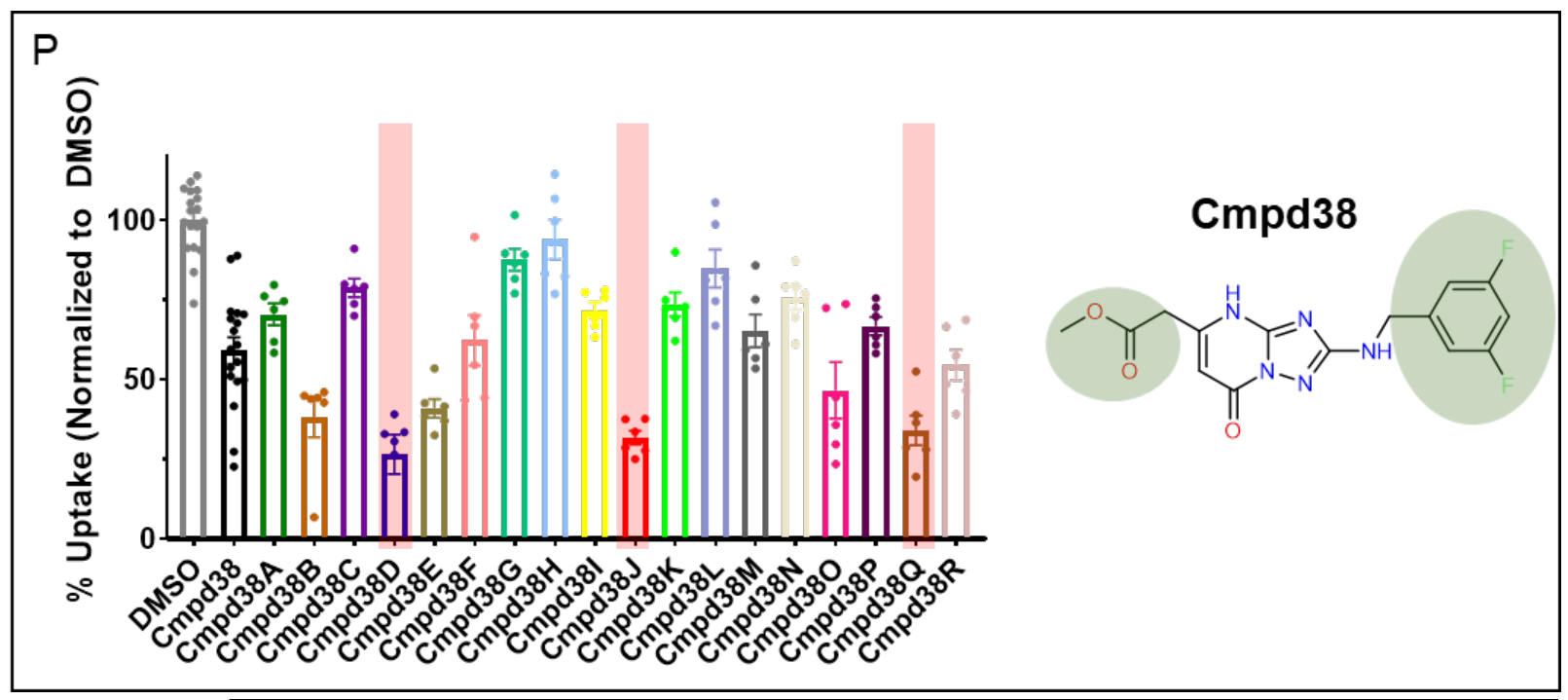

Fig P | Inhibitory activity of Cmpd38 Analogs normalized to DMSO. Compounds highlighted with red bars display inhibitory activity significantly different from both DMSO and Cmpd38 according to one-way ANOVA.

As requested by the reviewer we set out to characterize cmpd38J-NBCn2 interactions using site-directed mutagenesis. We mutated 6 key residues in the Cmpd38J binding pocket to alanine, and prepared stable HEK cell lines to perform uptake studies as was done for the wt transporter (Fig P). As we expected, most mutations entirely abolish NBCn2-mediated transport as Cmpd38J binds the substrate-binding pocket, and these residues likely also play a role in substrate binding, conformational changes required for substrate translocation, or both. The only notable exception is a K1001A mutation, which minimally reduced overall substrate transport, but appears to increase Cmpd38J's inhibitory efficacy at 100 µM concentration. These data partially agree with our MD studies that show that K1001 only minimally contributes to Cmpd38J binding (Fig. 5F, Extended Data Fig. 11C). To validate that the impact of the mutants on transport and Cmpd38J activity were not due to changes in transporter expression, we also performed ELISA (Fig Q). These data indeed confirm that none of the mutations impact NBCn2 expression. We have now included these data in Extended Data Fig. 12 and include a brief description and discussion of the data in the revised manuscript (Line 372-386).

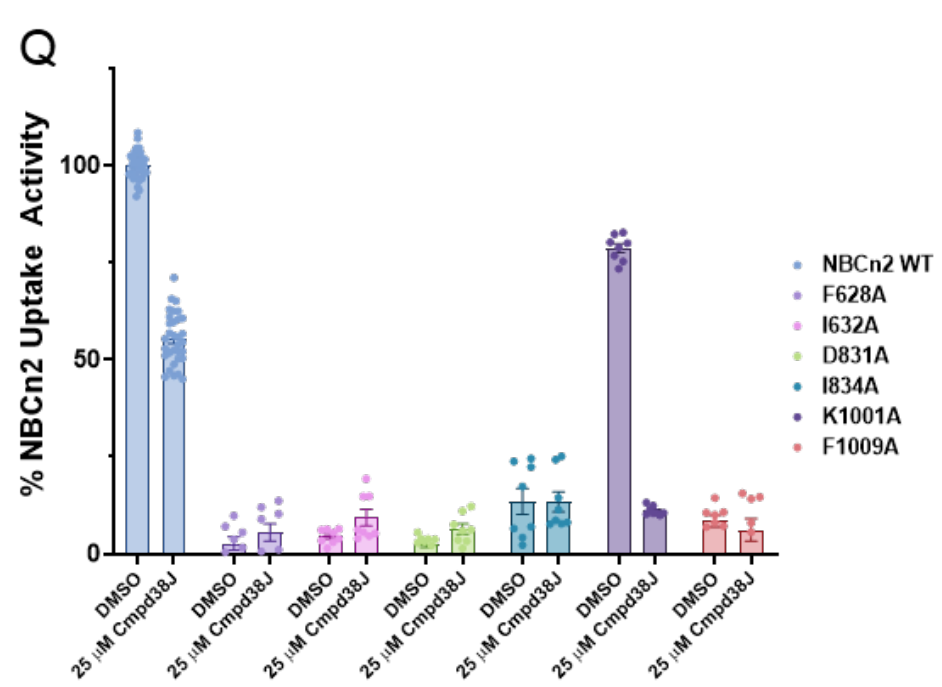

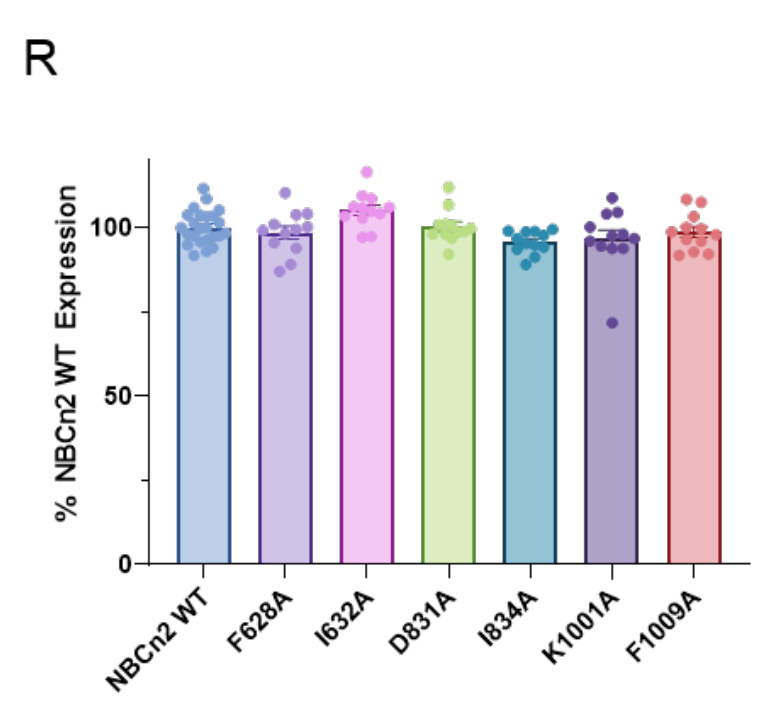

Fig Q | Normalized uptake activity of NBCn2 mutants.
Fig R | ELISA of NBCn2 mutants.

As requested, we have now also performed limited selectivity studies testing the activity of Cmpd38J at NDCBE and AE1, which was also requested by reviewer 2. As shown above (Figure A), Cmdp38J unsurprisingly also inhibits NDCBE, likely due to the highly similar binding pockets of NDCBE and NBCn2, but it does not show any activity at AE1. These data have now been included in the revised manuscript as Figure 4D.

Minor comments

- Lines 84-85: It would help to include a figure showing, based on the AF3 model, the construct used for structural studies and it's unclear to me the importance of the regions omitted.

As requested, we have now included the AF3 model for full length NBCn2 in Extended Data Figure 1, and colored areas of the model in red that have been truncated in the cryoEM construct. The truncated regions are all in the intracellular N-terminal domain, which typically does not play a role in SLC4-mediated transport. We have now stated that in the main text (Line 85-87).

- Lines 87-90 or Figure 1: it would be helpful to state that the structures obtained are in LMNG

We have now revised the legend of Figure 1 to read "**A**, CryoEM density of the overall NBCn2 homodimer obtained in LMNG detergent micelles".

- Lines 442-445: What are physiological substrate conditions that a drug would have to compete with?

Physiological serum HCO_3^- concentrations are typically in the range of 22-30 mM at a blood pH around 7.4. That being said, reviewer 2 rightfully points out that our effective HCO_3^- concentrations are likely considerably lower than the 25 mM used in our buffers. This is due to CO_2 dissipation, since we do not maintain HCO_3^- concentrations via consistent CO_2 bubbling (see comments to reviewer 2). We have thus removed any speculation about true potencies/affinities, and are currently working towards orthogonal assays that would allow to more accurately estimate affinities of Cmpd38J.

Reviewer #4 (Remarks to the Author):

I co-reviewed this manuscript with one of the reviewers who provided the listed reports. This is part of the Nature Communications initiative to facilitate training in peer review and to provide appropriate recognition for Early Career Researchers who co-review manuscripts.

We appreciate the reviewers' recognition of our revised manuscript. We further would like to thank Reviewer 3 for their additional insightful suggestions, which have allowed us to further improve the work. Our responses to these comments are provided below.

Major comments

Lines 375-386: The effect observed for K1001A is very interesting. It would make sense to see whether the effect of this mutation is due to an increased affinity for Cmpd38J or due to a different reason. A rudimentary IC50 determination would be helpful to be able to discuss this mutation in more detail, especially because this mutation/effect is not touched on again at all in the discussion.

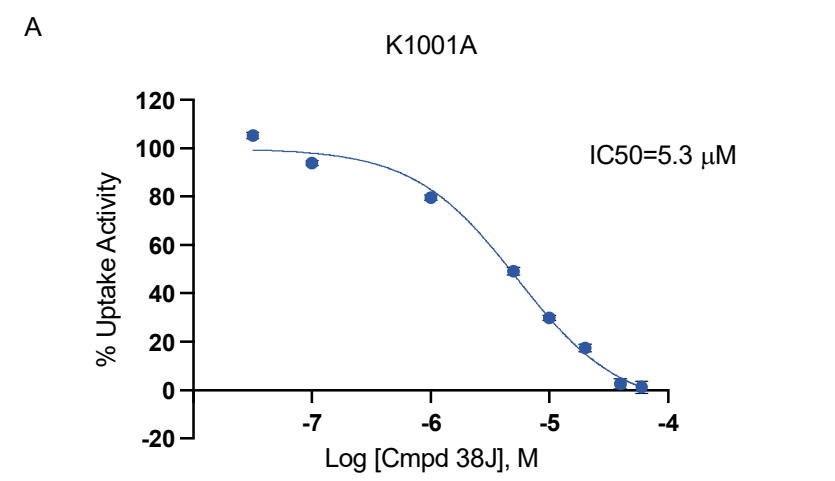

Fig. A | Concentration-response study of Cmpd 38J against K1001 mutant

We thank the reviewer for this suggestion, which we strongly agree with. To address this point, we have determined an IC50 for Cmpd38J at the K1001A mutant.

Consistent with our hypothesis, Cmpd38J shows a considerably increased apparent potency at the K1001A mutant (IC50=5.3 µM) compared to wild type (IC50=20.3 µM) (Fig. A). Consequently, we observe higher inhibitory efficacy at 25 µM Cmpd38J at the K1001A mutant compared to wild type NBCn2 (see Supplementary Figure 12B). We have now discussed in the text, that this effect is potentially due to the side chain of K1001 sterically interfering with Cmpd38J binding. Conceivably, K1001's polarity may also compromise hydrophobic areas of the binding pocket, thereby overall weakening the interaction with Cmpd38J. However, we note that a K1001A mutation could also affects substrate transport, thereby increasing the apparent potency of substrate transport inhibition. We have integrated a description and brief discussion of these findings into the main text (Lines 380-388) and included the data as Supplementary Figure 12D.

Lines 181-183: To make this statement, the same predictions/analyses without HCO3 and/or the waters should result in an unchanged Na1 site and the loss of Na2. This can be checked very quickly with AF.

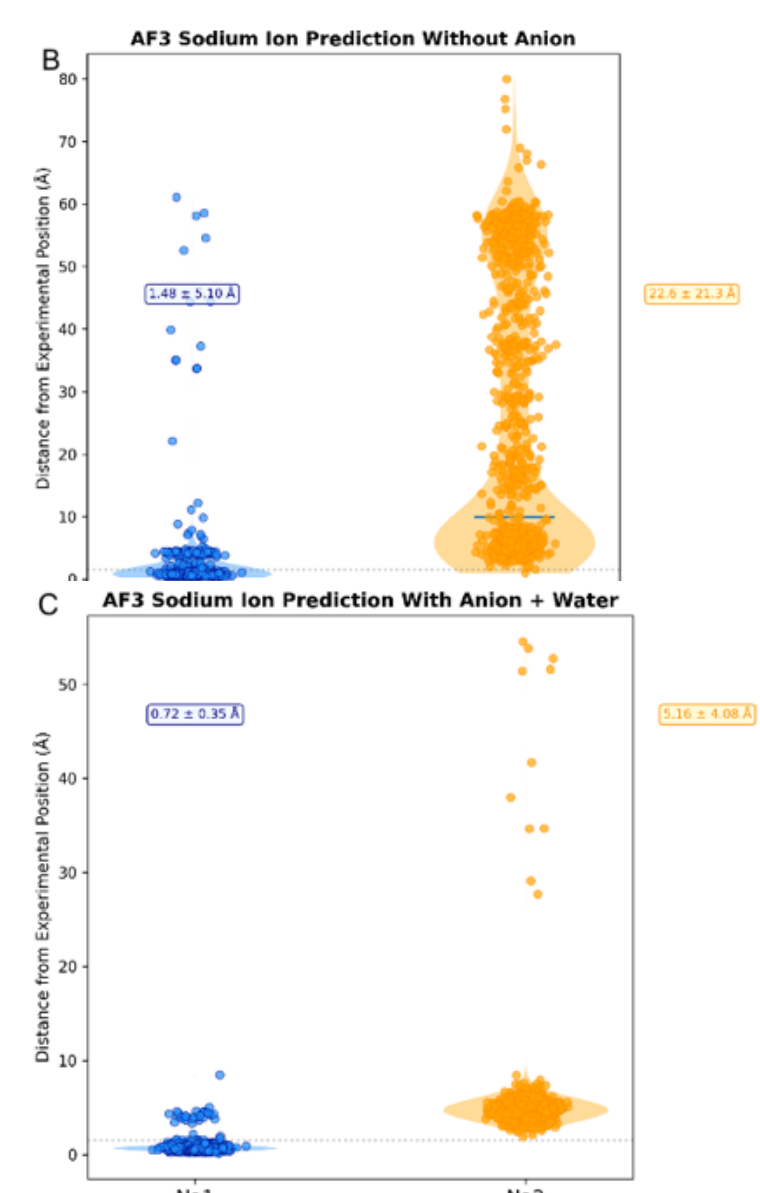

We thank the reviewer for this suggestion. In response, we performed additional AlphaFold3 (AF3) predictions of the NBCn2 monomer in complex with two sodium ions, excluding anions and water molecules. A total of 1,110 models were generated using different random seeds, with five models per seed. After alignment, the AF3 predictions were compared to the experimental cryo-EM reference positions of Na1 and Na2. The mean Euclidean distances (equivalent to the per-atom RMSD for a single atom) between predicted and observed sodium ion positions were calculated across the AF3 models. For comparison, we also re-analyzed our previously generated AF3 models of the NBCn2 monomer in complex with two sodium ions, one carbonate ion, and three water molecules using the same metrics.

As shown in Figure B, Na1 was still predicted at the correct site even in the absence of anions and water. A total of 938 out of 1,005 models (93%) placed Na1 within 1.5 Å of its experimental position (mean

displacement: 1.48 ± 5.10 Å). In contrast, Na2 was rarely predicted near its experimental site (mean displacement: 22.6 ± 21.3 Å). As shown in Figure C, Na2 localization improved substantially when AF3 was run in the presence of anions and water (mean displacement: 5.16 ± 4.08 Å), whereas Na1 showed a modest improvement under these conditions (mean displacement: 0.72 ± 0.35 Å).

The AF3 prediction implies that Na1 positioning is independent of the local chemical environment, whereas Na2 positioning depends on the presence of anions and water. This provides independent computational support for the functional distinction between the two sodium-binding sites observed in the cryo-EM structure, where carbonate and water were also observed. Accordingly we have now included this data (Supplementary Figure 7F, G) and a statement in Lines 178-182.

Figure 4A: I would consider making the bars greyscale and only coloring the bars for 38D, 38J, and 38Q, which will make it easier to highlight these particularly strong inhibitors.

Revised as suggested. Figure 4A now uses greyscale for background bars and highlights only 38D, 38J, and 38Q in color for emphasis.

Minor comments

Figure 1D the $NaHCO_3$ label is misaligned

We thank the reviewer for catching this. We have corrected the label alignment in Figure 1D.

Lines 310-313: I would add that this rather affects an accurate prediction of Cmpd38Js affinity rather than selectivity – you show that it is selective for NBCn2 and NDCBE over AE1

We have now edited this sentence to state that these confounding factors affect determination of accurate affinities/potencies (Line 310-313).

Throughout the manuscript: Misspelled propionate

Thank you for pointing this out. We have fixed the spelling in the revised manuscript.

Extended Data Figure 12 B: It would be easier to view this data with residue labels under the axis and two separate colors for DMSO/Cmpd38J. This would reduce the number of colors and make it easier to link the residues to the bars.

We have now updated Supplementary Figure 12B as requested, and also changed Supplementary Figure 12C to remove the colors.
